# The global epidemiology of chikungunya from 1999 to 2020: A systematic literature review to inform the development and introduction of vaccines

**DOI:** 10.1371/journal.pntd.0010069

**Published:** 2022-01-12

**Authors:** Alison A. Bettis, Maïna L’Azou Jackson, In-Kyu Yoon, J. Gabrielle Breugelmans, Ana Goios, Duane J. Gubler, Ann M. Powers

**Affiliations:** 1 The Coalition for Epidemic Preparedness Innovations (CEPI), Oslo, Norway; 2 The Coalition for Epidemic Preparedness Innovations (CEPI), London, United Kingdom; 3 The Coalition for Epidemic Preparedness Innovations (CEPI), Washington, D.C., Maryland, United States of America; 4 P95 Epidemiology and Pharmacovigilance, Leuven, Belgium; 5 Duke-NUS Medical School, Singapore; 6 Centers for Disease Control and Prevention (CDC), Fort Collins, Colorado, United States of America; Universidade do Estado do Pará: Universidade do Estado do Para, BRAZIL

## Abstract

Chikungunya fever is an acute febrile illness that is often associated with severe polyarthralgia in humans. The disease is caused by chikungunya virus (CHIKV), a mosquito-borne alphavirus. Since its reemergence in 2004, the virus has spread throughout the tropical world and several subtropical areas affecting millions of people to become a global public health issue. Given the significant disease burden, there is a need for medical countermeasures and several vaccine candidates are in clinical development. To characterize the global epidemiology of chikungunya and inform vaccine development, we undertook a systematic literature review in MEDLINE and additional public domain sources published up to June 13, 2020 and assessed epidemiological trends from 1999 to 2020. Observational studies addressing CHIKV epidemiology were included and studies not reporting primary data were excluded. Only descriptive analyses were conducted. Of 3,883 relevant sources identified, 371 were eligible for inclusion. 46% of the included studies were published after 2016. Ninety-seven outbreak reports from 45 countries and 50 seroprevalence studies from 31 countries were retrieved, including from Africa, Asia, Oceania, the Americas, and Europe. Several countries reported multiple outbreaks, but these were sporadic and unpredictable. Substantial gaps in epidemiological knowledge were identified, specifically granular data on disease incidence and age-specific infection rates. The retrieved studies revealed a diversity of methodologies and study designs, reflecting a lack of standardized procedures used to characterize this disease. Nevertheless, available epidemiological data emphasized the challenges to conduct vaccine efficacy trials due to disease unpredictability. A better understanding of chikungunya disease dynamics with appropriate granularity and better insights into the duration of long-term population immunity is critical to assist in the planning and success of vaccine development efforts pre and post licensure.

## Author summary

Chikungunya disease is a mosquito-borne viral infection which causes an acute febrile illness often associated with debilitating polyarthralgia. It is estimated that over three quarters of the world’s populations live in areas at-risk of chikungunya virus transmission and to date, no efficacious medical countermeasures exist. To guide vaccine development against chikungunya, data regarding where and when outbreaks occur are needed. We conducted a systematic literature review to describe the global epidemiology of chikungunya to inform vaccine development. We used well-defined methods to search for and identify relevant research published between 1, January 1999 and 13, June 2020 in MEDLINE and other publicly available sources. We reviewed 371 references which emphasized the global expansion of chikungunya since its reemergence in 2004. Gaps in epidemiological knowledge identified included the population at risk, magnitude of outbreaks, and duration of natural immunity. This information is essential for late-stage development of chikungunya vaccines.

## Introduction

Chikungunya is a mosquito-borne viral disease that has undergone widespread geographic expansion in the past 15–20 years [1]. The causative pathogen, chikungunya virus (CHIKV), was first isolated in 1952–53 during an outbreak in southern Tanzania by Lumsden and colleagues [2], however clinical descriptions suggest that chikungunya outbreaks possibly date as far back as the 1600s [3,4]. CHIKV autochthonous transmission has been reported to occur in 114 countries and territories over the tropical and sub-tropical areas of Africa, Asia, Oceania, the Americas and Europe in which over three quarters of the world’s populations live [5].

Onset of illness usually occurs approximately 4–8 days following a bite by an infected mosquito. The World Health Organization (WHO) defined three acute clinical forms for CHIKV infection (i.e., acute, atypical acute, and severe acute) and a chronic form [6]. Chikungunya is characterized by rapid onset of high-grade fever, frequently accompanied by arthralgia, primarily of peripheral joints. Other common signs and symptoms include myalgias, joint swelling, headache, nausea, severe fatigue, and maculopapular skin rash. Joint pain is often incapacitating and although the majority of cases recover within several weeks, joint and musculoskeletal pain may last for months to years post infection [7,8]. It has been reported that only 3%–25% of CHIKV infections remain clinically inapparent [9]; however, a more recent study reported a 49% probability of inapparent CHIKV infection [10].

CHIKV is an alphavirus (family *Togaviridae)* transmitted to humans by mosquitoes of the *Aedes* genus. While transmission in West and Central Africa is historically characterized as enzootic, involving several *Aedes spp*. mosquitoes and non-human primates, current transmission is increasingly associated with urban or peri-urban areas and likely sustained in the human population [11]. *Ae*. *aegypti* is the most common urban epidemic vector, though adaptation to other species, such as *Ae*. *albopictus*, has been described since 2007 [11,12]. This adaptation to new vectors, continued expansion of the mosquito vectors to more temperate climates, unplanned urban growth resulting in dense human populations, and spread of viruses via modern, rapid transportation have exposed an increasing proportion of the global population to the risk of chikungunya and other mosquito-borne diseases [13]. While the disease has been more frequently reported in tropical and subtropical regions, recent outbreaks raise concerns about the future public health impact of chikungunya in temperate regions [14], as reflected in the occurrence of an autochthonous outbreak in Italy in 2007 [15,16] and sporadic cases in France from 2010 [17].

Phylogenetic analyses reveal three genotypes of CHIKV: West African genotype, East/Central/South African (ECSA) genotype including the Indian Ocean Lineage (IOL), and the Asian genotype [11]. Evidence suggests that the Asian genotype evolved from the ECSA genotype sometime between 1879 and 1927 [18].

Given the epidemic potential and significant disease burden of chikungunya, the Coalition for Epidemic Preparedness Innovations (CEPI) is supporting development of CHIKV vaccines. Several vaccine candidates are currently in various stages of clinical development [19–21]. This systematic literature review aims to provide an in-depth analysis of the epidemiology of CHIKV infection over the last 20 years and to highlight current knowledge gaps and major challenges for assessing candidate vaccines and the feasibility to implement vaccine efficacy studies.

## Methods

A systematic review of the existing literature to describe the epidemiology of CHIKV was conducted in accordance with the Preferred Reporting Items for Systematic Reviews and Meta-Analyses (PRISMA) guidelines [22]. The protocol was registered on PROSPERO, an international database of prospectively registered systematic reviews in health and social care managed by the Centre for Reviews and Dissemination, University of York (CRD42020193856: https://www.crd.york.ac.uk/prospero/display_record.php?RecordID=193856) on 24 July 2020. The study aimed to describe CHIKV evolution (over time and space), outbreaks, seroprevalence, duration of infection-acquired immunity, and surveillance systems.

### Literature search

A literature search was conducted in MEDLINE (via PubMed) for articles published between January 1, 1999 and June 13, 2020. Limiting the search to studies published on or after January 1, 1999 was designed to capture major recent outbreaks while limiting potential bias resulting from differences in surveillance practices over time. The search string focused on incidence, prevalence, seasonality, risk factors, viral lineages, infection-acquired immunity, coinfections, seroprevalence, and surveillance systems; further details of the search strategy is provided in the **S1 Text**. Additional data available in the public domain, which matched the objectives and eligibility criteria for this study were retrieved from the WHO, Pan American Health Organization (PAHO), United States Centers for Disease Control & Prevention (US-CDC), European Center for Disease Prevention and Control (ECDC), Lilacs, African Index Medicus, Pro-Med, Scielo, and Google Scholar. For countries known to have chikungunya surveillance systems, Ministry of Health (MoH) websites were searched to identify online national surveillance reports. A full list of websites, data sources, and search dates and the completed PRISMA checklist are available in **S1 Table and S1 PRISMA Checklist**.

### Eligibility criteria

Inclusion criteria for this study were defined based on the Patient/Problem/Population, Intervention, Comparison/Control/Comparator, Outcome(s), and Study type (PICOS) strategy [23]. Observational studies addressing CHIKV infection or disease, conducted in any region and based on the general population or in any age group(s) were included. Studies were excluded if they focused exclusively on imported cases of chikungunya, pertained to diagnostic development, health economics, or social sciences, were case reports/series or authors’ opinions, were reviews or other studies not reporting primary data, included only non-human data, were published before January 1, 1999, or were reported in languages other than English, Spanish, Portuguese, or French. Although excluded, reference lists of relevant review papers retrieved from the electronic database search were reviewed to further identify potential studies of interest.

### Study selection and data extraction

Two reviewers independently screened the title and abstract of publications identified by the search using Rayyan software [24]. In a second step, the selected publications based on titles/abstracts were imported back into EndNote, and full texts were retrieved. Full texts and reports were then assessed for eligibility, by checking if they addressed the main outcomes of interest. As a quality control, two additional independent reviewers examined a random sample of 10% of the entries, and no discrepancies were found.

A pre-piloted standardized extraction form was used to extract data on study conduct (study design, population, country/region, study dates, case definitions, sample size) and outcomes (stratification variable, outcome type, outcome value, viral lineage, other co-circulating viruses). Data extraction was conducted by a single reviewer; data from 10% of included papers were independently re-extracted by a second reviewer for quality control purposes. Data were extracted as reported. Where possible, data were classified into pre-defined categories.

The following definitions were used to define chikungunya cases:

- Lab-confirmed: laboratory test performed to detect CHIKV infection (any type of serologic or virological test).
- Suspected: clinical diagnosis or clinical suspicion, generally raised by fever and arthralgia, with or without epidemiological link, but with no confirmed laboratory test result.

Studies and reports were also classified as:

- Outbreak data: if they addressed an outbreak limited in time or specifically mentioned to be an outbreak report.
- Seroprevalence data: if they provided rates of IgG positivity or if they specifically reported using the term “seroprevalence”.

The country groupings are based on the geographic regions defined under the Standard Country or Area Codes for Statistical Use (known as M49) of the United Nations Statistics Division.

### Descriptive analyses and synthesis of results

Data were initially summarized in tabular form by outcome using R 4.0.0 (R Core Team, 2020), retaining only those references that included data relevant for each outcome. The resulting summary tables were exported to Excel, and data were then manually inspected and further summarized. For the outbreaks table, data were summarized by outbreak. Attack rates and annual incidence rates were reported as a single measure because the reported attack rate periods were generally below or approximately equal to one year. Data on surveillance systems were summarized in tabular form. Reports that presented seroprevalence data were summarized in a table by reference and study region, with information on their study design, population, and sampling strategy. As this is a descriptive review and included studies were heterogenous in terms of methodology and thus difficult to directly compare, a meta-analysis was not performed.

## Results

### Study selection and characteristics

**Fig 1:**
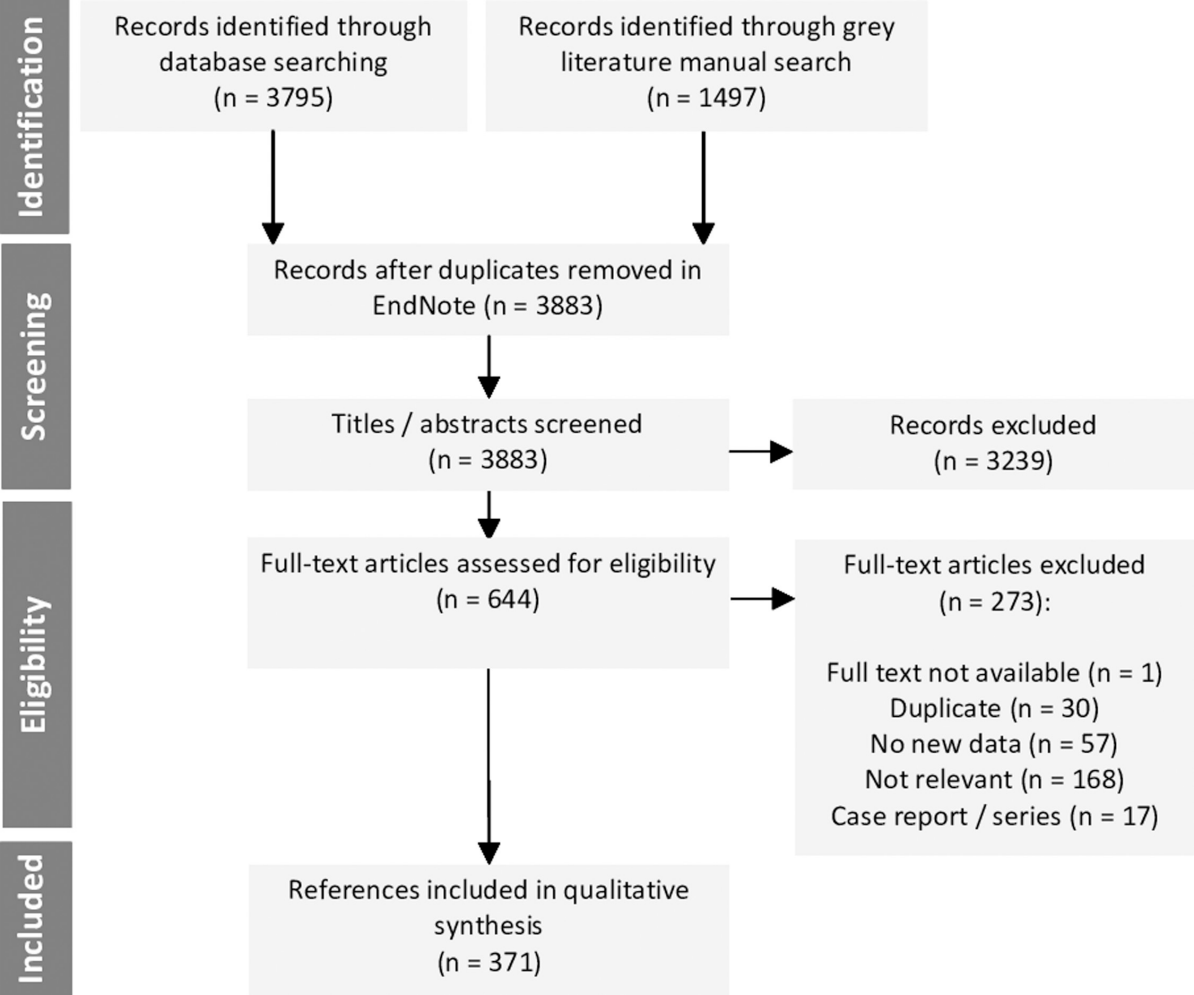
Flow diagram of the literature search according to PRISMA. All references identified in the online database searches were assigned a unique identification number. Following title and abstract review, duplicates were removed and articles further excluded based on the following inclusion/exclusion criteria: 1) Studies published in English, French, Spanish or Portuguese were included. 2) Studies addressing chikungunya virus infection or disease, conducted in any region, based on the general population or in any age group(s) were included. 3) Surveillance data from grey literature sources were also included. 4) Studies/reports focusing exclusively on imported cases of chikungunya, studies pertaining to diagnostic development, health economics, social sciences, case reports/series, author opinions, reviews or other studies not reporting on primary data, and non-human data were excluded.

There were 5,292 publications identified between January 1, 1999 and June 13, 2020, of which 3,883 were unique after removal of duplicates. Of the 3,883 unique records, 3,239 were excluded based on the title and abstract alone. Only one full text could not be accessed online or by direct request to the author. Full texts of the remaining 643 were assessed, of which 371 met the eligibility criteria (**Fig 1**). The list of all included references is available in **S2 Table**.

**Fig 2:**
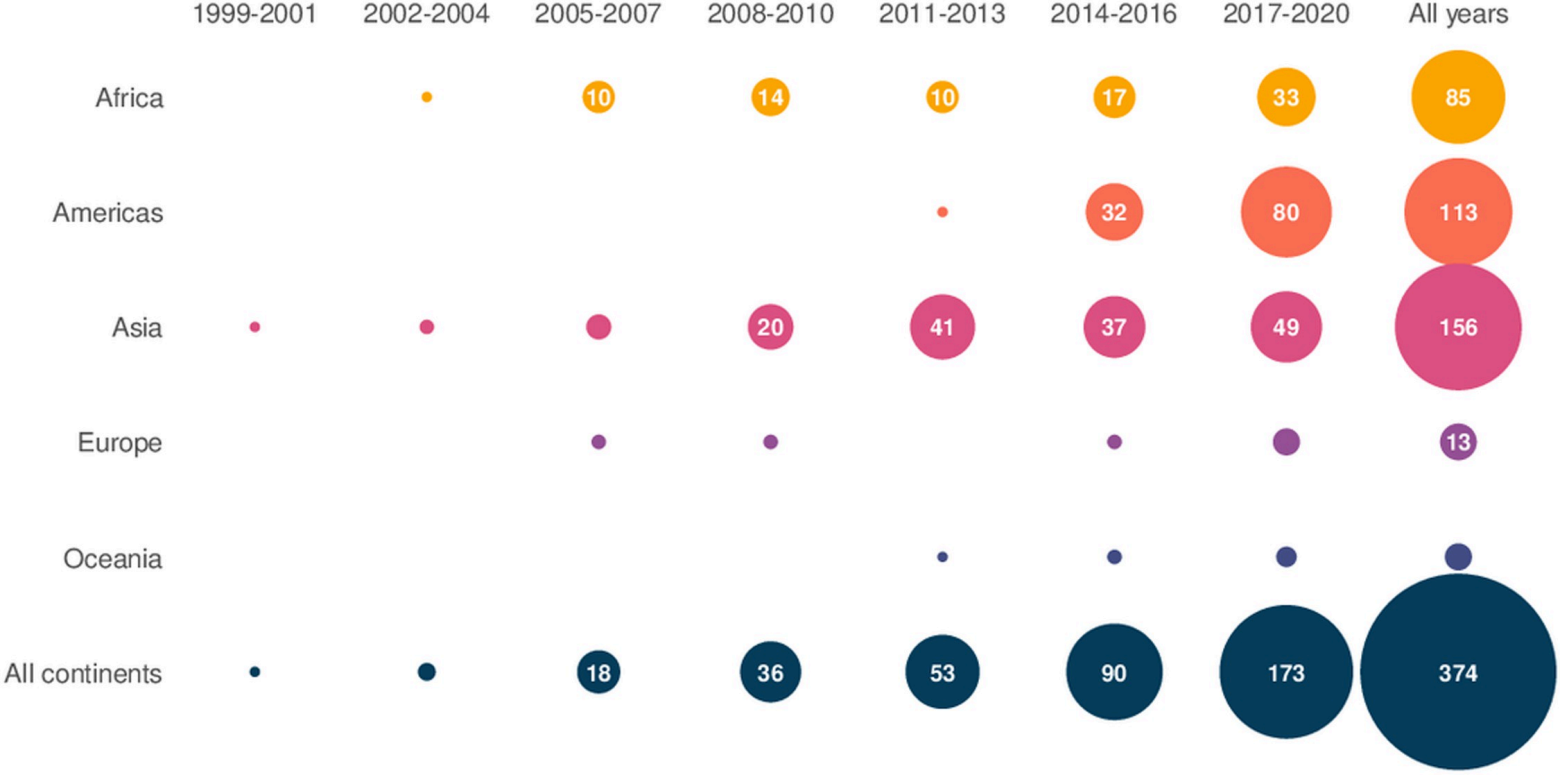
Number of references per year of publication and region of study setting. Circles are proportional to the number of studies. Circles with values lower than 10 are not labeled. Three references reported research in more than one region.

Out of 371 included studies, 173 (46%) were published from 2017 onwards. Of all included studies, 42% (n = 156) were from Asia, 30% (n = 113) from the Americas, 23% (n = 85) from Africa, 2% ((n = 7) from Oceania, and 3% (n = 13) from Europe (**Fig 2**). Three references reported research in more than one geographical area.

### Description of outbreaks/seroprevalence studies by geographic area

#### Africa

This review identified 13 chikungunya outbreaks in 11 African countries (**Table 1**) and 20 seroprevalence studies (**Table 2**) across 13 African countries between 1999 and 2020 Two countries experienced two different outbreaks during the study period; Gabon in 2006–7 and 2010, and Republic of Congo in 2011 and 2019 (**Table 1**).

**Table 1:** Chikungunya outbreaks by region and country, 1999–2020.

Region/Country	Area	Year of outbreak	Viral lineage
**Africa**			
Cameroon	West (Yaoundé and Douala)	2006	ECSA
Democratic Republic of Congo	Northwest (Kinshasa)	1999–2000	ECSA
Ethiopia	East (Dire Dawa)	2019	NR
Gabon	Northwest (Libreville and surroundings)	2006–07	ECSA
	Southeast (Franceville and surroundings)	2010	ECSA
La Reunion	La Reunion	2005–06	ECSA
Madagascar	East (Toamasina)	2006	NR
Mayotte	Mayotte	2005–06	NR
Republic of Congo	South (Brazzaville)	2011	ECSA
	West (Diosso)	2019	ECSA
Senegal	Southeast (Kedougou)	2009	West African
Sierra Leone	South (Bo)	2012–13	NR
Sudan	East (Kassala)	2015	NR
**Americas**			
Barbados	Southwest (Bridgetown)	2014	NR
Brazil	North (Amapá)	2014–15	Asian Urban
	Northeast (Sergipe)	2014–16	ECSA
	Northeast (Feira de Santana and Riachão do Jacuípe, Bahia)	2015	ECSA
	Southeast (Rio de janeiro)	2015–16	Asian Urban
	Northeast (Ceará)	2015–17	NR
	Northeast (Piauí)	2016–17	ECSA
	Northeast (Salvador, Bahia)	2017	ECSA
Colombia	North (Corozal & Ovejas, Sucre)	2014–15	NR
	North (Piedecuesta, Santander)	2014–15	NR
	Colombia	2015	NR
Dominica	Dominica	2013–14	Asian Urban
Dominican Republic	Southeast (La Romana)	2014	Asian Urban
Grenada	Grenada	2014	NR
Haiti	West	2014	NR
Honduras	Honduras	2015	NR
Martinique and Guadeloupe	Martinique and Guadeloupe	2013–15	Asian Urban
Mexico	South (Chiapas)	2014	Asian Urban
	Southeast (Yucatan)	2015–16	NR
Nicaragua	West (Managua)	2014–16	Asian Urban
Puerto Rico	Puerto Rico	2014	NR
Saint Martin /Sint Maarten	Saint Martin /Sint Maarten	2013–14	Asian Urban
Suriname	North (Paramaribo and Commewijne)	2014–15	NR
U.S. Virgin Islands	U.S. Virgin Islands	2014–15	NR
Venezuela	North (Aragua)	2014	NR
**Asia**			
Bangladesh	Bangladesh (Shibganj, Char Kusahi, Gopalpur)	2011–12	Indian Ocean
	East (Dhaka)	2017	ECSA
Bhutan	Southwest (Samtse, Chukha, and Thimphu)	2012	ECSA
Cambodia	South (Trapeang Roka, Kampong Speu)	2012	ECSA
China	South	2010	Indian Ocean
India	India	2005–06	Indian Ocean
	Southeast (Mallela, Andhra Pradesh); South (Avadi, Tamil Nadu)	2005–06	NR
	Southeast (Andhra Pradesh), Southwest (Karanataka), West (Maharashtra)	2005–06	ECSA
	West (Malegaon, Maharashtra)	2006	NR
	West (Sholapur, Maharashtra)	2006	NR
	South (Chennai, Tamil Nadu)	2006	NR
	East (Orissa)	2006	NR
	North (Delhi; Haryana; Uttar Pradesh)	2006–10	ECSA
	South (Kerala)	2007	ECSA
	East (West Bengal)	2007	NR
	Northeast (Assam)	2008	NR
	Southwest (Dakshina Kannada, Karnataka)	2008	Indian Ocean
	South (Kerala and Chennai, Tamil Nadu)	2008–09	ECSA
	Southeast (Andhra Pradesh)	2008–09	ECSA
	North (Delhi)	2010–11	NR
	North (Gwalior, Madhya Pradesh)	2010	ECSA
	Northeast (Garo Hills, Meghalaya)	2010	ECSA
	East (Orissa)	2010	Indian Ocean
	South (Tirunelveli, Tamil Nadu)	2010	ECSA
	Southeast (Medak, Telangana)	2013	NR
	East (Odisha, Orissa)	2013	ECSA
	East (West Bengal)	2014–15	NR
	Northeast (Assam)	2015	ECSA
	West (Pune, Maharashtra)	2016	ECSA
	North (Delhi)	2016	Multiple
	Central (Madhya Pradesh)	2016–18	ECSA
Indonesia	Southwest (Yogyakarta)	1999	NR
	Southwest (Bandung, West Java)	2000–08	Asian Urban
	Indonesia	2001	NR
	Indonesia	2010–11	NR
	North West (Sei Suka, North Sumatra)	2013	NR
	North West (Sumatra)	2014–15	Asian Urban
	South (Bali)	2015–16	Asian Urban
Lao PDR	South (Champassak)	2012	NR
Malaysia	Malaysia	2008–09	ECSA
	Malaysia	2010–11	NR
Maldives	Maldives	2006–07	NR
Nepal	South (Terai)	2013–15	NR
Pakistan	Pakistan	2016–17	Indian Ocean
Philippines	Philippines	2010–11	NR
	Philippines	2011–13	Indian Ocean; Asian Urban
	North (San Pablo, Laguna)	2012	NR
	Central (Cebu)	2012–14	Asian Urban
Thailand	South (Narathiwat)	2008–09	NR
	West	2010–11	NR
	Northeast (Bueng Kan)	2013	Indian Ocean
Vietnam	Vietnam	2010–11	NR
Yemen	West (Al-Hudaydah)	2010–12	Indian Ocean
**Europe**			
Italy	Northeast (Ravenna, Emilia-Romagna)	2007	Indian Ocean
	Central (Lazio)	2017	Indian Ocean
France	Provence-Alpes-Côte d’Azur	2010	NR
	Montpellier	2014	ECSA
	Provence-Alpes-Côte d’Azur	2017	ECSA
**Oceania**			
Federated States of Micronesia	Yap State	2013–14	NR

ESCA = East, Central and South African; NR = Not reported or not clear from the study text

**Table 2:** Seroprevalence data by region and country, 1999–2020.

Region/ Country	Area	Population^1^	Year sample collection	Recent outbreak^2^	Sample size	Seroprevalence (%)		Inapparent infection
						IgG	Overall IgM or IgG	(% of seropositives)
**Africa**								
Benin	South (Cotonou)	Pregnant women	2006–07	No	352	36.1	NR	NR
Cameroon	Northwest (3 sites, Kumbo)	Households	2007	Yes	105	88.6	NR	NR
Comoros	Comoros (Grande Comore, Ndzouani and Mwali)	Patients >15y	2011	No	400	12.0	NR	NR
	Grande Comore	Households	2005	Yes	331	26.9	63.1	NR
Kenya	West (Alupe)	Children aged 1-12y	2010–11	No	649	NR	5.6	NR
	East (Lamu Island)	Households, individuals >1y	2004	Yes	288	71.5	74.7	NR
	Kenya (West—Busia and Samburu—and East—Malindi)	Individuals >17y	2004	Yes	1141	34.0	NR	NR
	West (Busia)	Individuals >5y	2010–12	No	500	66.9	NR	NR
La Reunion	La Reunion	Pregnant women	2006	Yes	888	NR	18.2	NR
	La Reunion	Households	2006	Yes	2442	39.6	NR	NR
Madagascar	Central-East (6 sites)	Pregnant women	2010	Yes	1244	12.4	NR	25
Mayotte	Mayotte	Households	2006	Yes	1154	37.2	NR	28
Mozambique	Central-North (5 sites)	All patients	2015–16	No	392	28.6	NR	NR
	Mozambique	All patients	2009–15	No	895	17.9	NR	NR
Republic of Djibouti	Southeast (Djibouti city)	Households	2010–11	No	914	2.6	NR	NR
Rwanda	Rwanda	Blood donors	2015	No	874	63.0	NR	NR
Senegal	Northeast (5 sites)	Individuals >1y (nomadic pastoralists)	2014	No	1463	2.7	NR	NR
	Southeast (Kedougou)	Households	2012	No	998	54.0	NR	NR
Tanzania	Southwest (Kyela)	Patients >2y	2015	No	132	10.6	14.4	NR
Uganda	Uganda (5 sites)	Blood donors	2006–07	No	1744	31.7	NR	NR
**Americas**								
Brazil	North (Macapá, Amapá)	Blood donors	2015	Yes	442	0.2	NR	NR
	Southeast (Ribeirão Preto, São Paulo)	Blood donors	2016	Yes	455	0.0	NR	NR
	Northeast (Chapada, Bahia)	Households	2016	Yes	120	18.3	20.0	46
	Northeast (2 sites, Bahia)	Households, individuals >1y	2015	Yes	831	36.2	51.0	63
Martinique and Guadeloupe	Martinique	Blood donors	2015	Yes	1004	41.9	NR	NR
	Guadeloupe	Blood donors	2015	Yes	750	48.1	NR	NR
Mexico	Central (Puente de Ixtla, Morelos)	Households, Individuals >1y	2016	Yes	387	29.5	NR	43
Nicaragua	Managua	Children aged 2-14y	2014–15	Yes	3362	NR	6.1	58
	Managua	Households, individuals ≥15y	2015	Yes	848	NR	13.1	65
	Nicaragua (39 sites)	Households, individuals >2y	2015	Yes	11280	NR	32.8	19
Saint Martin	Saint Martin	All patients	2014	Yes	203	17.7	20.7	41
**Asia**								
India	South (Chennai, Tamil Nadu)	Households, individuals >4y	2011	Yes	1010	43.5	NR	60
	West (Sholapur, Maharashtra)	Households	2006	Yes	1192	30.4	NR	NR
	South (Kerala)	Households, individuals >13y	2009	Yes	381	68.0	NR	13
Iraq	South (Nasiriyah)	Individuals >13y, patients >9y	2012–13	No	399	0.5	NR	NR
Malaysia	Central	Individuals >34y	2008	No	945	5.9	NR	NR
Qatar	Qatar	Blood donors, male ≥18y	2013–16	No	200	3.5	NR	NR
Singapore	Singapore	Individuals >17y	2010	Yes	3293	2.2	NR	NR
Thailand	Central + South	Patients >6m	2014	No	835	26.8	NR	NR
	South (Songkhla)	Pregnant women	2009–10	Yes	319	45.7	72.3	NR
	South (Phatthalung)	Households, individuals >17y	2010	Yes	507	61.9	NR	47
**Europe**								
Turkey	Central (Kirrikale, Central Anatolia)	Blood donors	2015	No	500	0.4	NR	NR
Croatia	Southwest (Adriatic Coast)	Individuals >2y	2011–12	No	1008	0.9	NR	NR
Italy	Castiglione di Cervia	Households	2007–08	Yes	325	10.2	NR	18
Sweden	Sweden	Blood donors	2015	No	199	8.5	NR	NR
**Oceania**								
Fiji	Fiji	Individuals >5y	2017	No	320	12.8	NR	NR
French Polynesia	Tahiti	Children (primary/ secondary school)	2014	No	476	1.0	NR	NR
	French Polynesian (5 sites)	Households	2014	No	196	3.0	NR	NR
	Society Islands	Households	2015	Yes	700	76.0	NR	13
Solomon Islands	Honiara and Gizo	Households	2016	No	188	0.8	NR	NR

^1^“Households” and “all patients” includes children and adults, unless otherwise specified.

^2^Recent outbreak refers to studies conducted during or immediately after an outbreak.

M = Month; NR = Not reported or not clear from the study text; Y = Year

The first reported outbreak within the study period occurred in Democratic Republic of Congo (DRC) from May 1999 to February 2000 and was associated with the ECSA genotype [25]. See **Table 1** for details regarding the chronology of CHIKV outbreaks and associated genotypes. Chikungunya in Africa has historically been characterized by smaller-scale outbreaks associated with spillover from the natural reservoir vectors, *Aedes Stegomyia spp* mosquitoes, into humans in proximity to sylvatic transmission cycles. However, recent years have seen an increase in larger-scale outbreaks associated with urban areas of Africa as well (e.g., Ethiopia in 2019 [26], La Reunion in 2005–06 [27], or in Gabon in 2006–07 [28]). Data on incidence or attack rates for outbreaks identified in Africa are limited to four reports, with incidence highest in La Reunion 2005–06 (3,400 suspected cases per 10,000 inhabitants) and lowest in Mayotte (400 suspected cases per 10,000 inhabitants) [29] (**S3 Table**). Rather than relying solely on clinically identified cases, the majority of outbreaks in Africa reported lab-confirmed cases with the exception of Mayotte [29], although in variable proportions. Eight of the 13 outbreaks evaluated and identified in Africa reported the viral lineage and all but one were associated with the ECSA genotype; only the outbreak in Senegal in 2009 reported the West African genotype. Co-circulating mosquito-borne pathogens were reported in 10 outbreak investigations, mostly dengue virus (DENV) but also yellow fever virus (YFV), Zika virus (ZIKV), Rift Valley Fever virus (RVFV), West Nile virus (WNV), Sindbis virus (SINV), and malaria (**S3 Table**).

The proportion of seropositive individuals in African countries ranged from 3% (Republic of Djibouti 2011 and Senegal 2014) [30,31] to 89% (Cameroon 2007) [32] (**Table 2**). Globally, the highest seroprevalence values were detected in studies carried out during or shortly after an outbreak, although high values in this analysis were also detected in regions where no outbreak was reported (e.g. East Kenya (72%) [33] and Rwanda [63%) [34]).

Of the 20 seroprevalence studies identified in Africa, two studies reported a proportion of inapparent infections among the identified seropositive individuals (Madagascar (25%) [35] and Mayotte (28%) [36]). Ten seroprevalence studies (50%) were clinic- or hospital-based (five focused on specific populations, such as blood donors, pregnant women, or children) and 10 (50%) were community-based (**S4 Table**). Twelve (60%) seroprevalence studies used convenience sampling.

Surveillance systems were identified in Gabon, La Reunion, and Mayotte (**S5 Table**). Gabon (active from 2007–2010) [37] and La Reunion (active since 2005) [27,38,39] each used/use a sentinel network of physicians and laboratories and included/include all age groups. Mayotte employs an all-age, regional, passive system using healthcare providers and hospitals (active since 2005), and a hospital-based surveillance system focused only on maternofetal cases (active since 2006) [40].

#### Asia

Fifty-three chikungunya outbreaks in 15 different countries (**Table 1**) and 10 seroprevalence studies across six countries (**Table 2**) were identified from 1999 to 2019. Six countries (Bangladesh [n = 2], India [n = 26], Indonesia [n = 7], Malaysia [n = 2], the Philippines [n = 4], and Thailand [n = 3]) reported multiple outbreaks during this time period; 26 outbreaks occurred in India alone across numerous geographic areas. India and Indonesia reported outbreaks nearly every year of the study period.

Chikungunya epidemiology in Asia during this review period was generally characterized by large-scale outbreaks followed by periods of little to no reported transmission in the same geographic area. The first recorded outbreaks during the study period took place in Indonesia, beginning in 1999. Following several decades without reported outbreaks, the 2005 introduction of the IOL of ECSA genotype to India (via the Indian Ocean islands) led to large-scale transmission and multiple outbreaks in India and southeast Asia over the next decade. While outbreaks continued to be reported from 2015–2017, this period signaled a decline in the overall number of outbreaks identified in Asia. **Table 1** presents the chronology of chikungunya outbreaks across the region and associated genotypes. The highest incidence rates were reported for outbreaks in India in 2006 (4,270 suspected cases per 10,000 inhabitants) [41] and in 2008 (6,644 suspected cases per 10,000 inhabitants) (**S3 Table**) [42]. Yemen and the Philippines reported lower rates (70–120 suspected cases per 10,000 inhabitants) [43,44]. However, only 12 out of 53 (23%) reports had available incidence data. The vast majority of outbreaks identified in Asia reported lab-confirmed cases, with the exception of two in India [45,46]. Overall, from the available data, Asian countries appeared to test a relatively high proportion of suspected cases identified during outbreaks (particularly in India), with the exception of Yemen [44] and one outbreak in western India [47] which only tested between 0.7–1.2% of suspected cases. It should be noted that 14 reports did not specify the number of suspected cases, and thus information on the overall proportion of suspected cases tested or lab-confirmed is lacking. Of the 53 outbreaks identified across 11 Asian countries, 30 specified a viral lineage. Most (n = 25) outbreaks in Asia were associated with the ECSA genotype (nine with IOL in 8 countries). The Asian lineage was only identified in two countries, Indonesia and the Philippines. Two outbreaks included co-circulation of multiple lineages in northern India in 2016 [48] and in the Philippines in 2011–13 [49], the only two countries reporting different genotype circulation. Co-circulating mosquito-borne pathogens were described in 12 Asian countries and 26 outbreaks, with DENV noted in all instances and a small number of other pathogens (Japanese Encephalitis virus (JEV), ZIKV, and malaria) also noted. Most identified Asian outbreak studies were hospital- or clinic-based (34 in total). Twenty-six of the outbreak studies involved a convenience sample.

In Asia, the highest proportions of IgG-positive participants were reported in southern India in 2009 (68%) [50] and southern Thailand in 2010 (62%) [51], while low seroprevalence (0.5–6%) was reported in countries where no outbreaks were identified (Iraq, Qatar and Singapore [52–54]). Three studies reported a proportion of inapparent infections among the identified seropositive individuals (ranging from 13–60%) [50,51,55] (**S4 Table**). Three serosurveys were clinic- or hospital-based (two focused in specific populations, such as blood donors [53] or pregnant women [56]), six were community-based, and one was both clinic- and community-based. Six of the serosurveys used convenience sampling.

Surveillance systems were identified in three Asian countries (**S5 Table**). Several systems are in place in India including an all-age national, active sentinel surveillance system [57], an all-age national, active lab-based surveillance system predominantly focused in urban areas (active since 2013) [58], and an all-age regional, active surveillance system using local health units [45]. Singapore employed an all-age national, active sentinel laboratory-based surveillance system (active in December 2006) as well as an all-age national, passive surveillance system (active in December 2008) [59].

#### Oceania

Only one outbreak in Oceania was identified through this review, in the Federated States of Micronesia with a reported incidence of 1,550 suspected cases per 10,000 inhabitants [60]. Lab-confirmed cases were reported, though a relatively low proportion (approx. 10%) of suspected cases were tested.

Five seroprevalence studies were identified and the overall proportion of participants that were seropositive in Oceania was low (ranging from 1–13%) except for one area in French Polynesia reporting high seropositivity (76%) as well as a proportion of inapparent infections among the seropositives [61] (**Table 2**). Of the five serosurveys identified in Oceania, all were community-based (with one focused on primary and secondary school children). No specific viral lineage was reported, and no co-circulating pathogens were described in the retrieved references for Oceania. No surveillance system specific to chikungunya was identified through the review.

#### The Americas

Twenty-five chikungunya outbreaks in 16 countries (**Table 1**) and 11 seroprevalence studies (**Table 2**) in 5 countries were identified in the Americas over the 1999–2019 period, all occurring since 2013. Three countries experienced multiple outbreaks during the study period (Brazil [n = 8], Colombia [n = 3], and Mexico [n = 2]). Chikungunya outbreaks in the region increased in frequency and geographic distribution from 2013–2018, after which there was a decline (Table 1).

Chikungunya epidemiology in the Americas during this review period was generally characterized by large outbreaks followed by periods of lower transmission in the same geographic region. Incidence data were available for approximately half (13/25) of the outbreaks. The highest incidence was reported in Suriname, 2014–2015, with 2,760 suspected cases per 10,000 inhabitants [62] (**S3 Table**). Most of the outbreaks reported lab-confirmed cases, although four relied solely on clinically identified cases: northeastern Brazil in 2015 [63], Colombia in 2014 [64] and 2015 [65], and Honduras in 2015 [66]. The overall proportion of suspected cases tested was high, with the exception of Suriname [62] and the US Virgin Islands [67] which only tested approximately 2–3% (seven reports did not give a total number of suspected cases). Most (15/25) of the identified outbreak studies in Latin America were hospital- or clinic-based. Five of the outbreak studies involved a convenience sample, and three focused on children (Haiti and Barbados in 2014, and Mexico in 2015–16) [68–70]. Almost all outbreaks were associated with the Asian lineage, except for four outbreaks in northeastern Brazil associated with the ECSA genotype; in Sergipe from 2014 to 2016 [71], Bahia in 2015 [63], Piaui in 2016 to 2017 [72] and in Bahia again in 2017 [73]. A viral lineage was not specified in 13 outbreaks. Co-circulating mosquito-borne pathogens were described in eight countries and 12 outbreaks, with DENV and ZIKV being most common.

All seroprevalence studies were associated with a reported outbreak (many of which were conducted during the outbreaks), with an IgG seropositivity range of 0–48%. Of 11 seroprevalence studies identified in the Americas, seven studies reported a proportion of inapparent infections among the identified seropositive individuals (ranging from 19–65%). Six serosurveys were clinic- or hospital-based (all used convenience samples, four focused on blood donors, and one was in children), and five were community-based.

Ongoing surveillance systems were identified in six countries in the Americas (**S5 Table**). Brazil employs an all-age, regional active surveillance system [74]. Colombia has an all-age, national, active surveillance system [75]. Jamaica has an all-age, national, active and passive surveillance system using medical practitioners, sentinel sites at primary healthcare centers, and major hospitals [76]. Suriname uses an all-age, national, active lab-based surveillance system (active since October 2014) [77]. Puerto Rico has several systems in place: an all-age, national passive physician-based surveillance system (active since the late 1960s); an all-age, national, passive and active system using forensic physician reports and post-mortem serum samples (active since 2010); and an all-age, national, active healthcare facility-based surveillance system (active since 2012) [78]. The United States has an all-age, national, passive surveillance system using local public health departments, hospitals, laboratories and healthcare providers [79].

#### Europe

Five reported outbreaks involving local transmission of chikungunya by *Ae*. *albopictus* have been reported in Europe. Two of these outbreaks occurred in Italy and three in France, all with relatively low numbers of cases (ranging from only two autochthonous cases in France in 2010, up to 699 suspected cases in Italy in 2017).

The epidemiology of chikungunya in Europe is characterized by relatively small outbreaks involving local transmission following the introduction of an imported index case. The first European outbreak was reported in northeastern Italy from June to September 2007 [80,81]. See **Table 1** for further detail on outbreak chronology. Incidence rates were only reported in Italy, and ranged from 1.2–406 suspected cases per 10,000 inhabitants [16,80] (**S3 Table**). All outbreaks in Europe reported laboratory-confirmed cases, with a relatively high proportion of clinically suspected cases tested. Four of the five outbreak studies identified involved active case-finding (three door-to-door case finding within a certain radius of the home of a confirmed case, one clinic-based surveillance study). The ECSA genotype (IOL) was identified in all European outbreaks apart from France in 2010 where no specific CHIKV lineage was identified. No co-circulating pathogens were described.

Four seroprevalence studies have been conducted in European countries (**Table 2**) with a seropositivity rate ranging from 0.4% in Turkey to 10% in Italy (**S4 Table**). One serosurvey reported a proportion of inapparent infections among the identified seropositive individuals (Italy [82]). Three serosurveys were clinic-based (two focused in blood donors, two used convenience samples), and one was community-based.

No surveillance system specific to local transmission of CHIKV was identified in Europe by this review.

## Discussion

This comprehensive review summarizes global chikungunya epidemiology between 1999 and 2020. Understanding the epidemiology of chikungunya is critical for determining the feasibility of possible chikungunya vaccine efficacy trials based on infection and disease in endemic settings and for informing the appropriate regulatory pathway for vaccines against CHIKV and post-authorization vaccine effectiveness studies. Large-scale outbreaks have been seen in Africa, Asia, and the Americas. These are also areas with co-circulating mosquito-borne pathogens, most commonly DENV. Most outbreaks in Africa, Asia, and Europe were associated with the ECSA lineage, while almost all outbreaks in Latin America were associated with the Asian lineage except for a small number of outbreaks in northeastern Brazil associated with the ECSA genotype. The Asian lineage was only identified in two Asian countries in this analysis, Indonesia and the Philippines. Specific surveillance systems for chikungunya were identified in all geographic areas (except Oceania and Europe) but relatively few were still active at the time of this systematic literature review. Globally, the highest seroprevalence was detected in the studies carried out during or directly following an outbreak, although high seroprevalence was also detected in areas where no outbreak reports were retrieved (e.g., western Kenya, Rwanda). Inapparent infections were identified in many of these seroprevalence studies. The high number of references retrieved demonstrates a global awareness of chikungunya, while the wide diversity of methodologies, study designs, and populations reflect an overall lack of standardized procedures to understand this emerging disease.

**Outbreak reports** were difficult to interpret for several reasons. First, the definition of outbreak is unclear, especially where outbreaks continue for several years or when they occur in countries with what appears to be continuous CHIKV transmission (e.g., India or Indonesia). Second, outbreak data lacked standardization overall, with most studies designed to confirm the existence of a chikungunya outbreak rather than robustly characterizing the outbreak. Most outbreak studies identified in Africa were hospital- or clinic-based, several of which involved a convenience sample with associated biases. Studies used variable testing algorithms which were not always described and relied on diverse case definitions that do not distinguish chikungunya from similar co-circulating mosquito-borne pathogens (such as DENV or ZIKV), resulting in frequent misdiagnosis and misclassifications. Third, outbreak data are incomplete. Most data are based on suspected cases, of which only a variable proportion are laboratory-tested. Finally, information on sample collection, shipment, storage, and management was often overlooked, yet important to validate testing data and further understand chikungunya epidemiology.

The spatiotemporal dynamics of chikungunya outbreaks remain unpredictable and to date have mostly affected regions with limited resources to implement appropriate and sustainable long-term surveillance. Hence, in many countries, smaller outbreaks or low-level endemic transmission are under detected due to competing priorities for pathogen surveillance. Specific surveillance systems for chikungunya were identified in only a small number of countries (**S5 Table**). We identified several active and passive surveillance systems in various Asian countries; however, most were in place for a limited time and in limited geographic areas, raising the question of long-term sustainability of the reactive surveillance measures implemented during an outbreak. India has a national surveillance system, but limited accessible information on surveillance methods, case definitions, and sample selection for laboratory testing. Furthermore, only total case numbers but no incidence or denominator data are reported, complicating interpretation.

Data retrieved in this review suggest that major outbreaks may result in seroprevalences between 30% and 70%. These data should be interpreted with caution, given the heterogeneity in study designs and methodologies of varying sensitivity applied in a wide range of geographic settings. Among the identified seroprevalence studies, the reported proportion of inapparent infections ranged from 13% to 65%, with no obvious geographic pattern detected, although other patterns might come into play. Bustos Carrillo et al. suggested that the proportion of inapparent CHIKV infections is lineage dependent and that a higher proportion of inapparent infections are associated with the Asian lineage compared to the ECSA lineage [10]. These seroprevalence data, however, support that chikungunya does not generally recur in the same region of recent high-incidence outbreaks, but in chikungunya naïve regions or perhaps regions that only suffered mild outbreaks in the recent past. In some instances, substantial seroprevalence was detected in countries or regions with no retrieved outbreak reports. This could possibly be explained by serologic cross-reactions between CHIKV and other alphaviruses (e.g., o’nyong nyong virus cited as a possibility in Rwanda [[34]), travelers contracting CHIKV in endemic regions before returning home (e.g. in Europe), lack of surveillance in these areas resulting in previous outbreaks going unreported, or high proportion of inapparent CHIKV infections.

### Gaps in epidemiological knowledge and importance for vaccine development

This review shows that, although data on chikungunya have been abundant in the past eight years, the information that can be retrieved is limited given that most studies do not fully characterize the outbreak or inform the dynamics of CHIKV infection.

The diagnostic capacity available for identifying cases of chikungunya, particularly during large outbreaks, varies among countries and regions (e.g., with higher capacity for testing and active case-finding observed in Europe). Given the similarities in clinical presentation of arboviruses, there is a clear need to implement surveillance systems with laboratory confirmation to reduce underreporting. In most countries, CHIKV co-circulates with DENV or ZIKV (among other agents), and surveillance can build on systems already in place. Further, specific case definitions are needed for all arboviruses to avoid misclassification, and lab confirmation needs to be applied more systematically. Surveillance systems tend to be only clinic or hospital-based with no community-based component, meaning that the large proportion of non-clinically attended cases (symptomatic or clinically inapparent) are missed. Understanding the extent of underreporting, for example through community-based surveillance activities, could help to address these issues and improve surveillance. Seroprevalence studies help to understand the extent of virus propagation in a given population, including inapparent infections, and therefore identify the proportion of susceptible individuals after a given outbreak. However, such studies provide insufficient information to fully understand the duration of population protection without proper participant follow-up over a sufficient period.

Vaccine efficacy trials typically require sufficient lead times to establish clinical trial infrastructure, trained personnel, regulatory and ethical approvals, and community awareness and acceptance. These preparatory activities often need to be done in areas with minimal previous clinical trial experience, but where disease incidence is more likely within a reasonable timeframe. Multiple clinical trial sites in different areas and regions may be necessary, both to increase pathogen and population diversity, and to mitigate risk of site failure or unexpectedly low incidence at individual sites. Planning for such an undertaking requires adequate knowledge of age-specific infection rates and disease incidence at these sites. For chikungunya, not only is there a paucity of such granular data, but the limited information that is available suggests that outbreaks may not recur in the same area for many years. Furthermore, current information mainly focuses on large-scale outbreaks, with limited information available on smaller outbreaks or low-level endemic transmission. Given the wide geographical distribution of chikungunya in tropical and subtropical regions and the unpredictable nature of chikungunya, the feasibility of making informed decisions about the number and location of sites for a vaccine efficacy trial will remain challenging. Also needed is a better understanding of chikungunya disease dynamics with appropriate granularity at smaller geographic scales, improvement in outbreak detection, and better insight into the duration of long-term population immunity after CHIKV circulation.

### Strengths & limitations

There were several limitations to this study. Some chikungunya outbreaks were likely missed (e.g., Kenya, Oceania outbreaks 2011–2015) [1,83,84]. The search terms may not have been exhaustive, relevant papers may have been published after June 13, 2020 (i.e., date of search), and important information may have been contained in letters to the editor, unpublished studies or sources which are not publicly available, and primary data may have not always been reported. Despite these limitations, this study has several strengths. We employed a rigorous methodology, which allowed us to screen a large number of articles for inclusion. Furthermore, we supplemented the information obtained from articles by searching relevant MoH websites to find and access surveillance reports where available. We believe the methodology employed and the sources included have allowed us to provide an informative and adequate view of epidemiological trends of chikungunya during the review period.

## Conclusions

This review confirms chikungunya as an emerging disease that has rapidly expanded to new regions in recent years. Chikungunya outbreak data present important limitations, that are likely biasing awareness of the disease to major outbreaks only, with seroprevalence data suggesting that even some major outbreaks have been missed. Global lack of specific surveillance systems and case underreporting do not allow an informed understanding of chikungunya dynamics, which appears to evolve at a granular level within countries. The establishment of CHIKV dedicated surveillance systems with laboratory confirmation and characterization of the viruses should be encouraged to bridge the data gap. Until this can be addressed, challenges affecting the feasibility of planning and conducting vaccine efficacy trials and post-authorization effectiveness studies for CHIKV vaccines will persist.

## Disclaimers

The CEPI’s authors contributed to study design, data collection and analysis, preparation of the manuscript and decision to publish.

The publication reflects only the author’s view and the European Commission is not responsible for any use that may be made of the information it contains.

The findings and conclusions in this report are those of the author(s) and do not necessarily represent the views of the Centers for Disease Control and Prevention.

## Supporting information

S1 Text Full search string. Literature search using the above search string was conducted in MEDLINE (via PubMed) on June 13, 2020.
(DOCX)

S1 PRISMA checklist Flow diagram of the literature search according to PRISMA. (DOCX)

S1 Table List of websites, data sources, and search dates. (DOCX)

S2 Table Full list of included references. (DOCX)

S3 Table Chikungunya outbreak reports, summary by country. We identified 116 references including outbreak data, as defined in the methods section. Whenever possible (overlapping dates and similar region), data were summarized per outbreak. However, in many instances it was not clear whether the reports referred to the same outbreak (for example in India), and in other cases data were kept separate because they were based on different study designs (for example in Colombia).
(XLSX)

S4 Table Overall seroprevalences reported in the different countries, and characteristics of the different studies. We identified 47 studies with seroprevalence data. Four studies, set in Tanzania, India and Iran, were excluded from the table because they reported IgM antibodies only. Of the remaining 43 studies, seroprevalence data were available for 50 different regions and times (Table 2). Most of these (45/50) reported proportions of CHIKV IgG-positive samples and 5/50 presented seroprevalence as the overall proportion of IgG and IgM antibodies. The reports were heterogeneous in population and sampling strategies: 20/50 were based on household data, 9/50 studies were based on blood donors, 4/50 were based on pregnant women, and 3/50 were based on children only. Of all 50 reports, 23 were clinic-based and 29 were carried out during or recently after an outbreak. Among the identified seroprevalence studies, 14 reported the proportion of asymptomatic participants among seropositive samples. Of these studies, 12 were household-based, with some degree of stratification in the sampling design, and 7 were carried out in the Americas. The reported proportion of asymptomatic infections ranged from 13–65%, with no obvious geographic pattern detected.
(XLSX)

S5 Table Surveillance systems in place, by country. We identified 19 examples of surveillance systems in place across 12 geographical areas from MoH websites, as outlined in the methodology.
(DOCX)
