# Supplementary material for: The global epidemiology of chikungunya from 1999 to 2020: A systematic literature review to inform the development and introduction of vaccines
Source: PLoS Negl Trop Dis. 2022 Jan 12;16(1):e0010069. doi: 10.1371/journal.pntd.0010069 (PMC8789145; doi:10.1371/journal.pntd.0010069)
Supplement: S1 Text — Literature search using the above search string was conducted in MEDLINE (via PubMed) on June 13, 2020. (DOCX) [file pntd.0010069.s001.docx]

# S1 Text: Full search string

((chikungunya[Title/Abstract]) AND ("attack rate" OR "attack rates" OR incidence OR "incidence rate" OR "incidence rates" OR prevalence OR season OR seasonality OR "risk factors" OR "risk factor" OR stratification OR age OR transmission OR "viral lineages" OR "viral lineage" OR lineages OR clades OR genotypes OR "natural infection" OR "infection-acquired immunity" OR "natural immunity" OR coinfection OR coinfections OR "concomitant infection" OR "concomitant infections" OR co-circulate OR co-circulation OR serology OR seroprevalence OR serosurvey OR serosurveys OR surveillance OR "surveillance system" OR monitoring OR representativeness OR under-reporting OR underreporting)) AND (("1999/01/01"[Date - Publication] : "3000"[Date - Publication])).

Literature search using the above search string was conducted in MEDLINE (via PubMed) on June 13, 2020.
