# Supplementary material for: The global epidemiology of chikungunya from 1999 to 2020: A systematic literature review to inform the development and introduction of vaccines
Source: PLoS Negl Trop Dis. 2022 Jan 12;16(1):e0010069. doi: 10.1371/journal.pntd.0010069 (PMC8789145; doi:10.1371/journal.pntd.0010069)
Supplement: S1 Table — (DOCX) [file pntd.0010069.s003.docx]

# S1 Table: list of websites, data sources, and search dates

| **Name** | **URL** | **Date searched** | **Notes** |
| --- | --- | --- | --- |
| WHO | <https://www.who.int/health-topics/chikungunya/#tab=tab_1> | 20-22 April 2020 | Search for "chikungunya" and retrieval of all articles with data meeting the objectives |
|  | <https://www.who.int/home/search> | 20-22 April 2020 | Search for "chikungunya" and retrieval of all articles with data meeting the objectives |
| PAHO | <https://www.paho.org/en/topics/chikungunya> | 20-22 April 2020 | Search for "chikungunya" and retrieval of all articles with data meeting the objectives |
|  | <https://www.paho.org/data/index.php/en/mnu-topics/chikv-en/550-chikv-weekly-en.html> | 03 December 2020 | Search for "chikungunya" and retrieval of all articles with data meeting the objectives |
| ECDC | <https://www.ecdc.europa.eu/en> | 20-22 April 2020 | Search for "chikungunya" and retrieval of all articles with data meeting the objectives |
| LILACS | <https://lilacs.bvsalud.org/en/> | 20-22 April 2020 | Search for "chikungunya" and retrieval of all articles with data meeting the objectives |
| Afican Index Medicus | [https://afrolib.afro.who.int/#](https://afrolib.afro.who.int/) | 12 June 2020 | Search for "chikungunya" and retrieval of all articles with data meeting the objectives |
| Scielo | <https://www.scielo.br/scielo.php?script=sci_home&lng=pt&nrm=iso> | 12 June 2020 | Search for "chikungunya" and retrieval of all articles with data meeting the objectives |
| Google Scholar | <https://scholar.google.com> | 20-22 April 2020 | Initial search for "chikungunya" review articles, and then for key references included in these reviews |
| Brazil Ministry of Health | <https://www.gov.br/saude/pt-br/assuntos/boletins-epidemiologicos-1> | 15 November 2020 | Search for "chikungunya" among epidemiological bulletins, and retrieval of articles with data meeting the objectives |
