# Supplementary material for: The global epidemiology of chikungunya from 1999 to 2020: A systematic literature review to inform the development and introduction of vaccines
Source: PLoS Negl Trop Dis. 2022 Jan 12;16(1):e0010069. doi: 10.1371/journal.pntd.0010069 (PMC8789145; doi:10.1371/journal.pntd.0010069)
Supplement: S2 Table — (DOCX) [file pntd.0010069.s004.docx]

# S2 Table: Full list of included references

| **Full references** |
| --- |
| Abhishek, K. S., and A. Chakravarti. 2019. 'Simultaneous detection of IgM antibodies against dengue and chikungunya: Coinfection or cross-reactivity?', J Family Med Prim Care, 8: 2420-23. |
| Adam, Awadalkareem, Osama ME Seidahmed, Christopher Weber, Barbara Schnierle, Jonas Schmidt-Chanasit, Sven Reiche, and Christian Jassoy. 2016. 'Low seroprevalence indicates vulnerability of eastern and central Sudan to infection with chikungunya virus', Vector-Borne and Zoonotic Diseases, 16: 290-91. |
| Adams, L. E., S. W. Martin, N. P. Lindsey, J. A. Lehman, A. Rivera, J. Kolsin, K. Landry, J. E. Staples, T. M. Sharp, G. Paz-Bailey, and M. Fischer. 2019. 'Epidemiology of Dengue, Chikungunya, and Zika Virus Disease in U.S. States and Territories, 2017', Am J Trop Med Hyg, 101: 884-90. |
| Afreen, N., F. Deeba, W. H. Khan, S. H. Haider, S. N. Kazim, R. Ishrat, I. H. Naqvi, M. Y. Shareef, S. Broor, A. Ahmed, and S. Parveen. 2014. 'Molecular characterization of dengue and chikungunya virus strains circulating in New Delhi, India', Microbiol Immunol, 58: 688-96. |
| Agarwal, A., S. Gupta, A. K. Yadav, R. K. Nema, K. Ansari, and D. Biswas. 2019. 'Molecular and phylogenetic analysis of Chikungunya virus in Central India during 2016 and 2017 outbreaks reveal high similarity with recent New Delhi and Bangladesh strains', Infect Genet Evol, 75: 103940. |
| Ahmed, S., L. Francis, R. P. Ricketts, T. Christian, K. Polson-Edwards, and B. Olowokure. 2015. 'Chikungunya virus outbreak, Dominica, 2014', Emerg Infect Dis, 21: 909-11. |
| Akinola, MT, AD El-Yuguda, DN Bukbuk, and SS Baba. 2017. 'Prevalence of IgG and IgM antibodies to Chikungunya virus among outpatients with febrile illness attending University of Maiduguri Teaching Hospital, Maiduguri, Borno State, Nigeria', African Journal of Microbiology Research, 11: 306-11. |
| Alvarado, L. I., O. D. Lorenzi, B. C. Torres-Velásquez, T. M. Sharp, L. Vargas, J. L. Muñoz-Jordán, E. A. Hunsperger, J. Pérez-Padilla, A. Rivera, G. E. González-Zeno, R. L. Galloway, M. Glass Elrod, D. L. Mathis, M. S. Oberste, W. A. Nix, E. Henderson, J. McQuiston, J. Singleton, C. Kato, C. García-Gubern, W. Santiago-Rivera, R. Muns-Sosa, J. D. Ortiz-Rivera, G. Jiménez, V. Rivera-Amill, D. A. Andújar-Pérez, K. Horiuchi, and K. M. Tomashek. 2019. 'Distinguishing patients with laboratory-confirmed chikungunya from dengue and other acute febrile illnesses, Puerto Rico, 2012-2015', PLoS Negl Trop Dis, 13: e0007562. |
| Andayi, F., R. N. Charrel, A. Kieffer, H. Richet, B. Pastorino, I. Leparc-Goffart, A. A. Ahmed, F. Carrat, A. Flahault, and X. de Lamballerie. 2014. 'A sero-epidemiological study of arboviral fevers in Djibouti, Horn of Africa', PLoS Negl Trop Dis, 8: e3299. |
| Anfasa, F., L. Provacia, C. GeurtsvanKessel, R. Wever, I. Gerstenbluth, A. D. Osterhaus, and B. E. Martina. 2017. 'Hyperferritinemia is a potential marker of chronic chikungunya: A retrospective study on the Island of Curaçao during the 2014-2015 outbreak', J Clin Virol, 86: 31-38. |
| Ang, L. W., Y. W. Kam, C. Lin, P. U. Krishnan, J. Tay, L. C. Ng, L. James, V. J. M. Lee, K. T. Goh, L. F. P. Ng, and R. T. P. Lin. 2017. 'Seroprevalence of antibodies against chikungunya virus in Singapore resident adult population', PLoS Negl Trop Dis, 11: e0006163. |
| Angelini, P., P. Macini, A. C. Finarelli, C. Pol, C. Venturelli, R. Bellini, and M. Dottori. 2008. 'Chikungunya epidemic outbreak in Emilia-Romagna (Italy) during summer 2007', Parassitologia, 50: 97-8. |
| Anish, Ts, K. Vijayakumar, and I. A. Leela. 2011. 'Domestic and Environmental Factors of Chikungunya-affected Families in Thiruvananthapuram (Rural) District of Kerala, India', J Glob Infect Dis, 3: 32-6. |
| Ansumana, R., K. H. Jacobsen, T. A. Leski, A. L. Covington, U. Bangura, M. H. Hodges, B. Lin, A. S. Bockarie, J. M. Lamin, M. J. Bockarie, and D. A. Stenger. 2013. 'Reemergence of chikungunya virus in Bo, Sierra Leone', Emerg Infect Dis, 19: 1108-10. |
| António, V. S., A. F. Muianga, J. Wieseler, S. A. Pereira, V. O. Monteiro, F. Mula, I. Chelene, I. S. Chongo, J. O. Oludele, B. M. Kümmerer, and E. S. Gudo. 2018. 'Seroepidemiology of Chikungunya Virus Among Febrile Patients in Eight Health Facilities in Central and Northern Mozambique, 2015-2016', Vector Borne Zoonotic Dis, 18: 311-16. |
| Antonio, V. S., N. A. Amade, A. F. Muianga, S. Ali, V. Monteiro, F. Mula, I. Chelene, J. Oludele, I. Chongo, A. José, O. Augusto, and E. S. Gudo. 2019. 'Retrospective investigation of antibodies against chikungunya virus (CHIKV) in serum from febrile patients in Mozambique, 2009-2015: Implications for its prevention and control', PloS one, 14: e0213941. |
| Apandi, Y., S. K. Lau, N. Izmawati, N. M. Amal, Y. Faudzi, W. Mansor, M. H. Hani, and S. Zainah. 2010. 'Identification of Chikungunya virus strains circulating in Kelantan, Malaysia in 2009', Southeast Asian J Trop Med Public Health, 41: 1374-80. |
| Appassakij, H., P. Khuntikij, M. Kemapunmanus, R. Wutthanarungsan, and K. Silpapojakul. 2013. 'Viremic profiles in asymptomatic and symptomatic chikungunya fever: a blood transfusion threat?', Transfusion, 53: 2567-74. |
| Araúz, D., L. De Urriola, J. Jones, M. Castillo, A. Martínez, E. Murillo, L. Troncoso, M. Chen, L. Abrego, B. Armién, J. M. Pascale, N. Sosa, S. López-Verges, and B. Moreno. 2016. 'Febrile or Exanthematous Illness Associated with Zika, Dengue, and Chikungunya Viruses, Panama', Emerg Infect Dis, 22: 1515-7. |
| Arif, M., P. Tauran, H. Kosasih, N. M. Pelupessy, N. Sennang, R. H. Mubin, P. Sudarmono, E. Tjitra, D. Murniati, A. Alam, M. H. Gasem, A. T. Aman, D. Lokida, U. Hadi, K. T. M. Parwati, C. Y. Lau, A. Neal, and M. Karyana. 2020. 'Chikungunya in Indonesia: Epidemiology and diagnostic challenges', PLoS Negl Trop Dis, 14: e0008355. |
| Atalay, T., S. Kaygusuz, and A. K. Azkur. 2017. 'A study of the chikungunya virus in humans in Turkey', Turk J Med Sci, 47: 1161-64. |
| Aubry, M., A. Teissier, C. Roche, V. Richard, A. S. Yan, K. Zisou, E. Rouault, V. Maria, S. Lastère, V. M. Cao-Lormeau, and D. Musso. 2015. 'Chikungunya outbreak, French Polynesia, 2014', Emerg Infect Dis, 21: 724-6. |
| Aubry, M., A. Teissier, M. Huart, S. Merceron, J. Vanhomwegen, M. Mapotoeke, T. Mariteragi-Helle, C. Roche, A. L. Vial, S. Teururai, S. Sicard, S. Paulous, P. Desprès, J. C. Manuguerra, H. P. Mallet, A. Imrie, D. Musso, X. Deparis, and V. M. Cao-Lormeau. 2018. 'Seroprevalence of Dengue and Chikungunya Virus Antibodies, French Polynesia, 2014-2015', Emerg Infect Dis, 24: 558-61. |
| Aubry, M., M. Kama, A. D. Henderson, A. Teissier, J. Vanhomwegen, T. Mariteragi-Helle, T. Paoaafaite, J. C. Manuguerra, K. Christi, C. H. Watson, C. L. Lau, A. J. Kucharski, and V. M. Cao-Lormeau. 2020. 'Low chikungunya virus seroprevalence two years after emergence in Fiji', Int J Infect Dis, 90: 223-25. |
| Auerswald, H., C. Boussioux, S. In, S. Mao, S. Ong, R. Huy, R. Leang, M. Chan, V. Duong, S. Ly, A. Tarantola, and P. Dussart. 2018. 'Broad and long-lasting immune protection against various Chikungunya genotypes demonstrated by participants in a cross-sectional study in a Cambodian rural community', Emerg Microbes Infect, 7: 13. |
| Ayorinde, A. F., A. M. Oyeyiga, N. O. Nosegbe, and O. A. Folarin. 2016. 'A survey of malaria and some arboviral infections among suspected febrile patients visiting a health centre in Simawa, Ogun State, Nigeria', J Infect Public Health, 9: 52-9. |
| Ayu, S. M., L. R. Lai, Y. F. Chan, A. Hatim, N. N. Hairi, A. Ayob, and I. C. Sam. 2010. 'Seroprevalence survey of Chikungunya virus in Bagan Panchor, Malaysia', Am J Trop Med Hyg, 83: 1245-8. |
| Azami, N. A., S. A. Salleh, S. A. Shah, H. M. Neoh, Z. Othman, S. Z. Zakaria, and R. Jamal. 2013. 'Emergence of chikungunya seropositivity in healthy Malaysian adults residing in outbreak-free locations: chikungunya seroprevalence results from the Malaysian Cohort', BMC Infect Dis, 13: 67. |
| Baba, M., C. H. Logue, B. Oderinde, H. Abdulmaleek, J. Williams, J. Lewis, T. R. Laws, R. Hewson, A. Marcello, and D' Agaro P. 2013. 'Evidence of arbovirus co-infection in suspected febrile malaria and typhoid patients in Nigeria', J Infect Dev Ctries, 7: 51-9. |
| Bacci, A., S. Marchi, N. Fievet, A. Massougbodji, R. X. Perrin, J. P. Chippaux, V. Sambri, M. P. Landini, S. Varani, and G. Rossini. 2015. 'High seroprevalence of chikungunya virus antibodies among pregnant women living in an urban area in Benin, West Africa', Am J Trop Med Hyg, 92: 1133-6. |
| Badar, Nazish, Muhammad Salman, Jamil Ansari, Uzma Aamir, Muhammad Masroor Alam, Yasir Arshad, Nighat Mushtaq, Aamer Ikram, and Javaria Qazi. 2020. 'Emergence of Chikungunya Virus, Pakistan, 2016–2017', Emerging infectious diseases, 26: 307. |
| Bagno, F. F., M. M. Figueiredo, J. Villarreal, G. C. Pereira, L. C. Godoi, and F. G. da Fonseca. 2019. 'Undetected Chikungunya virus co-infections in a Brazilian region presenting hyper-endemic circulation of Dengue and Zika', J Clin Virol, 113: 27-30. |
| Balasubramaniam, S. M., J. Krishnakumar, T. Stephen, R. Gaur, and N. Appavoo. 2011. 'Prevalence of chikungunya in urban field practice area of a private medical college, chennai', Indian J Community Med, 36: 124-7. |
| Ball, J. D., M. A. Elbadry, T. Telisma, S. K. White, S. Chavannes, M. G. Anilis, M. Prosperi, D. A. T. Cummings, J. A. Lednicky, J. G. Morris, and M. Beau de Rochars. 2019. 'Clinical and Epidemiologic Patterns of Chikungunya Virus Infection and Coincident Arboviral Disease in a School Cohort in Haiti, 2014-2015', Clin Infect Dis, 68: 919-26. |
| Ballera, J. E., M. J. Zapanta, V. C. de los Reyes, M. N. Sucaldito, and E. Tayag. 2015. 'Investigation of chikungunya fever outbreak in Laguna, Philippines, 2012', Western Pac Surveill Response J, 6: 8-11. |
| Balmaseda, A., A. Gordon, L. Gresh, S. Ojeda, S. Saborio, Y. Tellez, N. Sanchez, G. Kuan, and E. Harris. 2016. 'Clinical Attack Rate of Chikungunya in a Cohort of Nicaraguan Children', Am J Trop Med Hyg, 94: 397-9. |
| Bandyopadhyay, B., D. Bandyopadhyay, R. Bhattacharya, R. De, B. Saha, H. Mukherjee, and A. K. Hati. 2009. 'Death due to chikungunya', Trop Doct, 39: 187-8. |
| Barakat, A. M., T. Smura, S. Kuivanen, E. Huhtamo, S. Kurkela, N. Putkuri, H. J. Hasony, H. Al-Hello, and O. Vapalahti. 2016. 'The Presence and Seroprevalence of Arthropod-Borne Viruses in Nasiriyah Governorate, Southern Iraq: A Cross-Sectional Study', Am J Trop Med Hyg, 94: 794-9. |
| Barde, P. V., M. K. Shukla, P. K. Bharti, B. K. Kori, J. K. Jatav, and N. Singh. 2014. 'Co-circulation of dengue virus serotypes with chikungunya virus in Madhya Pradesh, central India', WHO South East Asia J Public Health, 3: 36-40. |
| Barr, K. L., E. Khan, J. Q. Farooqi, K. Imtiaz, D. Prakoso, F. Malik, J. A. Lednicky, and M. T. Long. 2018. 'Evidence of Chikungunya Virus Disease in Pakistan Since 2015 With Patients Demonstrating Involvement of the Central Nervous System', Front Public Health, 6: 186. |
| Bhagwati, C., M. M, K. D. Mehta, and S. G. Y. 2013. 'Profile of The Chikungunya Infection: A Neglected Vector Borne Disease which is Prevalent In The Rajkot District', J Clin Diagn Res, 7: 1008-11. |
| Bloch, D., N. M. Roth, E. V. Caraballo, J. Muñoz-Jordan, E. Hunsperger, A. Rivera, J. Pérez-Padilla, B. Rivera Garcia, and T. M. Sharp. 2016. 'Use of Household Cluster Investigations to Identify Factors Associated with Chikungunya Virus Infection and Frequency of Case Reporting in Puerto Rico', PLoS Negl Trop Dis, 10: e0005075. |
| Bonilla-Aldana, D. K., J. L. Bonilla-Aldana, J. J. García-Bustos, C. O. Lozada, and A. J. Rodríguez-Morales. 2019. 'Geographical trends of chikungunya and Zika in the Colombian Amazonian gateway department, Caqueta, 2015-2018 - Implications for public health and travel medicine', Travel Med Infect Dis: 101481. |
| MOH Brazil. Boletim epidemiológico: Monitoramento dos casos de dengue, febre de chikungunya e febre pelo vírus Zika até a Semana Epidemiológica 49, 2016. 2016. Vol.47, No.38. |
| MOH Brazil. Boletim Epidemiológico: Monitoramento dos casos de dengue, febre de chikungunya e febre pelo vírus Zika até a Semana Epidemiológica 50, 2017. 2017. Vol. 48, No.: 45. |
| MOH Brazil. Boletim epidemiológico: Monitoramento dos casos de dengue, febre de chikungunya e doença aguda pelo vírus Zika até a Semana Epidemiológica 49 de 2018. 2018. Vol. 49, No.: 59. |
| MOH Brazil. Boletim epidemiológico: Monitoramento dos casos de arboviroses urbanas transmitidas pelo Aedes (dengue, chikungunya e Zika), Semanas Epidemiológicas 01 a 52, 2019. 2020. Vol. 51, No.: 02. |
| MOH Brazil. Boletim epidemiológico: Monitoramento dos casos de arboviroses urbanas transmitidas pelo Aedes Aegypti (dengue, chikungunya e zika), semanas epidemiológicas 1 a 36, 2020. 2020. Vol. 51, No.: 38. |
| Budodo, Rule M, Pius G Horumpende, Sixbert I Mkumbaye, Blandina T Mmbaga, Richard S Mwakapuja, and Jaffu Chilongola. 2020. 'Serological evidence of exposure to Rift Valley, Dengue and Chikungunya Viruses among agropastoral communities in Manyara and Morogoro regions in Tanzania: A community Survey', BioRxiv. |
| Bustos Carrillo, F., D. Collado, N. Sanchez, S. Ojeda, B. Lopez Mercado, R. Burger-Calderon, L. Gresh, A. Gordon, A. Balmaseda, G. Kuan, and E. Harris. 2019. 'Epidemiological Evidence for Lineage-Specific Differences in the Risk of Inapparent Chikungunya Virus Infection', J Virol, 93. |
| Cabrera, Maritza, Fernando Córdova-Lepe, Nereida Valero-Cedeño, Javier Reyes-Baque, and Alfonso J Rodríguez-Morales. 2019. 'Chikungunya in Ecuador, 2014-2017: Maps and more', Travel medicine and infectious disease, 29: 63. |
| Calba, C, F Franke, C Chaud, A Decoppet, L Pigaglio, and M Auzet-Caillaud. 2018. 'Circulation autochtone de chikungunya dans deux communes du Var, août-septembre 2017', Bull Epidémiol Hebd, 24: 504-9. |
| Camacho García, Daría, Argelia Celis, Zoila Moros, Jesús Reyes Osorio, Ricardo Araujo, Andrea Alcántara, Víctor Picos Guerrero, Augusto Tarazón, Ruth Blanco, Esmeralda Vizzi, Ferdinando Liprandi, Ana I. Negredo A, María P. Sánchez-Seco, and Guillermo Comach Pérez. 2016. 'Circulación de virus Chikungunya en el estado Aragua (Venezuela) durante el año 2014', Bol. malariol. salud ambient, 56: 122-30. |
| Capeding, M. R., M. N. Chua, S. R. Hadinegoro, II Hussain, R. Nallusamy, P. Pitisuttithum, K. Rusmil, U. Thisyakorn, S. J. Thomas, N. Huu Tran, D. N. Wirawan, I. K. Yoon, A. Bouckenooghe, Y. Hutagalung, T. Laot, and T. A. Wartel. 2013. 'Dengue and other common causes of acute febrile illness in Asia: an active surveillance study in children', PLoS Negl Trop Dis, 7: e2331. |
| Carabali, M., J. K. Lim, D. C. Palencia, A. Lozano-Parra, R. M. Gelvez, K. S. Lee, J. P. Florez, V. M. Herrera, J. S. Kaufman, E. M. Rojas, and L. A. Villar. 2018. 'Burden of dengue among febrile patients at the time of chikungunya introduction in Piedecuesta, Colombia', Trop Med Int Health, 23: 1231-41. |
| Caraballo Rodelo, Arístides, Ignacio Chica Arrieta, and Monica Ocampo Rivero. 2017. 'Epidemiological behavior of Dengue, Chikungunya and Zika. Córdoba-Colombia. 2014-2017'. |
| Cardoso, C. W., M. Kikuti, A. P. Prates, I. A. Paploski, L. B. Tauro, M. M. Silva, P. Santana, M. F. Rego, M. G. Reis, U. Kitron, and G. S. Ribeiro. 2017. 'Unrecognized Emergence of Chikungunya Virus during a Zika Virus Outbreak in Salvador, Brazil', PLoS Negl Trop Dis, 11: e0005334. |
| Cardoso, F. D., I. M. Rezende, E. L. T. Barros, L. Sacchetto, Tccs Garcês, N. I. O. Silva, P. A. Alves, J. O. Soares, E. G. Kroon, Actdc Pereira, B. P. Drumond, and G. P. Ferreira. 2019. 'Circulation of Chikungunya virus East-Central-South Africa genotype during an outbreak in 2016-17 in Piaui State, Northeast Brazil', Rev Inst Med Trop Sao Paulo, 61: e57. |
| Caron, M., C. Paupy, G. Grard, P. Becquart, I. Mombo, B. B. Nso, F. Kassa Kassa, D. Nkoghe, and E. M. Leroy. 2012. 'Recent introduction and rapid dissemination of Chikungunya virus and Dengue virus serotype 2 associated with human and mosquito coinfections in Gabon, central Africa', Clin Infect Dis, 55: e45-53. |
| Carrera, J. P., Y. Díaz, B. Denis, I. Barahona de Mosca, D. Rodriguez, I. Cedeño, D. Arauz, P. González, L. Cerezo, L. Moreno, L. García, L. E. Sáenz, M. A. Atencio, E. Rojas-Fermin, F. Vizcaino, N. Perez, B. Moreno, S. López-Vergès, A. Valderrama, and B. Armién. 2017. 'Unusual pattern of chikungunya virus epidemic in the Americas, the Panamanian experience', PLoS Negl Trop Dis, 11: e0005338. |
| Carrillo-Hernández, M. Y., J. Ruiz-Saenz, L. J. Villamizar, S. Y. Gómez-Rangel, and M. Martínez-Gutierrez. 2018. 'Co-circulation and simultaneous co-infection of dengue, chikungunya, and zika viruses in patients with febrile syndrome at the Colombian-Venezuelan border', BMC Infect Dis, 18: 61. |
| Carvalho, F. R., T. Medeiros, R. A. O. Vianna, G. Douglass-Jaimes, P. C. G. Nunes, M. D. S. Quintans, C. F. Souza, S. M. B. Cavalcanti, F. B. Dos Santos, S. A. Oliveira, C. A. A. Cardoso, and A. A. Silva. 2019. 'Simultaneous circulation of arboviruses and other congenital infections in pregnant women in Rio de Janeiro, Brazil', Acta Trop, 192: 49-54. |
| Cassadou, S., S. Boucau, M. Petit-Sinturel, P. Huc, I. Leparc-Goffart, and M. Ledrans. 2014. 'Emergence of chikungunya fever on the French side of Saint Martin island, October to December 2013', Euro Surveill, 19. |
| CDC. 2012. 'Chikungunya outbreak--Cambodia, February-March 2012', MMWR Morb Mortal Wkly Rep, 61: 737-40. |
| Chahar, H. S., P. Bharaj, L. Dar, R. Guleria, S. K. Kabra, and S. Broor. 2009. 'Co-infections with chikungunya virus and dengue virus in Delhi, India', Emerg Infect Dis, 15: 1077-80. |
| Chakravarti, A., S. Malik, S. Tiwari, and A. Ashraf. 2011. 'A study of Chikungunya outbreak in Delhi', J Commun Dis, 43: 259-63. |
| Charlys da Costa, A., J. Thézé, S. C. V. Komninakis, R. L. Sanz-Duro, M. R. L. Felinto, L. C. C. Moura, I. M. O. Barroso, L. E. C. Santos, M. A. L. Nunes, A. A. Moura, J. Lourenço, X. Deng, E. L. Delwart, Mrdas Guimarães, O. G. Pybus, E. C. Sabino, and N. R. Faria. 2017. 'Spread of Chikungunya Virus East/Central/South African Genotype in Northeast Brazil', Emerg Infect Dis, 23: 1742-44. |
| Chattopadhyay, S., R. Mukherjee, A. Nandi, and N. Bhattacharya. 2016. 'Chikungunya virus infection in West Bengal, India', Indian J Med Microbiol, 34: 213-5. |
| Chipwaza, B., J. P. Mugasa, M. Selemani, M. Amuri, F. Mosha, S. D. Ngatunga, and P. S. Gwakisa. 2014. 'Dengue and Chikungunya fever among viral diseases in outpatient febrile children in Kilosa district hospital, Tanzania', PLoS Negl Trop Dis, 8: e3335. |
| Chopra, A., V. Anuradha, R. Ghorpade, and M. Saluja. 2012. 'Acute Chikungunya and persistent musculoskeletal pain following the 2006 Indian epidemic: a 2-year prospective rural community study', Epidemiol Infect, 140: 842-50. |
| Chou, Y. C., C. J. Hsieh, C. A. Cheng, D. C. Wu, W. C. Wu, F. H. Lin, and C. P. Yu. 2020. 'Epidemiologic Characteristics of Imported and Domestic Chikungunya Cases in Taiwan: A 13-Year Retrospective Study', Int J Environ Res Public Health, 17. |
| Chua, K. B. 2010. 'Epidemiology of chikungunya in Malaysia: 2006-2009', Med J Malaysia, 65: 277-82. |
| Clements, T. L., C. A. Rossi, A. K. Irish, H. Kibuuka, L. A. Eller, M. L. Robb, P. Kataaha, N. L. Michael, L. E. Hensley, and R. J. Schoepp. 2019. 'Chikungunya and O'nyong-nyong Viruses in Uganda: Implications for Diagnostics', Open Forum Infect Dis, 6: ofz001. |
| Cordel, H. 2006. 'Chikungunya outbreak on Reunion: update', Euro Surveill, 11: E060302.3. |
| Cortes-Escamilla, A., H. López-Gatell, MÁ Sánchez-Alemán, J. Hegewisch-Taylor, M. Hernández-Ávila, and C. M. Alpuche-Aranda. 2018. 'The hidden burden of Chikungunya in central Mexico: results of a small-scale serosurvey', Salud Publica Mex, 60: 63-70. |
| Costa, Sdsb, Mdrfc Branco, J. Aquino Junior, Z. M. R. Rodrigues, R. C. S. Queiroz, A. S. Araujo, A. P. B. Câmara, P. S. D. Santos, E. D. A. Pereira, Mdsd Silva, Frvd Costa, Avdd Santos, M. N. L. Medeiros, J. O. Alcântara Júnior, V. V. Vasconcelos, A. M. D. Santos, and Aamd Silva. 2018. 'Spatial analysis of probable cases of dengue fever, chikungunya fever and zika virus infections in Maranhao State, Brazil', Rev Inst Med Trop Sao Paulo, 60: e62. |
| Crump, J. A., A. B. Morrissey, W. L. Nicholson, R. F. Massung, R. A. Stoddard, R. L. Galloway, E. E. Ooi, V. P. Maro, W. Saganda, G. D. Kinabo, C. Muiruri, and J. A. Bartlett. 2013. 'Etiology of severe non-malaria febrile illness in Northern Tanzania: a prospective cohort study', PLoS Negl Trop Dis, 7: e2324. |
| Cunha, M. D. P., C. A. D. Santos, D. F. L. Neto, A. S. Schanoski, S. Z. Pour, S. D. Passos, M. S. F. Souza, D. D. Costa, and P. M. A. Zanotto. 2017. 'Outbreak of chikungunya virus in a vulnerable population of Sergipe, Brazil-A molecular and serological survey', J Clin Virol, 97: 44-49. |
| Cunha, R. V., K. S. Trinta, C. A. Montalbano, M. V. Sucupira, M. M. de Lima, E. Marques, I. H. Romanholi, and J. Croda. 2017. 'Seroprevalence of Chikungunya Virus in a Rural Community in Brazil', PLoS Negl Trop Dis, 11: e0005319. |
| Dalvi, A. P. R., and J. U. Braga. 2019. 'Spatial diffusion of the 2015-2016 Zika, dengue and chikungunya epidemics in Rio de Janeiro Municipality, Brazil', Epidemiol Infect, 147: e237. |
| Darcy, A. W., S. Kanda, T. Dalipanda, C. Joshua, T. Shimono, P. Lamaningao, N. Mishima, and T. Nishiyama. 2020. 'Multiple arboviral infections during a DENV-2 outbreak in Solomon Islands', Trop Med Health, 48: 33. |
| Dariano, D. F., C. R. Taitt, K. H. Jacobsen, U. Bangura, A. S. Bockarie, M. J. Bockarie, J. Lahai, J. M. Lamin, T. A. Leski, C. Yasuda, D. A. Stenger, and R. Ansumana. 2017. 'Surveillance of Vector-Borne Infections (Chikungunya, Dengue, and Malaria) in Bo, Sierra Leone, 2012-2013', Am J Trop Med Hyg, 97: 1151-54. |
| Das, B., A. Sahu, M. Das, A. Patra, B. Dwibedi, S. K. Kar, and R. K. Hazra. 2012. 'Molecular investigations of chikungunya virus during outbreaks in Orissa, Eastern India in 2010', Infect Genet Evol, 12: 1094-101. |
| Dash, P. K., M. M. Parida, S. R. Santhosh, S. K. Verma, N. K. Tripathi, S. Ambuj, P. Saxena, N. Gupta, M. Chaudhary, J. P. Babu, V. Lakshmi, N. Mamidi, M. V. Subhalaxmi, P. V. Lakshmana Rao, and K. Sekhar. 2007. 'East Central South African genotype as the causative agent in reemergence of Chikungunya outbreak in India', Vector Borne Zoonotic Dis, 7: 519-27. |
| de Souza, T. M. A., E. D. Ribeiro, V. C. E. Corrêa, P. V. Damasco, C. C. Santos, F. de Bruycker-Nogueira, T. Chouin-Carneiro, Nrdc Faria, P. C. G. Nunes, M. Heringer, Mdrq Lima, J. Badolato-Corrêa, M. D. C. Cipitelli, E. L. Azeredo, R. M. R. Nogueira, and F. B. Dos Santos. 2018. 'Following in the Footsteps of the Chikungunya Virus in Brazil: The First Autochthonous Cases in Amapá in 2014 and Its Emergence in Rio de Janeiro during 2016', Viruses, 10. |
| de Souza Costa, M. C., L. M. Siqueira Maia, V. Costa de Souza, A. M. Gonzaga, V. Correa de Azevedo, L. Ramos Martins, J. H. Chavez Pavoni, F. Gomes Naveca, and R. Dezengrini Slhessarenko. 2019. 'Arbovirus investigation in patients from Mato Grosso during Zika and Chikungunya virus introdution in Brazil, 2015-2016', Acta Trop, 190: 395-402. |
| Delisle, E., C. Rousseau, B. Broche, I. Leparc-Goffart, G. L'Ambert, A. Cochet, C. Prat, V. Foulongne, J. B. Ferre, O. Catelinois, O. Flusin, E. Tchernonog, I. E. Moussion, A. Wiegandt, A. Septfons, A. Mendy, M. B. Moyano, L. Laporte, J. Maurel, F. Jourdain, J. Reynes, M. C. Paty, and F. Golliot. 2015. 'Chikungunya outbreak in Montpellier, France, September to October 2014', Euro Surveill, 20. |
| Dellagi, K., N. Salez, M. Maquart, S. Larrieu, A. Yssouf, R. Silaï, I. Leparc-Goffart, P. Tortosa, and X. de Lamballerie. 2016. 'Serological Evidence of Contrasted Exposure to Arboviral Infections between Islands of the Union of Comoros (Indian Ocean)', PLoS Negl Trop Dis, 10: e0004840. |
| Demanou, M., C. Antonio-Nkondjio, E. Ngapana, D. Rousset, C. Paupy, J. C. Manuguerra, and H. Zeller. 2010. 'Chikungunya outbreak in a rural area of Western Cameroon in 2006: A retrospective serological and entomological survey', BMC Res Notes, 3: 128. |
| Deshpande, G. R., B. Tilekar, S. L. Hundekar, K. Khutwad, R. S. Gunjikar, S. Vidhate, and G. N. Sapkal. 2018. 'Molecular characterization of chikungunya virus from urbanized villages of Pune, Maharashtra, India, 2016', Indian J Med Res, 148: 756-58. |
| Desjardins, MR, A Whiteman, I Casas, and E Delmelle. 2018. 'Space-time clusters and co-occurrence of chikungunya and dengue fever in Colombia from 2015 to 2016', Acta tropica, 185: 77-85. |
| Dias, J. P., Mdcn Costa, G. S. Campos, E. S. Paixão, M. S. Natividade, F. R. Barreto, M. S. C. Itaparica, C. Goes, F. L. S. Oliveira, E. B. Santana, N. S. J. Silva, C. A. A. Brito, L. C. Rodrigues, S. I. Sardi, R. C. Saavedra, and M. G. Teixeira. 2018. 'Seroprevalence of Chikungunya Virus after Its Emergence in Brazil', Emerg Infect Dis, 24: 617-24. |
| Dinkar, A., J. Singh, P. Prakash, A. Das, and G. Nath. 2018. 'Hidden burden of chikungunya in North India. J Infect Public Health. Jul-Aug 2018;11(4):586-591. doi: 10.1016/j.jiph.2017.09.008. Epub 2017 Oct 6 |
| Ditsuwan, T., T. Liabsuetrakul, V. Chongsuvivatwong, S. Thammapalo, and E. McNeil. 2011. 'Assessing the spreading patterns of dengue infection and chikungunya fever outbreaks in lower southern Thailand using a geographic information system', Ann Epidemiol, 21: 253-61. |
| Dodero-Rojas, E., L. G. Ferreira, V. B. P. Leite, J. N. Onuchic, and V. G. Contessoto. 2020. 'Modeling Chikungunya control strategies and Mayaro potential outbreak in the city of Rio de Janeiro', PloS one, 15: e0222900. |
| Doke, P. P., D. S. Dakhure, and A. V. Patil. 2011. 'A clinico-epidemiological study of Chikungunya outbreak in Maharashtra State, India', Indian J Public Health, 55: 313-6. |
| Dominguez, M, and A Economopoulou. 2005. 'Surveillance active des formes émergentes hospitalières de chikungunya', La Réunion, avril. |
| Dorléans, Frédérique, Bruno Hoen, Fatiha Najioullah, Cecile Herrmann-Storck, Kinda Maria Schepers, Sylvie Abel, Isabelle Lamaury, Laurence Fagour, Raymond Cesaire, and Stéphanie Guyomard. 2018. 'Outbreak of chikungunya in the French Caribbean islands of Martinique and Guadeloupe: findings from a hospital-based surveillance system (2013–2015)', The American journal of tropical medicine and hygiene, 98: 1819-25. |
| Dos Santos, S. Marinho R., R. L. Sanz Duro, G. L. Santos, J. Hunter, M. da Aparecida Rodrigues Teles, R. Brustulin, F. A. de Padua Milagres, E. C. Sabino, R. S. Diaz, and S. V. Komninakis. 2020. 'Detection of coinfection with Chikungunya virus and Dengue virus serotype 2 in serum samples of patients in State of Tocantins, Brazil', J Infect Public Health, 13: 724-29. |
| Dourado, Cynthia Angélica Ramos Oliveira, Evelyn Maria Braga Quirino, Clarissa Mourão Pinho, Mônica Alice Santos da Silva, Slayne Rayane Gomes de Souza, and Maria Sandra Andrade. 2019. 'Aspectos clínicos e epidemiológicos dos idosos com febre de Chikungunya', Rev Rene (Online), 20: e41184-e84. |
| Duncan, Jacqueline, Kelly Ann Gordon-Johnson, Marshall K.Tulloch-Reid, Colette Cunningham-Myrie, Kacey Ernst, Nathlee McMorris, Andriene Grant, Marcia Graham, Daisylyn Chin, and Karen Webster-Kerr. 2017. 'Chikungunya: important lessons from the Jamaican experience', Revista Panamericana de Salud Pública, 41: e60. |
| Duong, V., A. C. Andries, C. Ngan, T. Sok, B. Richner, N. Asgari-Jirhandeh, S. Bjorge, R. Huy, S. Ly, D. Laurent, B. Hok, M. C. Roces, S. Ong, M. C. Char, V. Deubel, A. Tarantola, and P. Buchy. 2012. 'Reemergence of Chikungunya virus in Cambodia', Emerg Infect Dis, 18: 2066-9. |
| Dutta, P., S. A. Khan, A. M. Khan, J. Borah, P. Chowdhury, and J. Mahanta. 2011. 'First evidence of chikungunya virus infection in Assam, Northeast India', Trans R Soc Trop Med Hyg, 105: 355-7. |
| Dutta, P., S. A. Khan, N. K. Hazarika, and S. Chetry. 2017. 'Molecular and phylogenetic evidence of chikungunya virus circulating in Assam, India', Indian J Med Microbiol, 35: 389-93. |
| Dwibedi, B., J. Sabat, N. Mahapatra, S. K. Kar, A. S. Kerketta, R. K. Hazra, S. K. Parida, N. S. Marai, and M. K. Beuria. 2011. 'Rapid spread of chikungunya virus infection in Orissa: India', Indian J Med Res, 133: 316-21. |
| ECDC. 2007. MISSION REPORT CHIKUNGUNYA IN ITALY (https://www.ecdc.europa.eu/en/publications-data/mission-report-chikungunya-italy). |
| ECDC, 2019. communicable-disease-threats-report-26-january-2019 (https://www.ecdc.europa.eu/en/publications-data?s=chikungunya&sort_by=search_api_relevance&sort_order=DESC&page=4). |
| Edwards, T., L. D. Signor, C. Williams, E. Donis, L. E. Cuevas, and E. R. Adams. 2016. 'Co-infections with Chikungunya and Dengue Viruses, Guatemala, 2015', Emerg Infect Dis, 22: 2003-05. |
| Elenga, N., M. Folin, Y. M. Vandamme, E. Cuadro-Alvarez, L. Long, F. Njuieyon, E. Martin, R. Kom-Tchameni, A. Defo, S. Herinantenaina Razafindrakoto, Y. Mrsic, P. Couppie, M. Nacher, and J. Dufour. 2017. 'Chikungunya Infection in Hospitalized Febrile Infants Younger Than 3 Months of Age', Pediatr Infect Dis J, 36: 736-40. |
| Elenjickal, M. G., and S. Sushamabai. 2009. 'Outbreak of Chikungunya disease in Kerala in 2007', Indian Pediatr, 46: 440-1. |
| Faruque, L. I., R. U. Zaman, E. S. Gurley, R. F. Massung, A. S. Alamgir, R. L. Galloway, A. M. Powers, Y. Bai, M. Kosoy, W. L. Nicholson, M. Rahman, and S. P. Luby. 2017. 'Prevalence and clinical presentation of Rickettsia, Coxiella, Leptospira, Bartonella and chikungunya virus infections among hospital-based febrile patients from December 2008 to November 2009 in Bangladesh', BMC Infect Dis, 17: 141. |
| Faustine, Novati L, Elias J Sabuni, Arnold J Ndaro, Eliakimu Paul, and Jaffu O Chilongola. 2017. 'Chikungunya, Dengue and West Nile virus Infections in Northern Tanzania', Journal of Advances in Medicine and Medical Research: 1-7. |
| Feldstein, L. R., E. M. Ellis, A. Rowhani-Rahbar, M. E. Halloran, and B. R. Ellis. 2016. 'The First Reported Outbreak of Chikungunya in the U.S. Virgin Islands, 2014-2015', Am J Trop Med Hyg, 95: 885-89. |
| Fokam, E. B., L. D. Levai, H. Guzman, P. A. Amelia, V. P. Titanji, R. B. Tesh, and S. C. Weaver. 2010. 'Silent circulation of arboviruses in Cameroon', East Afr Med J, 87: 262-8. |
| Franke F, Giron S, Cochet A, Jeannin C, Leparc-Goffart I, de Valk H, et al. 2019. 'Émergences de dengue et de chikungunya en France métropolitaine, 2010-2018', Bull Epidémiol Hebd., 19: 374-82. |
| Freitas, L. P., O. G. Cruz, R. Lowe, and Sá Carvalho M. 2019. 'Space-time dynamics of a triple epidemic: dengue, chikungunya and Zika clusters in the city of Rio de Janeiro', Proc Biol Sci, 286: 20191867. |
| Fritz, M., R. Taty Taty, C. Portella, C. Guimbi, M. Mankou, E. M. Leroy, and P. Becquart. 2019. 'Re-emergence of chikungunya in the Republic of the Congo in 2019 associated with a possible vector-host switch', Int J Infect Dis, 84: 99-101. |
| Fuller, T. L., G. Calvet, C. Genaro Estevam, J. Rafael Angelo, G. J. Abiodun, U. A. Halai, B. De Santis, P. Carvalho Sequeira, E. Machado Araujo, S. Alves Sampaio, M. C. Lima de Mendonça, A. Fabri, R. M. Ribeiro, R. Harrigan, T. B. Smith, C. Raja Gabaglia, P. Brasil, A. M. Bispo de Filippis, and K. Nielsen-Saines. 2017. 'Behavioral, climatic, and environmental risk factors for Zika and Chikungunya virus infections in Rio de Janeiro, Brazil, 2015-16', PloS one, 12: e0188002. |
| Galatas, B., S. Ly, V. Duong, K. Baisley, K. Nguon, S. Chan, R. Huy, S. Ly, S. Sorn, L. Som, P. Buchy, and A. Tarantola. 2016. 'Long-Lasting Immune Protection and Other Epidemiological Findings after Chikungunya Emergence in a Cambodian Rural Community, April 2012', PLoS Negl Trop Dis, 10: e0004281. |
| Galate, L. B., S. R. Agrawal, J. S. Shastri, and V. Londhey. 2016. 'Chikungunya Fever Among Patients with Acute Febrile Illness Attending a Tertiary Care Hospital in Mumbai', J Lab Physicians, 8: 85-9. |
| Gallian, P., I. Leparc-Goffart, P. Richard, F. Maire, O. Flusin, R. Djoudi, J. Chiaroni, R. Charrel, P. Tiberghien, and X. de Lamballerie. 2017. 'Epidemiology of Chikungunya Virus Outbreaks in Guadeloupe and Martinique, 2014: An Observational Study in Volunteer Blood Donors', PLoS Negl Trop Dis, 11: e0005254. |
| Garay-Morán, Carolina, Juan Francisco Román-Pedroza, Irma López-Martínez, José Cruz Rodríguez-Martínez, Cuitláhuac Ruiz-Matus, Pablo Kuri-Morales, and José Alberto Díaz-Quiñonez. 2017. 'Caracterización clínica y epidemiológica de fiebre chikungunya en México', Revista Panamericana de Salud Pública, 41. |
| García López, Guillermo Alfredo. 2015. 'Caracterización clínica epidemiológica de la chikungunya en la población atendida por El instituto salvadoreño del seguro social de junio a noviembre. El Salvador 2014': 44-44. |
| Gasem, M. H., H. Kosasih, E. Tjitra, B. Alisjahbana, M. Karyana, D. Lokida, A. Neal, C. J. Liang, A. T. Aman, M. Arif, P. Sudarmono, Suharto, T. P. Merati, V. Lisdawati, Siswanto, S. Siddiqui, and H. C. Lane. 2020. 'An observational prospective cohort study of the epidemiology of hospitalized patients with acute febrile illness in Indonesia', PLoS Negl Trop Dis, 14: e0007927. |
| Gay, N., D. Rousset, P. Huc, S. Matheus, M. Ledrans, J. Rosine, S. Cassadou, and H. Noël. 2016. 'Seroprevalence of Asian Lineage Chikungunya Virus Infection on Saint Martin Island, 7 Months After the 2013 Emergence', Am J Trop Med Hyg, 94: 393-96. |
| Gérardin, P., V. Guernier, J. Perrau, A. Fianu, K. Le Roux, P. Grivard, A. Michault, X. de Lamballerie, A. Flahault, and F. Favier. 2008. 'Estimating Chikungunya prevalence in La Réunion Island outbreak by serosurveys: two methods for two critical times of the epidemic', BMC Infect Dis, 8: 99. |
| Gerardin, Patrick, Joëlle Perrau, Adrian Fianu, and François Favier. 2008. 'Déterminants de l’infection à virus chikungunya à La Réunion: résultats de l’enquête Serochik de séroprévalence en population, août-octobre 2006', Bulletin épidémiologique hebdomadaire: 40. |
| Goeijenbier, M., G. Aron, F. Anfasa, Å Lundkvist, J. Verner-Carlsson, C. B. Reusken, B. E. Martina, E. C. van Gorp, and L. Resida. 2015. 'Emerging Viruses in the Republic of Suriname: Retrospective and Prospective Study into Chikungunya Circulation and Suspicion of Human Hantavirus Infections, 2008-2012 and 2014', Vector Borne Zoonotic Dis, 15: 611-8. |
| Gordon, A., L. Gresh, S. Ojeda, G. Chowell, K. Gonzalez, N. Sanchez, S. Saborio, J. C. Mercado, G. Kuan, A. Balmaseda, and E. Harris. 2018. 'Differences in Transmission and Disease Severity Between 2 Successive Waves of Chikungunya', Clin Infect Dis, 67: 1760-67. |
| Gregianini, T. S., T. Ranieri, C. Favreto, Z. M. A. Nunes, G. L. Tumioto Giannini, N. D. Sanberg, M. T. M. da Rosa, and A. B. G. da Veiga. 2017. 'Emerging arboviruses in Rio Grande do Sul, Brazil: Chikungunya and Zika outbreaks, 2014-2016', Rev Med Virol, 27. |
| Grossi-Soyster, E. N., E. A. J. Cook, W. A. de Glanville, L. F. Thomas, A. R. Krystosik, J. Lee, C. N. Wamae, S. Kariuki, E. M. Fèvre, and A. D. LaBeaud. 2017. 'Serological and spatial analysis of alphavirus and flavivirus prevalence and risk factors in a rural community in western Kenya', PLoS Negl Trop Dis, 11: e0005998. |
| Gudo, E. S., G. Pinto, S. Vene, A. Mandlaze, A. F. Muianga, J. Cliff, and K. Falk. 2015. 'Serological Evidence of Chikungunya Virus among Acute Febrile Patients in Southern Mozambique', PLoS Negl Trop Dis, 9: e0004146. |
| Guhar, D., N. Jamil, S. Jahan Talpur, G. Ahmed Channa, M. Wajeeh, M. Zohaib Khan, and S. Khan. 2018. 'The 2016-2017 Chikungunya Outbreak in Karachi', PLoS Curr, 10. |
| Haque F, Rahman M, Banu NN, et al. An epidemic of chikungunya in northwestern Bangladesh in 2011. PLoS One. 2019;14(3):e0212218. Published 2019 Mar 11. doi:10.1371/journal.pone.0212218 |
| Hennessey, Morgan J, Esther M Ellis, Mark J Delorey, Amanda J Panella, Olga I Kosoy, Hannah L Kirking, Grace D Appiah, Jin Qin, Alison J Basile, and Leora R Feldstein. 2018. 'Seroprevalence and symptomatic attack rate of chikungunya virus infection, United States Virgin Islands, 2014–2015', The American journal of tropical medicine and hygiene, 99: 1321-26. |
| Henry, Maria, Lorraine Francis, Virginia Asin, Karen Polson-Edwards, and Babatunde Olowokure. 2017. 'Chikungunya virus outbreak in Sint Maarten, 2013–2014', Revista Panamericana de Salud Pública, 41: e61. |
| Hertz, Julian T, O Michael Munishi, Eng Eong Ooi, Shiqin Howe, Wen Yan Lim, Angelia Chow, Anne B Morrissey, John A Bartlett, Jecinta J Onyango, and Venance P Maro. 2012. 'Chikungunya and dengue fever among hospitalized febrile patients in northern Tanzania', The American journal of tropical medicine and hygiene, 86: 171-77. |
| Hisamuddin, M., A. Tazeen, M. Abdullah, M. Islamuddin, N. Parveen, A. Islam, M. I. Faizan, A. Hamza, I. H. Naqvi, H. N. Verma, A. Malik, A. Ahmed, and S. Parveen. 2018. 'Co-circulation of Chikungunya and Dengue viruses in Dengue endemic region of New Delhi, India during 2016', Epidemiol Infect, 146: 1642-53. |
| Ho, K., L. W. Ang, B. H. Tan, C. S. Tang, P. L. Ooi, L. James, and G. Kee Tai. 2011. 'Epidemiology and control of chikungunya fever in Singapore', J Infect, 62: 263-70. |
| Horwood, P. F., L. J. Reimer, R. Dagina, M. Susapu, G. Bande, M. Katusele, G. Koimbu, S. Jimmy, B. Ropa, P. M. Siba, and B. I. Pavlin. 2013. 'Outbreak of chikungunya virus infection, Vanimo, Papua New Guinea', Emerg Infect Dis, 19: 1535-8. |
| Hsu, C. H., F. Cruz-Lopez, D. Vargas Torres, J. Perez-Padilla, O. D. Lorenzi, A. Rivera, J. E. Staples, E. Lugo, J. Munoz-Jordan, M. Fischer, C. Garcia Gubern, B. Rivera Garcia, L. Alvarado, and T. M. Sharp. 2019. 'Risk factors for hospitalization of patients with chikungunya virus infection at sentinel hospitals in Puerto Rico', PLoS Negl Trop Dis, 13: e0007084. |
| Humphrey, J. M., E. S. Al-Absi, M. M. Hamdan, S. S. Okasha, D. M. Al-Trmanini, H. G. El-Dous, S. R. Dargham, J. Schieffelin, L. J. Abu-Raddad, and G. K. Nasrallah. 2019. 'Dengue and chikungunya seroprevalence among Qatari nationals and immigrants residing in Qatar', PloS one, 14: e0211574. |
| MOH India. 2020. Chikungunya situation in India (https://nvbdcp.gov.in/index4.php?lang=1&level=0&linkid=486&lid=3765). |
| Inziani, Mary, Ferdinard Adungo, Janet Awando, Richelle Kihoro, Shingo Inoue, Kouichi Morita, Elizabeth Obimbo, Francis Onyango, and Matilu Mwau. 2020. 'Seroprevalence of yellow fever, dengue, West Nile and chikungunya viruses in children in Teso South Sub-County, Western Kenya', International Journal of Infectious Diseases, 91: 104-10. |
| Jain, Jaspreet, Kaustuv Nayak, Neha Tanwar, Rajni Gaind, Bhupendra Gupta, JS Shastri, Raj K Bhatnagar, Murali Krishna Kaja, Anmol Chandele, and Sujatha Sunil. 2017. 'Clinical, serological, and virological analysis of 572 chikungunya patients from 2010 to 2013 in India', Clinical infectious diseases, 65: 133-40. |
| Kabir, I., M. Dhimal, R. Müller, S. Banik, and U. Haque. 2017. 'The 2017 Dhaka chikungunya outbreak', Lancet Infect Dis, 17: 1118. |
| Kajeguka, Debora C, Robert D Kaaya, Steven Mwakalinga, Rogathe Ndossi, Arnold Ndaro, Jaffu O Chilongola, Franklin W Mosha, Karin L Schiøler, Reginald A Kavishe, and Michael Alifrangis. 2016. 'Prevalence of dengue and chikungunya virus infections in north-eastern Tanzania: a cross sectional study among participants presenting with malaria-like symptoms', BMC infectious diseases, 16: 183. |
| Kajeguka, Debora C, Robert D Kaaya, Rachelle Desrochers, Mahmood Iranpour, Reginald A Kavishe, Steven Mwakalinga, Karin L Schiøler, Michael Alifrangis, Robbin Lindsay, and Antonia Dibernardo. 2017. 'Mapping clusters of chikungunya and dengue transmission in northern Tanzania using disease exposure and vector data', Tanzania Journal of Health Research, 19. |
| Kajeguka, Debora C, Maulid Msonga, Karin L Schiøler, Dan W Meyrowitsch, Polyxeni Syrianou, Filemoni Tenu, Michael Alifrangis, Franklin W Mosha, and Reginald A Kavishe. 2017. 'Individual and environmental risk factors for dengue and chikungunya seropositivity in North-Eastern Tanzania', Infection, disease & health, 22: 65-76. |
| Kannan, M., R. Rajendran, I. P. Sunish, R. Balasubramaniam, N. Arunachalam, R. Paramsivan, S. C. Tewari, P. P. Samuel, and B. K. Tyagi. 2009. 'A study on chikungunya outbreak during 2007 in Kerala, south India', Indian J Med Res, 129: 311-5. |
| Kaur, P., M. Ponniah, M. V. Murhekar, V. Ramachandran, R. Ramachandran, H. K. Raju, V. Perumal, A. C. Mishra, and M. D. Gupte. 2008. 'Chikungunya outbreak, South India, 2006', Emerg Infect Dis, 14: 1623-5. |
| Kaur, N., J. Jain, A. Kumar, M. Narang, M. K. Zakaria, A. Marcello, D. Kumar, R. Gaind, and S. Sunil. 2017. 'Chikungunya outbreak in Delhi, India, 2016: report on coinfection status and comorbid conditions in patients', New Microbes New Infect, 20: 39-42. |
| Kaur, M., K. Singh, S. K. Sidhu, P. Devi, M. Kaur, S. Soneja, and N. Singh. 2018. 'Coinfection of chikungunya and dengue viruses: A serological study from North Western region of Punjab, India', J Lab Physicians, 10: 443-47. |
| Kautz, T. F., E. E. Díaz-González, J. H. Erasmus, I. R. Malo-García, R. M. Langsjoen, E. I. Patterson, D. I. Auguste, N. L. Forrester, R. M. Sanchez-Casas, M. Hernández-Ávila, C. M. Alpuche-Aranda, S. C. Weaver, and I. Fernández-Salas. 2015. 'Chikungunya Virus as Cause of Febrile Illness Outbreak, Chiapas, Mexico, 2014', Emerg Infect Dis, 21: 2070-3. |
| Kawle, A. P., A. R. Nayak, S. S. Bhullar, S. R. Borkar, S. D. Patankar, H. F. Daginawala, L. R. Singh, and R. S. Kashyap. 2017. 'Seroprevalence and clinical manifestations of chikungunya virus infection in rural areas of Chandrapur, Maharashtra, India', J Vector Borne Dis, 54: 35-43. |
| Kendrick, K., D. Stanek, and C. Blackmore. 2014. 'Notes from the field: Transmission of chikungunya virus in the continental United States--Florida, 2014', MMWR Morb Mortal Wkly Rep, 63: 1137. |
| Khan, S. A., P. Dutta, R. Topno, J. Borah, P. Chowdhury, and J. Mahanta. 2015. 'Chikungunya outbreak in Garo Hills, Meghalaya: An epidemiological perspective', Indian J Med Res, 141: 591-7. |
| Khatun, S., A. Chakraborty, M. Rahman, N. Nasreen Banu, M. M. Rahman, S. M. Hasan, S. P. Luby, and E. S. Gurley. 2015. 'An Outbreak of Chikungunya in Rural Bangladesh, 2011', PLoS Negl Trop Dis, 9: e0003907. |
| Kim Lien, P. T., L. Briant, T. B. Tang, B. M. Trang, L. Gavotte, E. Cornillot, V. T. Duoc, T. N. Duong, R. Frutos, and P. T. Nga. 2016. 'Surveillance of dengue and chikungunya infection in Dong Thap, Vietnam: A 13-month study', Asian Pac J Trop Med, 9: 39-43. |
| Kinimi, Edson, Bisimwa N Patrick, and Gerald Misinzo. 2018. 'Serological evidence of chikungunya and malaria co-infection among febrile patients seeking health care in Karagwe district, Tanzania', Tanzania Journal of Health Research, 20. |
| Kinimi, E., M. J. Shayo, B. N. Patrick, S. O. Angwenyi, C. J. Kasanga, J. Weyer, P. Jansen van Vuren, J. T. Paweska, L. E. G. Mboera, and G. Misinzo. 2018. 'Evidence of chikungunya virus infection among febrile patients seeking healthcare in selected districts of Tanzania', Infect Ecol Epidemiol, 8: 1553460. |
| Kolawole, O. M., K. E. Bello, A. A. Seriki, and A. A. Irekeola. 2017. 'Serological survey of Chikungunya virus in Ilorin Metropolis, Nigeria', Brazilian Journal of Infectious Diseases, 21: 365-66. |
| Konongoi, Samson Limbaso, Albert Nyunja, Victor Ofula, Samuel Owaka, Hellen Koka, Edith Koskei, Fredrick Eyase, Daniel Langat, James Mancuso, and Joel Lutomiah. 2018. 'Human and entomologic investigations of chikungunya outbreak in Mandera, Northeastern Kenya, 2016', PloS one, 13. |
| Kosasih, H., Q. de Mast, S. Widjaja, P. Sudjana, U. Antonjaya, C. Ma'roef, S. F. Riswari, K. R. Porter, T. H. Burgess, B. Alisjahbana, A. van der Ven, and M. Williams. 2013. 'Evidence for endemic chikungunya virus infections in Bandung, Indonesia', PLoS Negl Trop Dis, 7: e2483. |
| Kuan, G., S. Ramirez, L. Gresh, S. Ojeda, M. Melendez, N. Sanchez, D. Collado, N. Garcia, J. C. Mercado, A. Gordon, A. Balmaseda, and E. Harris. 2016. 'Seroprevalence of Anti-Chikungunya Virus Antibodies in Children and Adults in Managua, Nicaragua, After the First Chikungunya Epidemic, 2014-2015', PLoS Negl Trop Dis, 10: e0004773. |
| Kularatne, Senanayake AM, Sajitha C Weerasinghe, Champika Gihan, Sujantha Wickramasinghe, Samath Dharmarathne, Asanka Abeyrathna, and Thilak Jayalath. 2012. 'Epidemiology, clinical manifestations, and long-term outcomes of a major outbreak of chikungunya in a hamlet in Sri Lanka, in 2007: a longitudinal cohort study', Journal of tropical medicine, 2012. |
| Kumar, K., M. Chhabra, R. Katyal, P. K. Patnaik, H. Kukreti, A. Rai, V. K. Saxena, V. Mittal, and S. Lal. 2008. 'Investigation of an outbreak of chikungunya in Malegaon Municipal areas of Nasik district, Maharashtra (India) and its control', J Vector Borne Dis, 45: 157-63. |
| Kumar, CVM Naresh, P Sangamithra, M Rajasekhar, and DVR Saigopal. 2010. 'Surveillance of chikungunya virus in Andhra Pradesh, Southern India', Asian Pacific Journal of Tropical Medicine, 3: 860-65. |
| Kumar, Narendran Pradeep, Abidha Suresh, Perumal Vanamail, Shanmugavelu Sabesan, Kalianna Gounder Krishnamoorthy, Jacob Mathew, Varakilparambil Thomas Jose, and Purushothaman Jambulingam. 2011. 'Chikungunya virus outbreak in Kerala, India, 2007: a seroprevalence study', Memórias do Instituto Oswaldo Cruz, 106: 912-16. |
| Kumar, Alok, Christine Best, and Gemma Benskin. 2017. 'Epidemiology, clinical and laboratory features and course of chikungunya among a cohort of children during the first Caribbean epidemic', Journal of tropical pediatrics, 63: 43-49. |
| Kumarasamy, V., S. Prathapa, H. Zuridah, Y. K. Chem, I. Norizah, and K. B. Chua. 2006. 'Re-emergence of Chikungunya virus in Malaysia', Med J Malaysia, 61: 221-5. |
| Kuniholm, M. H., N. D. Wolfe, C. Y. Huang, E. Mpoudi-Ngole, U. Tamoufe, M. LeBreton, D. S. Burke, and D. J. Gubler. 2006. 'Seroprevalence and distribution of Flaviviridae, Togaviridae, and Bunyaviridae arboviral infections in rural Cameroonian adults', Am J Trop Med Hyg, 74: 1078-83. |
| LaBeaud, A Desiree, Tamara Banda, Julie Brichard, Eric M Muchiri, Peter L Mungai, Francis M Mutuku, Erin Borland, Ginny Gildengorin, Sarah Pfeil, and Crystal Y Teng. 2015. 'High rates of o’nyong nyong and Chikungunya virus transmission in coastal Kenya', PLoS neglected tropical diseases, 9. |
| Lam, S. K., K. B. Chua, P. S. Hooi, M. A. Rahimah, S. Kumari, M. Tharmaratnam, S. K. Chuah, D. W. Smith, and I. A. Sampson. 2001. 'Chikungunya infection--an emerging disease in Malaysia', Southeast Asian J Trop Med Public Health, 32: 447-51. |
| Langsjoen, R. M., R. J. Rubinstein, T. F. Kautz, A. J. Auguste, J. H. Erasmus, L. Kiaty-Figueroa, R. Gerhardt, D. Lin, K. L. Hari, R. Jain, N. Ruiz, A. E. Muruato, J. Silfa, F. Bido, M. Dacso, and S. C. Weaver. 2016. 'Molecular Virologic and Clinical Characteristics of a Chikungunya Fever Outbreak in La Romana, Dominican Republic, 2014', PLoS Negl Trop Dis, 10: e0005189. |
| Laoprasopwattana, K, T Suntharasaj, P Petmanee, O Suddeaugrai, and A Geater. 2016. 'Chikungunya and dengue virus infections during pregnancy: seroprevalence, seroincidence and maternal–fetal transmission, southern Thailand, 2009–2010', Epidemiology & Infection, 144: 381-88. |
| Laras, K., N. C. Sukri, R. P. Larasati, M. J. Bangs, R. Kosim, Djauzi, T. Wandra, J. Master, H. Kosasih, S. Hartati, C. Beckett, E. R. Sedyaningsih, H. J. Beecham, 3rd, and A. L. Corwin. 2005. 'Tracking the re-emergence of epidemic chikungunya virus in Indonesia', Trans R Soc Trop Med Hyg, 99: 128-41. |
| Ledrans, M, F Najioullah, S STEGMANN PLANCHARD, O FLUSIN, and C PRAT. 2014. 'Emergence du chikungunya dans les départements français d'Amérique: organisation et résultats de la surveillance épidémiologique, avril 2014', Bulletin épidémiologique hebdomadaire: 368-79. |
| Leroy, Eric M, Dieudoné Nkoghe, Benjamin Ollomo, Chimène Nze-Nkogue, Pierre Becquart, Gilda Grard, Xavier Pourrut, Rémi Charrel, Grégory Moureau, and Angélique Ndjoyi-Mbiguino. 2009. 'Concurrent chikungunya and dengue virus infections during simultaneous outbreaks, Gabon, 2007', Emerging infectious diseases, 15: 591. |
| Lertanekawattana, Sujet, Surapee Anantapreecha, Chuleeporn Jiraphongsa, Pawinee Duan-ngern, Sathit Potjalongsin, Wisanu Wiittayabamrung, Pamol Daroon, and Meta Techolarn. 2013. 'Prevalence and characteristics of dengue and chikungunya infections among acute febrile patients in Nong Khai Province, Thailand', Southeast Asian J Trop Med Public Health, 44: 780-90. |
| Lizarazo, E., M. Vincenti-Gonzalez, M. E. Grillet, S. Bethencourt, O. Diaz, N. Ojeda, H. Ochoa, M. A. Rangel, and A. Tami. 2019. 'Spatial Dynamics of Chikungunya Virus, Venezuela, 2014', Emerg Infect Dis, 25: 672-80. |
| Londhey, V., S. Agrawal, N. Vaidya, S. Kini, J. S. Shastri, and S. Sunil. 2016. 'Dengue and Chikungunya Virus Co-infections: The Inside Story', J Assoc Physicians India, 64: 36-40. |
| Macpherson, C., T. Noël, P. Fields, D. Jungkind, K. Yearwood, M. Simmons, S. Widjaja, G. Mitchell, D. Noel, S. Bidaisee, T. E. Myers, and A. D. LaBeaud. 2016. 'Clinical and Serological Insights from the Asian Lineage Chikungunya Outbreak in Grenada, 2014: An Observational Study', Am J Trop Med Hyg, 95: 890-93. |
| Makiala-Mandanda, Sheila, Steve Ahuka-Mundeke, Jessica L Abbate, Elisabeth Pukuta-Simbu, Justus Nsio-Mbeta, Nicolas Berthet, Eric Maurice Leroy, Pierre Becquart, and Jean-Jacques Muyembe-Tamfum. 2018. 'Identification of Dengue and Chikungunya cases among suspected cases of Yellow Fever in the Democratic Republic of the Congo', Vector-Borne and Zoonotic Diseases, 18: 364-70. |
| Malik, Mamunur Rahman, Abraham Mnzava, Emad Mohareb, Alia Zayed, Abdulhakeem Al Kohlani, Ahmed AK Thabet, and Hassan El Bushra. 2014. 'Chikungunya outbreak in Al-Hudaydah, Yemen, 2011: epidemiological characterization and key lessons learned for early detection and control', Journal of epidemiology and global health, 4: 203-11. |
| Manimunda, S. P., A. P. Sugunan, S. K. Rai, P. Vijayachari, A. N. Shriram, S. Sharma, N. Muruganandam, I. K. Chaitanya, D. R. Guruprasad, and A. B. Sudeep. 2010. 'Outbreak of chikungunya fever, Dakshina Kannada District, South India, 2008', Am J Trop Med Hyg, 83: 751-4. |
| Manu, S. K., J. H. K. Bonney, D. Pratt, F. N. Abdulai, E. E. Agbosu, P. O. Frimpong, and T. K. Adiku. 2019. 'Arbovirus circulation among febrile patients at the greater Accra Regional Hospital, Ghana', BMC Res Notes, 12: 332. |
| McHale, Thomas, Claudia Romero-Vivas, Claudio Fronterre, Pedro Arango-Padilla, Andrew Falconar, Naomi Waterlow, Chad Nix, and Jorge Cano. 2019. 'Spatiotemporal Heterogeneity in the Distribution of Chikungunya and Zika Virus Case Incidences and Risk Factors During Their Epidemics in Barranquilla, Colombia, between 2014 and 2016: An Ecological Study', Preprints. |
| Mease, L. E., R. L. Coldren, L. A. Musila, T. Prosser, F. Ogolla, V. O. Ofula, R. J. Schoepp, C. A. Rossi, and N. Adungo. 2011. 'Seroprevalence and distribution of arboviral infections among rural Kenyan adults: a cross-sectional study', Virol J, 8: 371. |
| Mehdi, Z., H. Shahbaz, A. Owais, S. U. Hasan, I. Nasr, A. Jahangir, N. Zubair, S. A. Abdul Khaliq, M. Khalid, S. Shahbaz, M. Qureshi, R. Hasan, M. Fasih, A. Khalid, D. Hasan, and S. Nigar. 2019. 'Frequency, Awareness, and Symptoms of Chikungunya Among Patients in a Tertiary Care Hospital of Karachi: A Cross-Sectional Study', Cureus, 11: e4054. |
| Méndez, Nina, Luis Baeza-Herrera, Rafael Ojeda-Baranda, Oswaldo Huchim-Lara, and Salvador Gómez-Carro. 2017. 'Perfil clinicoepidemiológico de la infección por Chikungunya en casos hospitalarios atendidos en 2015 en Mérida, México', Revista Panamericana de Salud Pública, 41: e91. |
| Mercado-Reyes, Marcela, Jorge Acosta-Reyes, Edgar Navarro-Lechuga, Sherill Corchuelo, Angélica Rico, Edgar Parra, Natalia Tolosa, Lissethe Pardo, Maritza González, and Jorge Martìn-Rodriguez-Hernández. 2019. 'Dengue, chikungunya and Zika virus coinfection: results of the national surveillance during the Zika epidemic in Colombia', Epidemiology & Infection, 147. |
| Ministerio del Poder Ciudadano para la Salud de Nicaragua. 2017. 'Seroprevalencia y tasa de ataque clínica por chikungunya en Nicaragua, 2014-2015', Rev. panam. salud pública, 41: e59-e59. |
| Mohamed, N., M. Magzoub, R. E. H. Mohamed, F. S. Aleanizy, F. Y. Alqahtani, B. Y. M. Nour, and M. M. S. Alkarsany. 2019. 'Prevalence and identification of arthropod-transmitted viruses in Kassala state, Eastern Sudan', Libyan J Med, 14: 1564511. |
| Mohanty, I., M. Dash, S. Sahu, M. V. Narasimham, P. Panda, and S. Padhi. 2013. 'Seroprevalence of chikungunya in southern odisha', J Family Med Prim Care, 2: 33-6. |
| Mombouli, J. V., P. Bitsindou, D. O. Elion, A. Grolla, H. Feldmann, F. R. Niama, H. J. Parra, and V. J. Munster. 2013. 'Chikungunya virus infection, Brazzaville, Republic of Congo, 2011', Emerg Infect Dis, 19: 1542-3. |
| Mørch, K., A. Manoharan, S. Chandy, N. Chacko, G. Alvarez-Uria, S. Patil, A. Henry, J. Nesaraj, C. Kuriakose, A. Singh, S. Kurian, C. Gill Haanshuus, N. Langeland, B. Blomberg, G. Vasanthan Antony, and D. Mathai. 2017. 'Acute undifferentiated fever in India: a multicentre study of aetiology and diagnostic accuracy', BMC Infect Dis, 17: 665. |
| Moro, M. L., C. Gagliotti, G. Silvi, R. Angelini, V. Sambri, G. Rezza, E. Massimiliani, A. Mattivi, E. Grilli, A. C. Finarelli, N. Spataro, A. M. Pierro, T. Seyler, and P. Macini. 2010. 'Chikungunya virus in North-Eastern Italy: a seroprevalence survey', Am J Trop Med Hyg, 82: 508-11. |
| Moyen, N., S. D. Thiberville, B. Pastorino, A. Nougairede, L. Thirion, J. V. Mombouli, Y. Dimi, I. Leparc-Goffart, M. R. Capobianchi, A. D. Lepfoundzou, and X. de Lamballerie. 2014. 'First reported chikungunya fever outbreak in the republic of Congo, 2011', PloS one, 9: e115938. |
| Mudurangaplar, B., and B. V. Peerapur. 2015. 'Seroepidemiological Survey of Chikungunya in and Around the Regions of Bijapur (Vijayapura - North Karnataka)', J Clin Diagn Res, 9: Dc01-2. |
| Mugabe, V. A., S. Ali, I. Chelene, V. O. Monteiro, O. Guiliche, A. F. Muianga, F. Mula, V. António, I. Chongo, J. Oludele, K. Falk, I. A. Paploski, M. G. Reis, U. Kitron, B. M. Kümmerer, G. S. Ribeiro, and E. S. Gudo. 2018. 'Evidence for chikungunya and dengue transmission in Quelimane, Mozambique: Results from an investigation of a potential outbreak of chikungunya virus', PloS one, 13: e0192110. |
| Muianga, A., G. Pinto, M. Massangaie, S. Ali, J. Oludele, A. Tivane, K. I. Falk, N. Lagerqvist, and E. S. Gudo. 2018. 'Antibodies Against Chikungunya in Northern Mozambique During Dengue Outbreak, 2014', Vector Borne Zoonotic Dis, 18: 445-49. |
| Mukherjee, S., S. K. Dutta, S. Sengupta, and A. Tripathi. 2017. 'Evidence of dengue and chikungunya virus co-infection and circulation of multiple dengue serotypes in a recent Indian outbreak', Eur J Clin Microbiol Infect Dis, 36: 2273-79. |
| Mulyatno, K. C., H. Susilowati, A. Yamanaka, S. Soegijanto, and E. Konishi. 2012. 'Primary isolation and phylogenetic studies of Chikungunya virus from Surabaya, Indonesia', Jpn J Infect Dis, 65: 92-4. |
| Murhekar, M., K. Kanagasabai, V. Shete, V. Joshua, M. Ravi, B. K. Kirubakaran, R. Ramachandran, R. Sabarinathan, and N. Gupta. 2019. 'Epidemiology of chikungunya based on laboratory surveillance data-India, 2016-2018', Trans R Soc Trop Med Hyg, 113: 259-62. |
| Muyeku–Matilu, Mary Inziani. 'Seroprevalence of Chikungunya, Yellow fever and West Nile Viruses in Children at the Alupe District Hospital in Western Kenya'. |
| Nakkhara, P., V. Chongsuvivatwong, and S. Thammapalo. 2013. 'Risk factors for symptomatic and asymptomatic chikungunya infection', Trans R Soc Trop Med Hyg, 107: 789-96. |
| Naveca, Felipe Gomes, Ingra Claro, Marta Giovanetti, Jaqueline Goes de Jesus, Joilson Xavier, Felipe Campos de Melo Iani, Valdinete Alves do Nascimento, Victor Costa de Souza, Paola Paz Silveira, and José Lourenço. 2019. 'Genomic, epidemiological and digital surveillance of Chikungunya virus in the Brazilian Amazon', PLoS neglected tropical diseases, 13: e0007065. |
| Ngoi, C. N., M. A. Price, B. Fields, J. Bonventure, C. Ochieng, G. Mwashigadi, A. S. Hassan, A. N. Thiong'o, M. Micheni, P. Mugo, S. Graham, and E. J. Sanders. 2016. 'Dengue and Chikungunya Virus Infections among Young Febrile Adults Evaluated for Acute HIV-1 Infection in Coastal Kenya', PloS one, 11: e0167508. |
| Ngwe Tun, M. M., S. Inoue, K. Z. Thant, N. Talemaitoga, A. Aryati, E. M. Dimaano, R. R. Matias, C. C. Buerano, F. F. Natividad, W. Abeyewickreme, N. T. Thuy, L. T. Mai, F. Hasebe, D. Hayasaka, and K. Morita. 2016. 'Retrospective seroepidemiological study of chikungunya infection in South Asia, Southeast Asia and the Pacific region', Epidemiol Infect, 144: 2268-75. |
| Ninla-Aesong, P., W. Mitarnun, and K. Noipha. 2020. 'Long-Term Persistence of Chikungunya Virus-Associated Manifestations and Anti-Chikungunya Virus Antibody in Southern Thailand: 5 Years After an Outbreak in 2008-2009', Viral Immunol, 33: 86-93. |
| Nitatpattana, N., K. Kanjanopas, S. Yoksan, W. Satimai, N. Vongba, S. Langdatsuwan, K. Nakgoi, S. Ratchakum, N. Wauquier, M. Souris, P. Auewarakul, and J. P. Gonzalez. 2014. 'Long-term persistence of Chikungunya virus neutralizing antibodies in human populations of North Eastern Thailand', Virol J, 11: 183. |
| Noridah, O., V. Paranthaman, S. K. Nayar, M. Masliza, K. Ranjit, I. Norizah, Y. K. Chem, B. Mustafa, V. Kumarasamy, and K. B. Chua. 2007. 'Outbreak of chikungunya due to virus of Central/East African genotype in Malaysia', Med J Malaysia, 62: 323-8. |
| Nunes, M. R., N. R. Faria, J. M. de Vasconcelos, N. Golding, M. U. Kraemer, L. F. de Oliveira, S. Azevedo Rdo, D. E. da Silva, E. V. da Silva, S. P. da Silva, V. L. Carvalho, G. E. Coelho, A. C. Cruz, S. G. Rodrigues, J. L. Vianez, Jr., B. T. Nunes, J. F. Cardoso, R. B. Tesh, S. I. Hay, O. G. Pybus, and P. F. Vasconcelos. 2015. 'Emergence and potential for spread of Chikungunya virus in Brazil', BMC Med, 13: 102. |
| Ochieng, Caroline, Petronella Ahenda, Amy Y Vittor, Raymond Nyoka, Stella Gikunju, Cyrus Wachira, Lilian Waiboci, Mamo Umuro, Andrea A Kim, and Leonard Nderitu. 2015. 'Seroprevalence of infections with dengue, Rift Valley fever and chikungunya viruses in Kenya, 2007', PloS one, 10. |
| Olajiga, Olayinka M, Olajumoke E Adesoye, Adewale P Emilolorun, Abiodun J Adeyemi, Emmanuel O Adeyefa, Ismail A Aderibigbe, Salmot A Adejumo, Wasiu O Adebimpe, Oluyinka O Opaleye, and Waidi F Sule. 2017. 'Chikungunya virus seroprevalence and associated factors among hospital attendees in two states of southwest Nigeria: A preliminary assessment', Immunological investigations, 46: 552-65. |
| Oliveira, J. F., M. S. Rodrigues, L. M. Skalinski, A. E. S. Santos, L. C. Costa, L. L. Cardim, E. S. Paixão, Mdcn Costa, W. K. Oliveira, M. L. Barreto, M. G. Teixeira, and R. F. S. Andrade. 2020. 'Interdependence between confirmed and discarded cases of dengue, chikungunya and Zika viruses in Brazil: A multivariate time-series analysis', PloS one, 15: e0228347. |
| Omarjee, R., C. Prat, O. Flusin, S. Boucau, B. Tenebray, O. Merle, P. Huc-Anais, S. Cassadou, and I. Leparc-Goffart. 2014. 'Importance of case definition to monitor ongoing outbreak of chikungunya virus on a background of actively circulating dengue virus, St Martin, December 2013 to January 2014', Euro Surveill, 19. |
| Omatola, Cornelius A., Bernard A. Onoja, Peter K. Fassan, Stephanie A. Osaruyi, Mercy Iyeh, Matthew A. Samuel, and Peace U. Haruna. 2020. 'Seroprevalence of chikungunya virus infection in five hospitals within Anyigba, Kogi State of Nigeria', Brazilian Journal of Infectious Diseases, 24: 1-6. |
| Oviedo-Pastrana, M., N. Méndez, S. Mattar, G. Arrieta, and L. Gomezcaceres. 2017. 'Epidemic outbreak of Chikungunya in two neighboring towns in the Colombian Caribbean: a survival analysis', Arch Public Health, 75: 1. |
| Oviedo-Pastrana, M., N. Méndez, S. Mattar, G. Arrieta, and L. Gomezcaceres. 2018. 'Lessons learned of emerging Chikungunya virus in two populations of social vulnerability of the Colombian tropics: epidemiological analysis', Arch Public Health, 76: 36. |
| Padbidri, V. S., N. S. Wairagkar, G. D. Joshi, U. B. Umarani, A. R. Risbud, D. L. Gaikwad, S. S. Bedekar, A. D. Divekar, and F. M. Rodrigues. 2002. 'A serological survey of arboviral diseases among the human population of the Andaman and Nicobar Islands, India', Southeast Asian J Trop Med Public Health, 33: 794-800. |
| Padilla, Julio César, Fredy Eberto Lizarazo, Olga Lucía Murillo, Fernando Antonio Mendigaña, Edwin Pachón, and Mauricio Javier Vera. 2017. 'Epidemiología de las principales enfermedades transmitidas por vectores en Colombia, 1990-2016', Biomédica, 37: 27-40. |
| Paganin, F., G. Borgherini, F. Staikowsky, C. Arvin-Berod, and P. Poubeau. 2006. '[Chikungunya on Reunion Island: chronicle of an epidemic foretold]', Presse Med, 35: 641-6. |
| Pan American Health Organization. 2014. "Epidemiological Update Chikungunya fever 23 May 2014." In. |
| Pan American Health Organization. 2015. "Number of Reported Cases of Chikungunya Fever in the Americas, by Country or Territory 2015." In. |
| Pan American Health Organization. 2017. "Number of Reported Cases of Chikungunya Fever in the Americas, by Country or Territory 2016." In. |
| Pan American Health Organization. 2017. "Number of Reported Cases of Chikungunya Fever in the Americas, by Country or Territory 2017." In. |
| an American Health Organization. Cases of Chikungunya Virus Disease 2020 [Available from: https://www.paho.org/data/index.php/en/mnu-topics/chikv-en/550-chikv-weekly-en.html. |
| Pandey, B. D., B. Neupane, K. Pandey, M. M. Tun, and K. Morita. 2015. 'Detection of Chikungunya Virus in Nepal', Am J Trop Med Hyg, 93: 697-700. |
| Pandey, Kishor, Basu Dev Pandey, Ram Rekha Chaurasiya, Mahesh Thakur, Biswas Neupane, Yogendra Shah, Mya Myat Ngwe Tun, and Kouichi Morita. 2017. 'Evidence of Chikungunya virus circulation in the Terai region of Nepal in 2014 and 2015', Transactions of the Royal Society of Tropical Medicine and Hygiene, 111: 294-99. |
| Panning, Marcus, Dominic Wichmann, Klaus Grywna, Augustina Annan, Sriyal Wijesinghe, SAM Kularatne, and Christian Drosten. 2009. 'No evidence of chikungunya virus and antibodies shortly before the outbreak on Sri Lanka', Medical microbiology and immunology, 198: 103. |
| Paquet, Christophe, I Quatresous, JL Solet, D Sissoko, P Renault, V Pierre, H Cordel, C Lassalle, J Thiria, and H Zeller. 2006. 'Chikungunya outbreak in Reunion: epidemiology and surveillance, 2005 to early January 2006', Weekly releases (1997–2007), 11: 2891. |
| Pastorino, B., J. J. Muyembe-Tamfum, M. Bessaud, F. Tock, H. Tolou, J. P. Durand, and C. N. Peyrefitte. 2004. 'Epidemic resurgence of Chikungunya virus in democratic Republic of the Congo: identification of a new central African strain', J Med Virol, 74: 277-82. |
| Pastula, D. M., W. T. Hancock, M. Bel, H. Biggs, M. Marfel, R. Lanciotti, J. Laven, T. H. Chen, J. E. Staples, M. Fischer, and S. L. Hills. 2017. 'Chikungunya virus disease outbreak in Yap State, Federated States of Micronesia', PLoS Negl Trop Dis, 11: e0005410. |
| Patil, S. S., S. R. Patil, P. M. Durgawale, and A. G. Patil. 2013. 'A study of the outbreak of Chikungunya fever', J Clin Diagn Res, 7: 1059-62. |
| Patrick, BN, S Angwenyi, E Kinimi, M Shayo, M Hugo, and CJ Kasanga. 2018. 'Evidence of anti-chikungunya virus igg and igm antibodies among patients seeking treatment in different health facilities in Kyela District, Tanzania'. |
| Paul, K. K., H. Salje, M. W. Rahman, M. Rahman, and E. S. Gurley. 2020. 'Comparing insights from clinic-based versus community-based outbreak investigations: a case study of chikungunya in Bangladesh', Int J Infect Dis. |
| Pereira Gusmão Maia, Z., F. Mota Pereira, R. F. do Carmo Said, V. Fonseca, T. Gräf, F. de Bruycker Nogueira, V. Brandão Nardy, J. Xavier, M. Lima Maia, A. L. Abreu, C. F. Campelo de Albuquerque, W. Kleber Oliveira, J. Croda, A. M. B. de Filippis, R. Venancio Cunha, J. Lourenço, T. de Oliveira, N. R. Faria, L. C. Junior Alcantara, and M. Giovanetti. 2020. 'Return of the founder Chikungunya virus to its place of introduction into Brazil is revealed by genomic characterization of exanthematic disease cases', Emerg Microbes Infect, 9: 53-57. |
| Pessôa, Rodrigo, João Veras Patriota, Maria de Lourdes de Souza, Alvina Clara Felix, Nubia Mamede, and Sabri S Sanabani. 2016. 'Investigation into an outbreak of dengue-like illness in Pernambuco, Brazil, revealed a cocirculation of Zika, Chikungunya, and dengue virus type 1', Medicine, 95. |
| Peyrefitte, C. N., D. Rousset, B. A. Pastorino, R. Pouillot, M. Bessaud, F. Tock, H. Mansaray, O. L. Merle, A. M. Pascual, C. Paupy, A. Vessiere, P. Imbert, P. Tchendjou, J. P. Durand, H. J. Tolou, and M. Grandadam. 2007. 'Chikungunya virus, Cameroon, 2006', Emerg Infect Dis, 13: 768-71. |
| Peyrefitte, C. N., M. Bessaud, B. A. Pastorino, P. Gravier, S. Plumet, O. L. Merle, I. Moltini, E. Coppin, F. Tock, W. Daries, L. Ollivier, F. Pages, R. Martin, F. Boniface, H. J. Tolou, and M. Grandadam. 2008. 'Circulation of Chikungunya virus in Gabon, 2006-2007', J Med Virol, 80: 430-3. |
| Pham, P. N., L. T. Williams, U. Obot, L. A. Padilla, M. Aung, T. F. Akinyemiju, A. P. Carson, and P. E. Jolly. 2017. 'Epidemiology of Chikungunya fever outbreak in Western Jamaica during July-December 2014', Res Rep Trop Med, 8: 7-16. |
| Phommanivong, V., S. Kanda, T. Shimono, P. Lamaningao, A. W. Darcy, N. Mishima, B. Phaytanavanh, and T. Nishiyama. 2016. 'Co-circulation of the dengue with chikungunya virus during the 2013 outbreak in the southern part of Lao PDR', Trop Med Health, 44: 24. |
| Poirier, Mathieu JP, Delynn M Moss, Karla R Feeser, Thomas G Streit, Gwong-Jen J Chang, Matthew Whitney, Brandy J Russell, Barbara W Johnson, Alison J Basile, Christin H Goodman, Amanda K Barry, and Patrick J Lammie. 2016. 'Measuring Haitian children’s exposure to chikungunya, dengue and malaria', Bulletin of the World Health Organization, 94: 817-25A. |
| Porter, K. R., R. Tan, Y. Istary, W. Suharyono, Sutaryo, S. Widjaja, C. Ma'Roef, E. Listiyaningsih, H. Kosasih, L. Hueston, J. McArdle, and M. Juffrie. 2004. 'A serological study of Chikungunya virus transmission in Yogyakarta, Indonesia: evidence for the first outbreak since 1982', Southeast Asian J Trop Med Public Health, 35: 408-15. |
| Proesmans, Sam, Freddy Katshongo, John Milambu, Blaise Fungula, Hypolite Muhindo Mavoko, Steve Ahuka-Mundeke, Raquel Inocêncio da Luz, Marjan Van Esbroeck, Kevin K Ariën, and Lieselotte Cnops. 2019. 'Dengue and chikungunya among outpatients with acute undifferentiated fever in Kinshasa, Democratic Republic of Congo: A cross-sectional study', PLoS neglected tropical diseases, 13: e0007047. |
| Qiaoli, Z., H. Jianfeng, W. De, W. Zijun, Z. Xinguang, Z. Haojie, D. Fan, L. Zhiquan, W. Shiwen, H. Zhenyu, Z. Yonghui, K. Changwen, Y. Dakang, L. Wenjia, L. Deqiong, and C. Pinghua. 2012. 'Maiden outbreak of chikungunya in Dongguan city, Guangdong province, China: epidemiological characteristics', PloS one, 7: e42830. |
| Quyen, N. T. H., D. T. H. Kien, M. Rabaa, N. M. Tuan, T. T. Vi, L. Van Tan, N. T. Hung, H. M. Tuan, T. Van Tram, N. Le Da Ha, H. K. Quang, N. Q. Doanh, N. Van Vinh Chau, B. Wills, and C. P. Simmons. 2017. 'Chikungunya and Zika Virus Cases Detected against a Backdrop of Endemic Dengue Transmission in Vietnam', Am J Trop Med Hyg, 97: 146-50. |
| Raghavendhar, B. S., P. Ray, V. H. Ratagiri, B. S. Sharma, S. K. Kabra, and R. Lodha. 2016. 'Evaluation of chikungunya virus infection in children from India during 2009-2010: A cross sectional observational study', J Med Virol, 88: 923-30. |
| Rahman, M., J. Yamagishi, R. Rahim, A. Hasan, and A. Sobhan. 2019. 'East/Central/South African Genotype in a Chikungunya Outbreak, Dhaka, Bangladesh, 2017', Emerg Infect Dis, 25: 370-72. |
| Raina, S., R. K. Raina, N. Agarwala, S. K. Raina, and R. Sharma. 2018. 'Coinfections as an aetiology of acute undifferentiated febrile illness among adult patients in the sub-Himalayan region of north India', J Vector Borne Dis, 55: 130-36. |
| Ramachandran, V. G., S. Das, P. Roy, V. Hada, and N. S. Mogha. 2016. 'Chikungunya: a reemerging infection spreading during 2010 dengue fever outbreak in National Capital Region of India', Virusdisease, 27: 183-6. |
| Rao, P. N., A. M. van Eijk, S. Choubey, S. Z. Ali, A. Dash, P. Barla, R. R. Oraon, G. Patel, P. Nandini, S. Acharya, S. Mohanty, J. M. Carlton, and S. Satpathi. 2019. 'Dengue, chikungunya, and scrub typhus are important etiologies of non-malarial febrile illness in Rourkela, Odisha, India', BMC Infect Dis, 19: 572. |
| Ratsitorahina, Mahery, Julie Harisoa, Jocelyn Ratovonjato, Sophie Biacabe, Jean-Marc Reynes, Hervé Zeller, Yolande Raoelina, Antoine Talarmin, Vincent Richard, and Jean Louis Soares. 2008. 'Outbreak of dengue and Chikungunya fevers, Toamasina, Madagascar, 2006', Emerging infectious diseases, 14: 1135. |
| Ray, P., V. H. Ratagiri, S. K. Kabra, R. Lodha, S. Sharma, B. S. Sharma, M. Kalaivani, and N. Wig. 2012. 'Chikungunya infection in India: results of a prospective hospital based multi-centric study', PloS one, 7: e30025. |
| Reller, M. E., U. Akoroda, A. Nagahawatte, V. Devasiri, W. Kodikaarachchi, J. J. Strouse, R. Chua, Y. Hou, A. Chow, O. M. Sessions, T. Østbye, D. J. Gubler, C. W. Woods, and C. Bodinayake. 2013. 'Chikungunya as a cause of acute febrile illness in southern Sri Lanka', PloS one, 8: e82259. |
| Renault, Philippe, Jean-Louis Solet, Daouda Sissoko, Elsa Balleydier, Sophie Larrieu, Laurent Filleul, Christian Lassalle, Julien Thiria, Emmanuelle Rachou, and Henriette de Valk. 2007. 'A major epidemic of chikungunya virus infection on Reunion Island, France, 2005–2006', The American journal of tropical medicine and hygiene, 77: 727-31. |
| Renault, P, D Sissoko, M Ledrans, V Pierre, and G Brücker. 2008. 'L’épidémie de chikungunya aLa Réunion et aMayotte, France, 2005–2006: le contexte et les questions de surveillance et d’évaluation posées', Bull Epidémiol Hebd: 38-39. |
| Réunion-Mayotte, Cire. Épidémiologie du chikungunya à la Réunion–Bilan d’une année de surveillance–19 avril 2007–18 avril 2008. Institut de veille sanitaire, décembre 2008, 8 p.[Article in French]. |
| Rezza, G., L. Nicoletti, R. Angelini, R. Romi, A. C. Finarelli, M. Panning, P. Cordioli, C. Fortuna, S. Boros, F. Magurano, G. Silvi, P. Angelini, M. Dottori, M. G. Ciufolini, G. C. Majori, and A. Cassone. 2007. 'Infection with chikungunya virus in Italy: an outbreak in a temperate region', Lancet, 370: 1840-6. |
| Rezza, G., G. El-Sawaf, G. Faggioni, F. Vescio, R. Al Ameri, R. De Santis, G. Helaly, A. Pomponi, D. Metwally, M. Fantini, H. Qadi, M. Ciccozzi, and F. Lista. 2014. 'Co-circulation of Dengue and Chikungunya Viruses, Al Hudaydah, Yemen, 2012', Emerg Infect Dis, 20: 1351-4. |
| Rianthavorn, P., K. Prianantathavorn, N. Wuttirattanakowit, A. Theamboonlers, and Y. Poovorawan. 2010. 'An outbreak of chikungunya in southern Thailand from 2008 to 2009 caused by African strains with A226V mutation', Int J Infect Dis, 14: e161-5. |
| Rico-Mendoza, A., P. R. Alexandra, A. Chang, L. Encinales, and R. Lynch. 2019. 'Co-circulation of dengue, chikungunya, and Zika viruses in Colombia from 2008 to 2018', Rev Panam Salud Publica, 43: e49. |
| Riswari, S. F., C. N. Ma'roef, H. Djauhari, H. Kosasih, A. Perkasa, F. A. Yudhaputri, I. M. Artika, M. Williams, A. van der Ven, K. S. Myint, B. Alisjahbana, J. P. Ledermann, A. M. Powers, and U. A. Jaya. 2016. 'Study of viremic profile in febrile specimens of chikungunya in Bandung, Indonesia', J Clin Virol, 74: 61-5. |
| Robinson, M. L., D. Kadam, S. Khadse, U. Balasubramanian, P. Raichur, C. Valvi, I. Marbaniang, S. Kanade, J. Sachs, A. Basavaraj, R. Bharadwaj, A. Kagal, V. Kulkarni, J. Zenilman, G. Nelson, Y. C. Manabe, A. Kinikar, A. Gupta, and V. Mave. 2018. 'Vector-Borne Disease is a Common Cause of Hospitalized Febrile Illness in India', Am J Trop Med Hyg, 98: 1526-33. |
| Rodas, J. D., T. Kautz, E. Camacho, L. Paternina, H. Guzmán, F. J. Díaz, P. Blanco, R. Tesh, and S. C. Weaver. 2016. 'Genetic Characterization of Northwestern Colombian Chikungunya Virus Strains from the 2014-2015 Epidemic', Am J Trop Med Hyg, 95: 639-46. |
| Rodrigues, N. C. P., R. P. Daumas, A. S. de Almeida, R. S. Dos Santos, I. Koster, P. P. Rodrigues, M. F. Gomes, A. F. Macedo, A. Gerardi, and I. D. C. Leite. 2018. 'Risk factors for arbovirus infections in a low-income community of Rio de Janeiro, Brazil, 2015-2016', PloS one, 13: e0198357. |
| Rodríguez-Barraquer, I., S. S. Solomon, P. Kuganantham, A. K. Srikrishnan, C. K. Vasudevan, S. H. Iqbal, P. Balakrishnan, S. Solomon, S. H. Mehta, and D. A. Cummings. 2015. 'The Hidden Burden of Dengue and Chikungunya in Chennai, India', PLoS Negl Trop Dis, 9: e0003906. |
| Rojas, D. P., G. A. Barrera-Fuentes, N. Pavia-Ruz, M. Salgado-Rodriguez, A. Che-Mendoza, P. Manrique-Saide, G. M. Vazquez-Prokopec, M. E. Halloran, I. M. Longini, and H. Gomez-Dantes. 2018. 'Epidemiology of dengue and other arboviruses in a cohort of school children and their families in Yucatan, Mexico: Baseline and first year follow-up', PLoS Negl Trop Dis, 12: e0006847. |
| Rosenberg, Ronald, Nicole P Lindsey, Marc Fischer, Christopher J Gregory, Alison F Hinckley, Paul S Mead, Gabriela Paz-Bailey, Stephen H Waterman, Naomi A Drexler, and Gilbert J Kersh. 2018. 'Vital signs: trends in reported vectorborne disease cases—United States and Territories, 2004–2016', Morbidity and Mortality Weekly Report, 67: 496. |
| Rueda, J. C., A. M. Santos, J. I. Angarita, R. B. Giraldo, E. L. Saldarriaga, J. G. Ballesteros Muñoz, E. Forero, H. Valencia, F. Somoza, D. Martin-Arsanios, E. J. Quintero, V. Reyes-Martinez, D. Padilla, F. M. Cuervo, I. Peláez-Ballestas, M. H. Cardiel, P. X. Pavía, and J. Londono. 2019. 'Demographic and clinical characteristics of chikungunya patients from six Colombian cities, 2014-2015', Emerg Microbes Infect, 8: 1490-500. |
| Safronetz, D., M. Sacko, N. Sogoba, K. Rosenke, C. Martellaro, S. Traoré, I. Cissé, O. Maiga, M. Boisen, D. Nelson, D. Oottamasathien, M. Millett, R. F. Garry, L. M. Branco, S. Doumbia, H. Feldmann, and M. S. Traoré. 2016. 'Vectorborne Infections, Mali', Emerg Infect Dis, 22: 340-2. |
| Sahadeo, N., H. Mohammed, O. M. Allicock, A. J. Auguste, S. G. Widen, K. Badal, K. Pulchan, J. E. Foster, S. C. Weaver, and C. V. Carrington. 2015. 'Molecular Characterisation of Chikungunya Virus Infections in Trinidad and Comparison of Clinical and Laboratory Features with Dengue and Other Acute Febrile Cases', PLoS Negl Trop Dis, 9: e0004199. |
| Saifeldin, Tasabih, Mawahib H Eldegail, Najla A Elaziz, Hala A Alebaeid, and Baha Eldin K Elamin. 2018. 'Co-circulation of Chikungunya virus and Dengue virus in Kassala, Sudan'. |
| Sam, I. C., Y. F. Chan, S. Y. Chan, S. K. Loong, H. K. Chin, P. S. Hooi, R. Ganeswrie, and S. Abubakar. 2009. 'Chikungunya virus of Asian and Central/East African genotypes in Malaysia', J Clin Virol, 46: 180-3. |
| Santhosh, S. R., P. K. Dash, M. M. Parida, M. Khan, M. Tiwari, and P. V. Lakshmana Rao. 2008. 'Comparative full genome analysis revealed E1: A226V shift in 2007 Indian Chikungunya virus isolates', Virus Res, 135: 36-41. |
| Santhosh, S. R., P. K. Dash, M. Parida, M. Khan, and P. V. Rao. 2009. 'Appearance of E1: A226V mutant Chikungunya virus in Coastal Karnataka, India during 2008 outbreak', Virol J, 6: 172. |
| Sarangan, G., S. A. Nayar, M. Mani, S. Sundarrajan, S. Sankar, G. Palani, G. F. Selvaraj, J. Damodharan, K. Muthumani, and P. Srikanth. 2018. 'Genetic characterization of Chikungunya virus 2009 isolates from South India', Bioinformation, 14: 106-12. |
| Sardi, S. I., S. Somasekar, S. N. Naccache, A. C. Bandeira, L. B. Tauro, G. S. Campos, and C. Y. Chiu. 2016. 'Coinfections of Zika and Chikungunya Viruses in Bahia, Brazil, Identified by Metagenomic Next-Generation Sequencing', J Clin Microbiol, 54: 2348-53. |
| Sari, K., K. S. A. Myint, A. R. Andayani, P. D. Adi, R. Dhenni, A. Perkasa, C. N. Ma'roef, N. P. D. Witari, D. Megawati, A. M. Powers, and U. A. Jaya. 2017. 'Chikungunya fever outbreak identified in North Bali, Indonesia', Trans R Soc Trop Med Hyg, 111: 325-27. |
| Sasayama, M., S. Benjathummarak, N. Kawashita, P. Rukmanee, S. Sangmukdanun, P. Masrinoul, P. Pitaksajjakul, O. Puiprom, P. Wuthisen, T. Kurosu, P. Chaichana, P. Maneekan, K. Ikuta, P. Ramasoota, T. Okabayashi, P. Singhasivanon, and N. Luplertlop. 2014. 'Chikungunya virus was isolated in Thailand, 2010', Virus Genes, 49: 485-9. |
| Sasmono, R. T., A. Perkasa, B. Yohan, S. Haryanto, F. A. Yudhaputri, R. F. Hayati, C. N. Ma'roef, J. P. Ledermann, K. S. Aye Myint, and A. M. Powers. 2017. 'Chikungunya Detection during Dengue Outbreak in Sumatra, Indonesia: Clinical Manifestations and Virological Profile', Am J Trop Med Hyg, 97: 1393-98. |
| Saswat, T., A. Kumar, S. Kumar, P. Mamidi, S. Muduli, N. K. Debata, N. S. Pal, B. M. Pratheek, S. Chattopadhyay, and S. Chattopadhyay. 2015. 'High rates of co-infection of Dengue and Chikungunya virus in Odisha and Maharashtra, India during 2013', Infect Genet Evol, 35: 134-41. |
| Saswat, T., N. Sahoo, S. Muduli, N. K. Debata, S. Chattopadhyay, and S. Chattopadhyay. 2019. 'Epidemiological trends and molecular dynamics of dengue, chikungunya virus infection, coinfection, and other undifferentiated fever during 2015-2016 in Odisha, India', J Med Virol, 91: 163-70. |
| Schwarz, Norbert G, Mirko Girmann, Njary Randriamampionona, Alexandra Bialonski, Deborah Maus, Anne Caroline Krefis, Christine Njarasoa, Jeanne Fleury Rajanalison, Herly Daniel Ramandrisoa, and Maurice Lucien Randriarison. 2012. 'Seroprevalence of antibodies against Chikungunya, Dengue, and Rift Valley fever viruses after febrile illness outbreak, Madagascar', Emerging infectious diseases, 18: 1780. |
| Seck, M. C., A. S. Badiane, J. Thwing, D. Moss, F. B. Fall, J. F. Gomis, A. B. Deme, K. Diongue, M. Sy, A. Mbaye, T. Ndiaye, A. Gaye, Y. D. Ndiaye, M. A. Diallo, D. Ndiaye, and E. Rogier. 2019. 'Serological Data Shows Low Levels of Chikungunya Exposure in Senegalese Nomadic Pastoralists', Pathogens, 8. |
| Sengupta, S., S. Mukherjee, S. K. Haldar, N. Bhattacharya, and A. Tripathi. 2020. 'Re-emergence of Chikungunya virus infection in Eastern India', Braz J Microbiol, 51: 177-82. |
| Sergon, K., A. A. Yahaya, J. Brown, S. A. Bedja, M. Mlindasse, N. Agata, Y. Allaranger, M. D. Ball, A. M. Powers, V. Ofula, C. Onyango, L. S. Konongoi, R. Sang, M. K. Njenga, and R. F. Breiman. 2007. 'Seroprevalence of Chikungunya virus infection on Grande Comore Island, union of the Comoros, 2005', Am J Trop Med Hyg, 76: 1189-93. |
| Sergon, K., C. Njuguna, R. Kalani, V. Ofula, C. Onyango, L. S. Konongoi, S. Bedno, H. Burke, A. M. Dumilla, J. Konde, M. K. Njenga, R. Sang, and R. F. Breiman. 2008. 'Seroprevalence of Chikungunya virus (CHIKV) infection on Lamu Island, Kenya, October 2004', Am J Trop Med Hyg, 78: 333-7. |
| Seruyange, E., K. Ljungberg, C. M. Muvunyi, J. B. Gahutu, S. Katare, J. Nyamusore, Y. D. Gwon, M. Evander, H. Norder, P. Liljeström, and T. Bergström. 2019. 'Seroreactivity to Chikungunya and West Nile Viruses in Rwandan Blood Donors', Vector Borne Zoonotic Dis, 19: 731-40. |
| Seyler, T, Y Hutin, V Ramanchandran, R Ramakrishnan, P Manickam, and M Murhekar. 2010. 'Estimating the burden of disease and the economic cost attributable to chikungunya, Andhra Pradesh, India, 2005–2006', Transactions of the Royal Society of Tropical Medicine and Hygiene, 104: 133-38. |
| Seyler, T., P. Sakdapolrak, S. S. Prasad, and R. Dhanraj. 2012. 'A chikungunya outbreak in the metropolis of Chennai, India, 2006', J Environ Health, 74: 8-13 |
| Shaikh, N, CG Raut, and M Manjunatha. 2015. 'Co-infections with chikungunya and dengue viruses: A serological study in Karnataka State, India', Indian journal of medical microbiology, 33: 459. |
| Sharp, Tyler M, Kyle R Ryff, Luisa Alvarado, Wun-Ju Shieh, Sherif R Zaki, Harold S Margolis, and Brenda Rivera-Garcia. 2016. 'Surveillance for chikungunya and dengue during the first year of chikungunya virus circulation in Puerto Rico', The Journal of infectious diseases, 214: S475-S81. |
| Shilpa, C., K. Kavitha, N. Sudheesh, S. Sabeena, V. Prasad, M. Hindol, and G. Arunkumar. 2020. 'Estimating the seroprevalence of chikungunya virus exposure in Shimoga district, Karnataka state: A hospital-based study during 2014-2018', J Med Virol, 92: 119-23. |
| Silva, M. M. O., L. B. Tauro, M. Kikuti, R. O. Anjos, V. C. Santos, T. S. F. Gonçalves, I. A. D. Paploski, P. S. S. Moreira, L. C. J. Nascimento, G. S. Campos, A. I. Ko, S. C. Weaver, M. G. Reis, U. Kitron, and G. S. Ribeiro. 2019. 'Concomitant Transmission of Dengue, Chikungunya, and Zika Viruses in Brazil: Clinical and Epidemiological Findings From Surveillance for Acute Febrile Illness', Clin Infect Dis, 69: 1353-59. |
| Silva, N. M. D., R. A. G. Teixeira, C. G. Cardoso, J. B. Siqueira Junior, G. E. Coelho, and E. S. F. Oliveira. 2018. 'Chikungunya surveillance in Brazil: challenges in the context of Public Health', Epidemiol Serv Saude, 27: e2017127. |
| Simião, A. R., F. K. A. Barreto, Rmab Oliveira, J. W. Cavalcante, A. S. Lima Neto, R. B. Barbosa, C. S. Lins, A. G. Meira, F. M. C. Araújo, D. R. Q. Lemos, C. H. Alencar, and L. P. G. Cavalcanti. 2019. 'A major chikungunya epidemic with high mortality in northeastern Brazil', Rev Soc Bras Med Trop, 52: e20190266. |
| MOH Singapore. 2014. Vector-borne diseases 2014 (https://www.moh.gov.sg/docs/librariesprovider5/resources-statistics/reports/vector-borne-diseases.pdf). |
| MOH Singapore. 2016. VECTOR-BORNE DISEASES 2016 (https://www.moh.gov.sg/docs/librariesprovider5/resources-statistics/reports/vector-borne-diseases.pdf). |
| MOH Singapore. 2017. Vector-borne diseases 2017 (https://www.moh.gov.sg/docs/librariesprovider5/diseases-updates/vector-bornezoonotic-diseases-2017.pdf). |
| Singh, K., S. K. Sidhu, P. Devi, M. Kaur, M. Kaur, and N. Singh. 2016. 'Seroprevalence of Common Viral Diseases: A Hospital Based Study from Amritsar, India', J Clin Diagn Res, 10: Dc15-dc19. |
| Singh, N., M. Shukla, G. Chand, P. V. Barde, and M. P. Singh. 2014. 'Vector-borne diseases in central India, with reference to malaria, filaria, dengue and chikungunya', WHO South East Asia J Public Health, 3: 28-35. |
| Singh, P., V. Mittal, M. A. Rizvi, D. Bhattacharya, M. Chhabra, D. S. Rawat, R. L. Ichhpujani, L. S. Chauhan, and A. Rai. 2012. 'Northward movement of East Central South African genotype of Chikungunya virus causing an epidemic between 2006-2010 in India', J Infect Dev Ctries, 6: 563-71. |
| Singh, P., V. Mittal, M. M. Rizvi, M. Chhabra, P. Sharma, D. S. Rawat, D. Bhattacharya, L. S. Chauhan, and A. Rai. 2012. 'The first dominant co-circulation of both dengue and chikungunya viruses during the post-monsoon period of 2010 in Delhi, India', Epidemiol Infect, 140: 1337-42. |
| Singh, R. K., S. Tiwari, V. K. Mishra, R. Tiwari, and T. N. Dhole. 2012. 'Molecular epidemiology of Chikungunya virus: mutation in E1 gene region', J Virol Methods, 185: 213-20. |
| Sissoko D, Delmas G, Giry C, Petinelli F, Saidali R, Gabrie P, Abaine A, Paquet C, Pierre V. 2007. 'Épidémie massive de fièvre chikungunya à Mayotte, France en 2005-2006 : description à partir des résultats de deux enquêtes épidémiologiques', Bulletin épidémiologique hebdomadaire, 48: 405-7. |
| Sissoko, D., D. Malvy, C. Giry, G. Delmas, C. Paquet, P. Gabrie, F. Pettinelli, M. A. Sanquer, and V. Pierre. 2008. 'Outbreak of Chikungunya fever in Mayotte, Comoros archipelago, 2005-2006', Trans R Soc Trop Med Hyg, 102: 780-6. |
| Sissoko, D., A. Moendandze, D. Malvy, C. Giry, K. Ezzedine, J. L. Solet, and V. Pierre. 2008. 'Seroprevalence and risk factors of chikungunya virus infection in Mayotte, Indian Ocean, 2005-2006: a population-based survey', PloS one, 3: e3066. |
| Sitepu, Frans, and Elpiani Depari. 2019. 'Epidemiological and Entomological Investigation of Chikungunya Fever Outbreak, in Serdang Bedagai District, North Sumatera Province, Indonesia, 2013', Global Biosecurity, 1. |
| Slavov, Svetoslav Nanev, Katia Kaori Otaguiri, Melina Lellis Bianquini, Hellen Tayana Oliveira Bitencourt, Marcia Cristina Munhoz Chagas, Domingos Sávio de Souza Guerreiro, Luiz Tadeu Moraes Figueiredo, Dimas Tadeu Covas, and Simone Kashima. 2018. 'Seroprevalence of Chikungunya virus in blood donors from Northern and Southeastern Brazil', Hematology, Transfusion and Cell Therapy, 40: 358-62. |
| Soni, M., A. K. Singh, S. Sharma, A. Agarwal, N. Gopalan, P. V. Rao, M. Parida, and P. K. Dash. 2013. 'Molecular and virological investigation of a focal chikungunya outbreak in northern India', ScientificWorldJournal, 2013: 367382. |
| Soulaphy, C., P. Souliphone, K. Phanthavong, D. Phonekeo, S. Phimmasine, B. Khamphaphongphane, V. Kitthiphong, and H. C. Lewis. 2013. 'Emergence of chikungunya in Moonlapamok and Khong Districts, Champassak Province,the Lao People's Democratic Republic, May to September 2012', Western Pac Surveill Response J, 4: 46-50. |
| Souza, Thiago Moreno L, Yasmine Rangel Vieira, Edson Delatorre, Giselle Barbosa-Lima, Raul Leal Faria Luiz, Alexandre Vizzoni, Komal Jain, Milene Mesquita Miranda, Nishit Bhuva, and Jan F Gogarten. 2019. 'Emergence of the East-Central-South-African genotype of Chikungunya virus in Brazil and the city of Rio de Janeiro may have occurred years before surveillance detection', Scientific reports, 9: 1-7. |
| Sow, A., C. Loucoubar, D. Diallo, O. Faye, Y. Ndiaye, C. S. Senghor, A. T. Dia, O. Faye, S. C. Weaver, M. Diallo, D. Malvy, and A. A. Sall. 2016. 'Concurrent malaria and arbovirus infections in Kedougou, southeastern Senegal', Malar J, 15: 47. |
| Sow, A., O. Faye, M. Diallo, D. Diallo, R. Chen, O. Faye, C. T. Diagne, M. Guerbois, M. Weidmann, Y. Ndiaye, C. S. Senghor, A. Faye, O. M. Diop, B. Sadio, O. Ndiaye, D. Watts, K. A. Hanley, A. T. Dia, D. Malvy, S. C. Weaver, and A. A. Sall. 2018. 'Chikungunya Outbreak in Kedougou, Southeastern Senegal in 2009-2010', Open Forum Infect Dis, 5: ofx259. |
| Sow, Abdourahmane, Birgit Nikolay, Oumar Faye, Simon Cauchemez, Jorge Cano, Mawlouth Diallo, Ousmane Faye, Bakary Sadio, Oumar Ndiaye, and Scott C Weaver. 2020. 'Changes in the transmission dynamic of Chikungunya virus in Southeastern Senegal', Viruses, 12: 196. |
| Srikanth, P., G. Sarangan, K. Mallilankaraman, S. A. Nayar, R. Barani, T. Mattew, G. F. Selvaraj, K. A. Sheriff, G. Palani, and K. Muthumani. 2010. 'Molecular characterization of Chikungunya virus during an outbreak in South India', Indian J Med Microbiol, 28: 299-302. |
| Srikiatkhachorn, A., M. T. Alera, C. B. Lago, I. A. Tac-An, D. Villa, S. Fernandez, B. Thaisomboonsuk, C. Klungthong, J. W. Levy, J. M. Velasco, V. G. Roque, Jr., A. Nisalak, L. R. Macareo, and I. K. Yoon. 2016. 'Resolution of a Chikungunya Outbreak in a Prospective Cohort, Cebu, Philippines, 2012-2014', Emerg Infect Dis, 22: 1852-4. |
| Staikowsky, F., K. Le Roux, I. Schuffenecker, P. Laurent, P. Grivard, A. Develay, and A. Michault. 2008. 'Retrospective survey of Chikungunya disease in Réunion Island hospital staff', Epidemiol Infect, 136: 196-206. |
| Staikowsky, F., F. Talarmin, P. Grivard, A. Souab, I. Schuffenecker, K. Le Roux, M. Lecuit, and A. Michault. 2009. 'Prospective study of Chikungunya virus acute infection in the Island of La Réunion during the 2005-2006 outbreak', PloS one, 4: e7603. |
| Stewart-Ibarra, Anna M, Sadie J Ryan, Aileen Kenneson, Christine A King, Mark Abbott, Arturo Barbachano-Guerrero, Efraín Beltrán-Ayala, Mercy J Borbor-Cordova, Washington B Cárdenas, and Cinthya Cueva. 2018. 'The burden of dengue fever and chikungunya in southern coastal Ecuador: epidemiology, clinical presentation, and phylogenetics from the first two years of a prospective study', The American journal of tropical medicine and hygiene, 98: 1444-59. |
| Sudeep, A. B., S. L. Hundekar, P. G. Jacob, R. Balasubramanian, V. A. Arankalle, and A. C. Mishra. 2011. 'Investigation of a Chikungunya-like illness in Tirunelveli district, Tamil Nadu, India 2009-2010', Trop Med Int Health, 16: 585-8. |
| Sutherland, L. J., A. A. Cash, Y. J. Huang, R. C. Sang, I. Malhotra, A. M. Moormann, C. L. King, S. C. Weaver, C. H. King, and A. D. LaBeaud. 2011. 'Serologic evidence of arboviral infections among humans in Kenya', Am J Trop Med Hyg, 85: 158-61. |
| Suwanmanee, S., P. Surasombatpattana, N. Soonthornworasiri, R. Hamel, P. Maneekan, D. Missé, and N. Luplertlop. 2018. 'Monitoring arbovirus in Thailand: Surveillance of dengue, chikungunya and zika virus, with a focus on coinfections', Acta Trop, 188: 244-50. |
| Sy, A. K., M. Saito-Obata, I. A. Medado, K. Tohma, C. Dapat, E. Segubre-Mercado, A. Tandoc, 3rd, S. Lupisan, and H. Oshitani. 2016. 'Molecular Characterization of Chikungunya Virus, Philippines, 2011-2013', Emerg Infect Dis, 22: 887-90. |
| Taraphdar, D., A. Sarkar, B. B. Mukhopadhyay, S. Chakrabarti, and S. Chatterjee. 2012. 'Rapid spread of chikungunya virus following its resurgence during 2006 in West Bengal, India', Trans R Soc Trop Med Hyg, 106: 160-6. |
| Taraphdar, D., and S. Chatterjee. 2015. 'Molecular characterization of chikungunya virus circulating in urban and rural areas of West Bengal, India after its re-emergence in 2006', Trans R Soc Trop Med Hyg, 109: 197-202. |
| Tauro, L. B., C. W. Cardoso, R. L. Souza, L. C. Nascimento, D. R. D. Santos, G. S. Campos, S. Sardi, O. B. D. Reis, M. G. Reis, U. Kitron, and G. S. Ribeiro. 2019. 'A localized outbreak of Chikungunya virus in Salvador, Bahia, Brazil', Mem Inst Oswaldo Cruz, 114: e180597. |
| Tavakoli, F., F. Rezaei, N. Z. Shafiei-Jandaghi, A. Shadab, and T. Mokhtari-Azad. 2020. 'Seroepidemiology of dengue and chikungunya fever in patients with rash and fever in Iran, 2017', Epidemiol Infect, 148: e42. |
| Theamboonlers, A., P. Rianthavorn, K. Praianantathavorn, N. Wuttirattanakowit, and Y. Poovorawan. 2009. 'Clinical and molecular characterization of chikungunya virus in South Thailand', Jpn J Infect Dis, 62: 303-5. |
| Tigoi, C., O. Lwande, B. Orindi, Z. Irura, J. Ongus, and R. Sang. 2015. 'Seroepidemiology of selected arboviruses in febrile patients visiting selected health facilities in the lake/river basin areas of Lake Baringo, Lake Naivasha, and Tana River, Kenya', Vector Borne Zoonotic Dis, 15: 124-32. |
| Tomashek, K. M., O. D. Lorenzi, D. A. Andújar-Pérez, B. C. Torres-Velásquez, E. A. Hunsperger, J. L. Munoz-Jordan, J. Perez-Padilla, A. Rivera, G. E. Gonzalez-Zeno, T. M. Sharp, R. L. Galloway, M. Glass Elrod, D. L. Mathis, M. S. Oberste, W. A. Nix, E. Henderson, J. McQuiston, J. Singleton, C. Kato, C. García Gubern, W. Santiago-Rivera, J. Cruz-Correa, R. Muns-Sosa, J. D. Ortiz-Rivera, G. Jiménez, I. E. Galarza, K. Horiuchi, H. S. Margolis, and L. I. Alvarado. 2017. 'Clinical and epidemiologic characteristics of dengue and other etiologic agents among patients with acute febrile illness, Puerto Rico, 2012-2015', PLoS Negl Trop Dis, 11: e0005859. |
| Triana-Vidal, Luz Elena, Mónica Andrea Morales-García, Maria Janeth Arango-Cárdenas, Marisol Badiel-Ocampo, and Daniel Elías Cuartas. 2019. 'Análisis de la distribución espacial y temporal de los virus del Dengue (2006-2017), Zika (2015-2017) y Chikungunya (2014-2017) en Colombia', Infectio, 23: 352-56. |
| Tun, M. M., K. Z. Thant, S. Inoue, T. Nabeshima, K. Aoki, A. K. Kyaw, T. Myint, T. Tar, K. T. Maung, D. Hayasaka, and K. Morita. 2014. 'Detection of east/central/south African genotype of chikungunya virus in Myanmar, 2010', Emerg Infect Dis, 20: 1378-81. |
| Uthappa, C. K., R. R. Allam, D. Gunti, C. Nalini, P. R. Udaragudi, G. P. Tadi, and M. V. Murhekar. 2015. 'Chikungunya outbreak in Atmakur village, Medak district, Telangana State, India', Indian J Med Res, 142: S108-10. |
| Vairo, F., A. Mammone, S. Lanini, E. Nicastri, C. Castilletti, F. Carletti, V. Puro, D. Di Lallo, V. Panella, D. Varrenti, P. Scaramozzino, A. di Caro, P. Scognamiglio, M. R. Capobianchi, and G. Ippolito. 2018. 'Local transmission of chikungunya in Rome and the Lazio region, Italy', PloS one, 13: e0208896. |
| van Genderen, F. T., I. Krishnadath, R. Sno, M. G. Grunberg, W. Zijlmans, and M. R. Adhin. 2016. 'First Chikungunya Outbreak in Suriname |
| Vega, F. L. R., J. M. T. Bezerra, R. F. C. Said, A. N. D. Gama Neto, E. C. Cotrim, D. Mendez, F. F. Amâncio, and M. Carneiro. 2019. 'Emergence of chikungunya and Zika in a municipality endemic to dengue, Santa Luzia, MG, Brazil, 2015-2017', Rev Soc Bras Med Trop, 52: e20180347. |
| Velasco, J. M., M. T. Valderama, M. N. Lopez, D. Chua, Jr., R. Latog, 2nd, V. Roque, Jr., J. Corpuz, C. Klungthong, P. Rodpradit, K. Hussem, Y. Poolpanichupatam, L. Macareo, S. Fernandez, and I. K. Yoon. 2015. 'Chikungunya Virus Infections Among Patients with Dengue-Like Illness at a Tertiary Care Hospital in the Philippines, 2012-2013', Am J Trop Med Hyg, 93: 1318-24. |
| Vieira, D. S., M. R. Zambenedetti, L. Requião, I. A. Borghetti, L. K. S. Luna, A. O. D. Santos, R. L. M. Taborda, D. B. Pereira, M. A. Krieger, J. M. V. Salcedo, and R. C. P. Rampazzo. 2019. 'Epidemiological profile of Zika, Dengue and Chikungunya virus infections identified by medical and molecular evaluations in Rondonia, Brazil', Rev Inst Med Trop Sao Paulo, 61: e40. |
| Vilibic-Cavlek, T., I. Pem-Novosel, B. Kaic, A. Babić-Erceg, J. Kucinar, A. Klobucar, A. Medic, D. Pahor, K. Barac-Juretic, and I. Gjenero-Margan. 2015. 'Seroprevalence and entomological study on Chikungunya virus at the Croatian littoral', Acta Microbiol Immunol Hung, 62: 199-206. |
| Vongpunsawad, Sompong, Duangnapa Intharasongkroh, Thanunrat Thongmee, and Yong Poovorawan. 2017. 'Seroprevalence of antibodies to dengue and chikungunya viruses in Thailand', PloS one, 12. |
| Wangchuk, S., P. Chinnawirotpisan, T. Dorji, T. Tobgay, T. Dorji, I. K. Yoon, and S. Fernandez. 2013. 'Chikungunya fever outbreak, Bhutan, 2012', Emerg Infect Dis, 19: 1681-4. |
| Wanlapakorn, N., T. Thongmee, P. Linsuwanon, P. Chattakul, S. Vongpunsawad, S. Payungporn, and Y. Poovorawan. 2014. 'Chikungunya outbreak in Bueng Kan Province, Thailand, 2013', Emerg Infect Dis, 20: 1404-6. |
| White, S. K., C. Mavian, M. A. Elbadry, V. M. Beau De Rochars, T. Paisie, T. Telisma, M. Salemi, J. A. Lednicky, and J. G. Morris, Jr. 2018. 'Detection and phylogenetic characterization of arbovirus dual-infections among persons during a chikungunya fever outbreak, Haiti 2014', PLoS Negl Trop Dis, 12: e0006505. |
| World Health Organization. 2006. "Chikungunya and Dengue in the south west Indian Ocean." In. |
| World Health Organization. 2006. "Chikungunya in India." In. |
| World Health Organization. 2013. "Chikungunya in the French part of the Caribbean isle of Saint Martin." In. |
| Chikungunya: case definitions for acute, atypical and chronic cases. Conclusions of an expert consultation, Managua, Nicaragua, 20-21 May 2015'. 2015. Wkly Epidemiol Rec, 90: 410-4. |
| World Health Organization. 2015. "Chikungunya – Senegal." In. |
| World Health Organization. 2016. "Chikungunya – Argentina." In. |
| World Health Organization. 2016. "Chikungunya – United States of America." In. |
| World Health Organization. 2017. "Chikungunya – France." In. |
| World Health Organization. 2017. "Chikungunya – Italy (update)." In. |
| World Health Organization. 2018. "Chikungunya – Sudan." In. |
| World Health Organization, Regional Office for Africa. 2018. "Chikungunya in Kenya." In Outbreaks and Emergencies Bulletin. WHO Regional Office for Africa. |
| World Health Organization. 2019. "Chikungunya in Congo." In Outbreaks and Emergencies Bulletin, 5. WHO Regional Office for Africa. |
| World Health Organization. 2019. "Chikungunya outbreak in Ethiopia." In Outbreaks and Emergencies Bulletin, 6. WHO Regional Office for Africa. |
| Wittlin, Bernardo Bastos. 2018. 'Prevalência sorológica de infecções por dengue, zika e chikungunya vírus em gestantes atendidas numa maternidade pública, no município de Nova Iguaçu, Baixada Fluminense': xiii,58-xiii,58. |
| Wu, D., J. Wu, Q. Zhang, H. Zhong, C. Ke, X. Deng, D. Guan, H. Li, Y. Zhang, H. Zhou, J. He, L. Li, and X. Yang. 2012. 'Chikungunya outbreak in Guangdong Province, China, 2010', Emerg Infect Dis, 18: 493-5. |
| Wu, D., Y. Zhang, Q. Zhouhui, J. Kou, W. Liang, H. Zhang, C. Monagin, Q. Zhang, W. Li, H. Zhong, J. He, H. Li, S. Cai, C. Ke, and J. Lin. 2013. 'Chikungunya virus with E1-A226V mutation causing two outbreaks in 2010, Guangdong, China', Virol J, 10: 174. |
| Yadav, Pragya D, Deepak Y Patil, and Devendra T Mourya. 2018. 'Positivity of dengue and chikungunya among Crimean–Congo hemorrhagic fever-negative cases in India: 2013–2016'. |
| Yaqub, T., M. Z. Shabbir, N. Mukhtar, Z. Tahir, T. Abbas, E. Amir, and G. Defang. 2017. 'Detection of selected arboviral infections in patients with history of persistent fever in Pakistan', Acta Trop, 176: 34-38. |
| Yergolkar, P. N., B. V. Tandale, V. A. Arankalle, P. S. Sathe, A. B. Sudeep, S. S. Gandhe, M. D. Gokhle, G. P. Jacob, S. L. Hundekar, and A. C. Mishra. 2006. 'Chikungunya outbreaks caused by African genotype, India', Emerg Infect Dis, 12: 1580-3. |
| Yoon, In-Kyu, Maria Theresa Alera, Catherine B Lago, Ilya A Tac-An, Daisy Villa, Stefan Fernandez, Butsaya Thaisomboonsuk, Chonticha Klungthong, Jens W Levy, and John Mark Velasco. 2015. 'High rate of subclinical chikungunya virus infection and association of neutralizing antibody with protection in a prospective cohort in the Philippines', PLoS neglected tropical diseases, 9. |
| Yoosuf, A. A., I. Shiham, A. J. Mohamed, G. Ali, J. M. Luna, R. Pandav, G. N. Gongal, A. Nisaluk, R. G. Jarman, and R. V. Gibbons. 2009. 'First report of chikungunya from the Maldives', Trans R Soc Trop Med Hyg, 103: 192-6. |
| Yusof, Mohd Apandi, L Sau Kuen, Norfaezah Adnan, NI Razak, and L Ahmad Zamri. 2011. 'Epidemiology and molecular characterization of chikungunya virus involved in the 2008 to 2009 outbreak in Malaysia', Journal of General and Molecular Virology, 3: 35-42. |
| Yusoff, A. F., A. N. Mustafa, H. M. Husaain, W. M. Hamzah, A. M. Yusof, R. Harun, and F. N. Abdullah. 2013. 'The assessment of risk factors for the Central/East African Genotype of chikungunya virus infections in the state of Kelantan: a case control study in Malaysia', BMC Infect Dis, 13: 211. |
| Zambrano, L. I., M. Sierra, B. Lara, I. Rodríguez-Núñez, M. T. Medina, C. O. Lozada-Riascos, and A. J. Rodríguez-Morales. 2017. 'Estimating and mapping the incidence of dengue and chikungunya in Honduras during 2015 using Geographic Information Systems (GIS)', J Infect Public Health, 10: 446-56. |
