# Supplementary material for: The global epidemiology of chikungunya from 1999 to 2020: A systematic literature review to inform the development and introduction of vaccines
Source: PLoS Negl Trop Dis. 2022 Jan 12;16(1):e0010069. doi: 10.1371/journal.pntd.0010069 (PMC8789145; doi:10.1371/journal.pntd.0010069)
Supplement: S5 Table — We identified 19 examples of surveillance systems in place across 12 geographical areas from MoH websites, as outlined in the methodology. (DOCX) [file pntd.0010069.s007.docx]

S5 Table: Surveillance systems in place, by country***.*** *We identified 19 examples of surveillance systems in place across 12 geographical areas from MoH websites, as outlined in the methodology.*

| **Country** | **Geographical coverage (national, regional)** | **Population covered (all ages, specific age groups, special populations)** | **Type of surveillance (active, passive)** | **Type of reporting (compulsory, voluntary, sentinel network)** | **Case definition used** | **Laboratory diagnosis** | **Representativeness** | **Time period or start date** | **References** |
| --- | --- | --- | --- | --- | --- | --- | --- | --- | --- |
| **Africa** | | | | | | | | | |
| Gabon | National | all ages | active | Sentinel  Physicians; labs | An acute febrile syndrome was characterized by acute fever(>38.5°C) and≥1 of the following symptoms: arthralgia, myalgia, headache, rash, asthenia, nausea, vomiting, diarrhea, jaundice, or bleeding. | RT-PCR | not assessed | Sept 2007-Aug 2010 | [[1) |
| Mayotte, Comoros archipelago | Regional | all ages | passive | Voluntary  Health care providers and hospitals | Suspected case: any person with incapacitating polyarthralgia and a history of sudden onset of fever (body temperature ≥38.5 ◦C) possibly associated with myalgia, headache, skin rash or conjunctival injection in the absence of Plasmodium species infection checked by the rapid diagnostic test (OptiMAL, Flow Inc.,Portland, OR, USA).  Confirmed case: suspected case with at least one of the following laboratory criteria: (i) detection of the CHIK virus genome in a fluid sample (blood, exudate, cerebrospinal fluid) by RT-PCR; (ii) demonstration of specific anti-CHIK virus IgM in a fluid sample regardless of the presence of specific anti-CHIK virus IgG by ELISA. | MAC-ELISA; RT-PCR | not assessed | since 2005 | (2) |
| Mayotte, Comoros archipelago |  | maternofetal cases | active | Voluntary  Hospital based | Maternoneonatal CHIK fever case is defined as a new-born with laboratory confirmed CHIK fever within the first 9 days of life whom the mother had had confirmed CHIK fever during the last month of pregnancy. ‘Severe form’ was defined as laboratory confirmed patients over 9 days old who had at least one organic involvement other than articular manifestation and requiring the maintenance of at least one vital function | MAC-ELISA; RT-PCR | not assessed | since 2006 | (2) |
| Reunion island | National | all ages >10 days | active | NA | Fever, arthralgia |  | not assessed |  | (3) |
| Reunion island | National | all ages | active | Sentinel  Physicians; labs; GPs; patients | Suspected case: rapid onset of fever over 38.5°C with incapacitating joint pain  Confirmed by the detection of anti-chikungunya virus IgM and/or detection of viral ARN by RT-PCR or virus isolation | RT-PCR | not assessed | ongoing | (4) |
| Reunion island | National | all ages | active | Sentinel  Labs, private physicians, patients | Suspected case: Sudden onset of fever > 38.5°C accompanied by incapacitating joint pain.  Confirmed case: positive IgM serologic results and/or the chikungunya genome detected by reverse chain reaction (RT-PCR). | RT-PCR | yes | since Mar-2005 | (5) |
| **Asia** | | | | | | | | | |
| India | National | all ages | active | Sentinel | Chikungunya suggesting syndrome (fever, arthralgia, myalgia, rash), definition by National CDC of India 2006 | MAC ELISA | not assessed |  | (6) |
| India | National | all ages | active | lab based | Acute Febrile Illness with rash, arthralgia or haemorrhagic manifestations are investigated for dengue as well as chikungunya | MAC ELISA | Predominantly urban areas | since 2013 | (7) |
| India | National | all ages | active | local health units | Patients with fever or arthralgia presented at medical camp on or after June 20, 2006 | ELISA | urban | ongoing | (8) |
| Singapore | National | all ages | active | Sentinel  Lab based | Acute febrile illness of at least 37.5C, with or without joint pain, and whose blood sample was either tested positive for CHIKV by RT-PCR or showed a four-fold rise in anti-CHIKV IgG antibody titres from acute and convalescent samples taken at least 14 days apart; clinically compatible cases with a positive anti-CHIKV IgM result if they were epidemiologically linked to a laboratory-confirmed case | RT-PCR | not assessed | Dec-06 | (9) |
| Singapore | National | all ages | passive | Compulsory | Acute febrile illness of at least 37.5C, with or without joint pain, and whose blood sample was either tested positive for CHIKV by RT-PCR or showed a four-fold rise in anti-CHIKV IgG antibody titres from acute and convalescent samples taken at least 14 days apart; clinically compatible cases with a positive anti-CHIKV IgM result if they were epidemiologically linked to a laboratory-confirmed case | RT-PCR | not assessed | Dec-08 | (9) |
| **South America** | | | | | | | | | |
| Brazil | Regional | all ages | active | Compulsory | Suspected case: acute onset of fever, severe arthralgia and/or arthritis, residing or having visited epidemic areas within the last 15 days before symptom onset  Confirmed case: lab confirmation through cell culture, RNA detection or serology. | RT-PCR | not assessed | ongoing | (10) |
| Colombia | National | all ages | active | Compulsory | Clinically suspected cases with presence of a rash and an elevation of axillary body temperature greater than 37.2 °C; non-purulent conjunctivitis or conjunctival hyperaemia, arthralgia or myalgias and headache or general malaise | rRT-PCR | not assessed | ongoing | (11) |
| Jamaica | National | all ages | active and passive | Sentinel  Sites at primary health care centers, and all major hospitals  Medical practitioners | Suspected case: fever of acute onset of > 101.3°F (38.5°C) *and* severe arthralgia (or arthritis) not explained by other medical conditions in a person who resides in or had visited an epidemic or endemic area within 2 weeks prior to symptom onset  Confirmed case: a suspected case with a positive result by any of the following CHIKV specific laboratory tests: viral isolation, detection of viral RNA by RT-PCR, detection of IgM in a single serum sample (collected during acute or convalescent phase), or 4-fold increase in CHIKV-specific antibody titers (samples collected at least 2 – 3 weeks apart). | RT-PCR | not assessed | ongoing | (12) |
| Suriname | National | all ages | active | Voluntary  Lab based | Suspected case: fever >38.5C and severe arthralgia/arthritis not explained by other medical conditions who is residing or has visited epidemic areas within 15 days prior to onset of symptoms.  Confirmed case: suspected patient meeting laboratory confirmation criteria, which are either virus culture, RT-PCR, IgM antibody assay in single sample, or four-fold increase in CHIKV-specific antibody titers IgG (Pan American Health Organization 2015). | ELISA, FRNT, RT-PCR | not assessed | From Oct 2014 | (13) |
| **North America** | | | | | | | | | |
| Puerto Rico | National | all ages | passive | Compulsory  Physician based | Clinically suspected (no precise definition is noted) | MAC ELISA, RT-PCR | not assessed | since the late 1960s | (14) |
| Puerto Rico | National | all ages | active | Compulsory  Health care facility based | Febrile patients suspected for the infection and presenting at a health care facility | MAC ELISA, RT-PCR | not assessed | since 2012 | (14) |
| Puerto Rico | National | all ages | combined | Compulsory | Premortem lab criteria  Forensic physicians reports combined with pre- and post-mortem serum samples | RT-PCR | not assessed | since 2010 | (14) |
| USA | National | all ages | passive | Compulsory  Local public health departments  Hospitals  Laboratories  Health care providers | CDC standard definitions | not available | not assessed | ongoing | (15) |

**S5 Table references**

1. Caron M, Paupy C, Grard G, Becquart P, Mombo I, Nso BB, et al. Recent introduction and rapid dissemination of Chikungunya virus and Dengue virus serotype 2 associated with human and mosquito coinfections in Gabon, central Africa. Clin Infect Dis. 2012;55(6):e45-53.

2. Sissoko D, Malvy D, Giry C, Delmas G, Paquet C, Gabrie P, et al. Outbreak of Chikungunya fever in Mayotte, Comoros archipelago, 2005-2006. Trans R Soc Trop Med Hyg. 2008;102(8):780-6.

3. Dominguez M, Economopoulou A. Surveillance active des formes émergentes hospitalières de chikungunya. La Réunion, avril. 2005.

4. Paquet C, Quatresous I, Solet J, Sissoko D, Renault P, Pierre V, et al. Chikungunya outbreak in Reunion: epidemiology and surveillance, 2005 to early January 2006. Weekly releases (1997–2007). 2006;11(5):2891.

5. Renault P, Solet J-L, Sissoko D, Balleydier E, Larrieu S, Filleul L, et al. A major epidemic of chikungunya virus infection on Reunion Island, France, 2005–2006. The American journal of tropical medicine and hygiene. 2007;77(4):727-31.

6. Chakravarti A, Malik S, Tiwari S, Ashraf A. A study of Chikungunya outbreak in Delhi. J Commun Dis. 2011;43(4):259-63.

7. Murhekar M, Kanagasabai K, Shete V, Joshua V, Ravi M, Kirubakaran BK, et al. Epidemiology of chikungunya based on laboratory surveillance data-India, 2016-2018. Trans R Soc Trop Med Hyg. 2019;113(5):259-62.

8. Seyler T, Sakdapolrak P, Prasad SS, Dhanraj R. A chikungunya outbreak in the metropolis of Chennai, India, 2006. J Environ Health. 2012;74(6):8-13; quiz 64.

9. Ho K, Ang LW, Tan BH, Tang CS, Ooi PL, James L, et al. Epidemiology and control of chikungunya fever in Singapore. J Infect. 2011;62(4):263-70.

10. Naveca FG, Claro I, Giovanetti M, de Jesus JG, Xavier J, de Melo Iani FC, et al. Genomic, epidemiological and digital surveillance of Chikungunya virus in the Brazilian Amazon. PLoS neglected tropical diseases. 2019;13(3):e0007065.

11. Mercado-Reyes M, Acosta-Reyes J, Navarro-Lechuga E, Corchuelo S, Rico A, Parra E, et al. Dengue, chikungunya and Zika virus coinfection: results of the national surveillance during the Zika epidemic in Colombia. Epidemiology & Infection. 2019;147.

12. Duncan J, Gordon-Johnson KA, K.Tulloch-Reid M, Cunningham-Myrie C, Ernst K, McMorris N, et al. Chikungunya: important lessons from the Jamaican experience. Revista Panamericana de Salud Pública. 2017;41:e60.

13. Goeijenbier M, Aron G, Anfasa F, Lundkvist Å, Verner-Carlsson J, Reusken CB, et al. Emerging Viruses in the Republic of Suriname: Retrospective and Prospective Study into Chikungunya Circulation and Suspicion of Human Hantavirus Infections, 2008-2012 and 2014. Vector Borne Zoonotic Dis. 2015;15(10):611-8.

14. Sharp TM, Ryff KR, Alvarado L, Shieh W-J, Zaki SR, Margolis HS, et al. Surveillance for chikungunya and dengue during the first year of chikungunya virus circulation in Puerto Rico. The Journal of infectious diseases. 2016;214:S475-S81.

15. Rosenberg R, Lindsey NP, Fischer M, Gregory CJ, Hinckley AF, Mead PS, et al. Vital signs: trends in reported vectorborne disease cases—United States and Territories, 2004–2016. Morbidity and Mortality Weekly Report. 2018;67(17):496.
