# Supplementary material for: The global epidemiology of chikungunya from 1999 to 2020: A systematic literature review to inform the development and introduction of vaccines
Source: PLoS Negl Trop Dis. 2022 Jan 12;16(1):e0010069. doi: 10.1371/journal.pntd.0010069 (PMC8789145; doi:10.1371/journal.pntd.0010069)
Supplement: Peer review history

# Peer review: Decision Letter 0

**DOI:** 10.1371/journal.pntd.0010069.r001

**Authors:** Pedro F. C. Vasconcelos

7 Dec 2021

Dear L'Azou Jackson,

We are pleased to inform you that your manuscript 'The global epidemiology of chikungunya from 1999 to 2020: a systematic literature review to inform the development and introduction of vaccines' has been provisionally accepted for publication in PLOS Neglected Tropical Diseases.

Before your manuscript can be formally accepted you will need to complete some formatting changes, which you will receive in a follow up email. A member of our team will be in touch with a set of requests.

Please note that your manuscript will not be scheduled for publication until you have made the required changes, so a swift response is appreciated.

IMPORTANT: The editorial review process is now complete. PLOS will only permit corrections to spelling, formatting or significant scientific errors from this point onwards. Requests for major changes, or any which affect the scientific understanding of your work, will cause delays to the publication date of your manuscript.

Should you, your institution's press office or the journal office choose to press release your paper, you will automatically be opted out of early publication. We ask that you notify us now if you or your institution is planning to press release the article. All press must be co-ordinated with PLOS.

Thank you again for supporting Open Access publishing; we are looking forward to publishing your work in PLOS Neglected Tropical Diseases.

Best regards,

Pedro F. C. Vasconcelos

Deputy Editor

PLOS Neglected Tropical Diseases

Pedro Vasconcelos

Deputy Editor

PLOS Neglected Tropical Diseases

***********************************************************

Reviewer's Responses to Questions

**Key Review Criteria Required for Acceptance?**

As you describe the new analyses required for acceptance, please consider the following:

**Methods**

-Are the objectives of the study clearly articulated with a clear testable hypothesis stated?

-Is the study design appropriate to address the stated objectives?

-Is the population clearly described and appropriate for the hypothesis being tested?

-Is the sample size sufficient to ensure adequate power to address the hypothesis being tested?

-Were correct statistical analysis used to support conclusions?

-Are there concerns about ethical or regulatory requirements being met?

Reviewer #1: The objective of the SR is clear. The authors use an adequate and transparent methodology. The search strategy in reference sources is limited to MEDLINE. This could have trouble identifying other items. They also report searches in other sources of information, including WHO, LILACS and others.

To improve I recommend:

1. It is not clear from the flow chart (figure 1) whether the 1497 are results from all the other sources mentioned. This needs to be clarified. How many are from other sources databases? How many are really gray literature?

2. In appendix 2, the number of references identified in each source must be put.

3. Perform a search on Embase and update the results. As the authors well mention, Europe has been affected by CHIKV and it is likely that unidentified articles have remained

I welcome the decision not to combine the results in a meta-analysis. It was not desirable given the high heterogeneity between the studies.

* Endnote is a registered trademark.

Reviewer #2: The authors analyzed 371 published studies that met the eligibility criteria. As this is a descriptive review, the Authors basically summarized their results in table format. Methods and groups for analysis were well described.

Reviewer #3: The objective and purpose of the paper are clearing stated. The paper used a systematic review of literature on Chikungunya disease over a 20-year period. The study used the right methods in obtaining the necessary data for the paper, and these are clearly explained under the methods section.

The author showed the various search string used for obtaining the relevant literature to support the study. There is an explanation for works included in the study and those that were excluded. The methods also explain how data were analyzed and presented at the results side.

**********

**Results**

-Does the analysis presented match the analysis plan?

-Are the results clearly and completely presented?

-Are the figures (Tables, Images) of sufficient quality for clarity?

Reviewer #1: Line 168: check an error (Error! Reference source not found.)

The results are well presented. The tables are extensive but allow to have the information ordered and clear by region.

Reviewer #2: Results were clearly described and matched the analysis plans.

Reviewer #3: Results following the objective of the study and the analysis plan. They were clearly presented and were written in a manner that helps the reader to follow through and understand each step presented. Charts, figures and table used are clear, appropriate and representative enough.

**********

**Conclusions**

-Are the conclusions supported by the data presented?

-Are the limitations of analysis clearly described?

-Do the authors discuss how these data can be helpful to advance our understanding of the topic under study?

-Is public health relevance addressed?

Reviewer #1: I think this is not a limitation but an error of the authors: Line 448 "The search terms may not have been exhaustive. Please review your search strategy and be clear enough to improve it. In methods I recommended including Embase, but IF the Authors are not convinced of their own strategy, these results would not be valid.

Reviewer #2: Conclusions are consistent with the results presented in the study and study's limitations are well described as well as possible relevance for public health.

Reviewer #3: The study is appropriate in helping us understand the epidemiology of the chikungunya disease. It also presents to use the available data, reports, prevalence and other dynamics which would aid in the development of vaccines to combat the disease. This could help with an alternative source of information for policy and planning purposes in activities targeted at the combating disease.

The author detailed out the limitations of the study and the methods used, and proposed relevant recommendations. The conclusion affirms the data and the results presented.

**********

**Editorial and Data Presentation Modifications?**

Use this section for editorial suggestions as well as relatively minor modifications of existing data that would enhance clarity. If the only modifications needed are minor and/or editorial, you may wish to recommend “Minor Revision” or “Accept”.

Reviewer #1: Table 1:

Change en Colombia: North (Corozal & Ovejjas, Sucre) by North (Corozal & Ovejas, Sucre).

They must erase a j to Ovejjas.

Reviewer #2: Authors should correct some typos in lines 168, 183, and 230 in the Results section.

Reviewer #3: Accept

**********

**Summary and General Comments**

Use this section to provide overall comments, discuss strengths/weaknesses of the study, novelty, significance, general execution and scholarship. You may also include additional comments for the author, including concerns about dual publication, research ethics, or publication ethics. If requesting major revision, please articulate the new experiments that are needed.

Reviewer #1: I am grateful for the invitation to evaluate the paper "The global epidemiology of chikungunya from 1999 to 2020: a systematic literature review to inform the development and introduction of vaccines". It is a great initiative on the part of the authors to dare to summarize in a systematic way the information published on the epidemiology of the CHKV.

In a global assessment it was a great job. The authors use an appropriate methodology. It is completely transparent, including the registration in PROSPERO (attention: you must update it). But they are also aware of the challenge they took and unfortunately of the weakness of the results. The latter as a consequence of the great variability and limitations of the retrieved studies.

Despite this, the results allow a vision of the situation in recent decades regarding the behavior of the CHIKV, and clearly inform their findings. And in the end they provide recommendations for what should be continued in the CHIKV research.

Reviewer #2: The study aims to provide an in-depth analysis of the epidemiology of CHIKV infection over the last 20 years and to highlight current knowledge gaps and major challenges for assessing candidate vaccines and the feasibility to implement vaccine efficacy studies.

The authors analyzed 371 published studies that met the eligibility criteria. As this is a descriptive review, the Authors basically summarized their results in table format. Methods and groups for analysis were well described. Results were clearly described and matched the analysis plans. Conclusions are consistent with the results presented in the study and study's limitations are well described as well as possible relevance for public health.

Reviewer #3: The strong aspect of the study is the ability to use data over a period of time spanning over 20 years in identifying the trends and changing patterns of the disease globally. The study identified appropriate methods in presenting descriptive analysis of the relevant publications on the disease, which also helped the author in achieving the overall purpose of the study. Review of existing literature also helped to gather evidence from various sources to give a bigger picture of the situation of chikungunya disease.

The author identified major limitations such as missing some likely outbreaks, inadequate search terms and publications which are done after the study period. However, with the use of appropriate methodology, the effect of such limitations on the outcome of the study is minimal.

Overall, the paper is well writing, and it satisfies my consideration for acceptance for publication.

**********

PLOS authors have the option to publish the peer review history of their article (what does this mean?). If published, this will include your full peer review and any attached files.

If you choose “no”, your identity will remain anonymous but your review may still be made public.

**Do you want your identity to be public for this peer review?** For information about this choice, including consent withdrawal, please see our Privacy Policy.

Reviewer #1: No

Reviewer #2: No

Reviewer #3: **Yes: **Richard Amoako

# Editor report: Acceptance letter

**DOI:** 10.1371/journal.pntd.0010069.r002

**Authors:** Pedro F. C. Vasconcelos

7 Jan 2022

Dear L'Azou Jackson,

We are delighted to inform you that your manuscript, "The global epidemiology of chikungunya from 1999 to 2020: a systematic literature review to inform the development and introduction of vaccines," has been formally accepted for publication in PLOS Neglected Tropical Diseases.

We have now passed your article onto the PLOS Production Department who will complete the rest of the publication process. All authors will receive a confirmation email upon publication.

The corresponding author will soon be receiving a typeset proof for review, to ensure errors have not been introduced during production. Please review the PDF proof of your manuscript carefully, as this is the last chance to correct any scientific or type-setting errors. Please note that major changes, or those which affect the scientific understanding of the work, will likely cause delays to the publication date of your manuscript. Note: Proofs for Front Matter articles (Editorial, Viewpoint, Symposium, Review, etc...) are generated on a different schedule and may not be made available as quickly.

Soon after your final files are uploaded, the early version of your manuscript will be published online unless you opted out of this process. The date of the early version will be your article's publication date. The final article will be published to the same URL, and all versions of the paper will be accessible to readers.

Thank you again for supporting open-access publishing; we are looking forward to publishing your work in PLOS Neglected Tropical Diseases. 

Best regards,

Shaden Kamhawi

co-Editor-in-Chief

PLOS Neglected Tropical Diseases

Paul Brindley

co-Editor-in-Chief

PLOS Neglected Tropical Diseases
